# Women’s subsistence strategies predict fertility across cultures, but context matters

**DOI:** 10.1073/pnas.2318181121

**Published:** 2024-02-12

**Authors:** Abigail E. Page, Erik J. Ringen, Jeremy Koster, Monique Borgerhoff Mulder, Karen Kramer, Mary K. Shenk, Jonathan Stieglitz, Kathrine Starkweather, John P. Ziker, Adam H. Boyette, Heidi Colleran, Cristina Moya, Juan Du, Siobhán M. Mattison, Russell Greaves, Chun-Yi Sum, Ruizhe Liu, Sheina Lew-Levy, Francy Kiabiya Ntamboudila, Sean Prall, Mary C. Towner, Tami Blumenfield, Andrea B. Migliano, Daniel Major-Smith, Mark Dyble, Gul Deniz Salali, Nikhil Chaudhary, Inez E. Derkx, Cody T. Ross, Brooke A. Scelza, Michael D. Gurven, Bruce P. Winterhalder, Carmen Cortez, Luis Pacheco-Cobos, Ryan Schacht, Shane J. Macfarlan, Donna Leonetti, Jennifer C. French, Nurul Alam, Fatema tuz Zohora, Hillard S. Kaplan, Paul L. Hooper, Rebecca Sear

**Affiliations:** ^a^Division of Psychology, Brunel University of London, London UB8 3PN, United Kingdom; ^b^Department of Population Health, London School of Hygiene and Tropical Medicine, London WC1E 7HT, United Kingdom; ^c^University of Zürich, Zürich 8050, Switzerland; ^d^Department of Comparative Language Science, University of Zürich, Zürich 8050, Switzerland; ^e^Center for the Interdisciplinary Study of Language Evolution, University of Zürich, Zürich 8050, Switzerland; ^f^Department of Human Behavior, Ecology and Culture, Max Planck Institute for Evolutionary Anthropology, Leipzig 04103, Germany; ^g^Department of Anthropology, University of California, Davis, CA 95616; ^h^Department of Anthropology, University of Utah, Salt Lake City, UT 84112; ^i^Department of Anthropology, Penn State College of the Liberal Arts, State College, PA 16801; ^j^Institute for Advanced Study in Toulouse, Universite Toulouse 1 Capitole, Toulouse 31080, France; ^k^Department of Anthropology, University of Illinois Chicago, Chicago, IL 60607; ^l^Department of Anthropology, Boise State University, Boise, ID 83725; ^m^State Key Laboratory of Grassland Agro-Ecosystem, College of Ecology, Lanzhou University, Lanzhou 730000, China; ^n^Department of Anthropology, The University of New Mexico, Albuquerque, NM 87106; ^o^Maxwell Museum of Anthropology, University of New Mexico, Albuquerque, NM 87106; ^p^Anthropology Department, Boston University, Boston, MA 02215; ^q^Department of Psychology, Durham University, Durham DH1 3LE, United Kingdom; ^r^Faculté des Lettres, Arts, et Sciences Humaines, Département d’anthropologie, Marien Ngouabi University, Brazzaville, Congo; ^s^Department of Anthropology, University of Missouri, Columbia, MO 65201; ^t^Department of Integrative Biology, Oklahoma State University, Stillwater, OK 74078; ^u^Department of Anthropology, Universität Zürich, Zürich 8050, Switzerland; ^v^Population Health Sciences, Bristol Medical School, University of Bristol, Bristol BS8 2PS, United Kingdom; ^w^Department of Archaeology, University of Cambridge, Cambridge CB2 3DZ, United Kingdom; ^x^Department of Anthropology, University College London, London WC1H 0BW, United Kingdom; ^y^Department of Anthropology, University of California, Los Angeles, CA 90095; ^z^Department of Anthropology, University of California, Santa Barbara, CA 93106; ^aa^Community Agroecology Network, Santa Cruz, CA 95064; ^bb^Facultad de Biología–Xalapa, Universidad Veracruzana, Zalapa-Enriquez 91090, México; ^cc^Department of Anthropology, East Carolina University, Greenville, NC 27858; ^dd^Department of Anthropology, University of Washington, Settle, WA 98105; ^ee^Department of Archaeology, Classics and Egyptology, University of Liverpool, Liverpool L69 7WZ, United Kingdom; ^ff^Health Systems and Population Studies Division, International Centre for Diarrheal Disease Research, Bangladesh, Dhaka 1213, Bangladesh

**Keywords:** fertility, subsistence-based populations, cross-cultural analysis, anthropological demography, demographic transition

## Abstract

While it is commonly assumed that farmers have higher, and foragers lower, fertility compared to populations practicing other forms of subsistence, robust supportive evidence is lacking. We tested whether subsistence activities—incorporating market integration—are associated with fertility in 10,250 women from 27 small-scale societies and found considerable variation in fertility. This variation did not align with group-level subsistence typologies. Societies labeled as “farmers” did not have higher fertility than others, while “foragers” did not have lower fertility. However, at the individual level, we found strong evidence that fertility was positively associated with farming and moderate evidence of a negative relationship between foraging and fertility. Markers of market integration were strongly negatively correlated with fertility. Despite strong cross-cultural evidence, these relationships were not consistent in all populations, highlighting the importance of the socioecological context, which likely influences the diverse mechanisms driving the relationship between fertility and subsistence.

## Significance

While it is commonly believed that farming is linked to higher fertility, our analysis of 27 societies revealed that these relationships are not apparent between populations. At the individual level, cross-culturally, there is strong evidence associating fertility with increased engagement in farming. Nonetheless, these results are not consistent across societies, representing different socioecological contexts. Our results emphasize that subsistence alone cannot predict an individual’s fertility, highlighting the significant influence of socioecological contexts. This means that the broader environment, cultural norms, and local dynamics play a pivotal role in fertility, even when individuals engage in similar subsistence strategies. Our findings caution against oversimplifying the relationship between subsistence and fertility and underscore the importance of context in understanding human reproductive behavior.

There is a long-standing assumption across anthropology, demography, and archaeology that human fertility (definitions in Table 1) is higher in farming populations and lower in hunter–gatherer populations due to assumed differences in energetic status associated with different modes of food production (1–4). The strength of evidence for this assumption is, however, weak: Several comparative anthropological studies have explicitly tested this in contemporary subsistence-based populations (5–8) but found little, or inconsistent, differences in average total fertility by subsistence type. Moreover, any analysis of contemporary populations needs to robustly address the shifts to waged labor markets which have happened cross-culturally since few populations are now purely “subsistence” based, relying only on the food they themselves produce. To answer the question of whether subsistence activities are associated with fertility, we here provide a robust, individual-level analysis of a large, cross-cultural dataset of contemporary societies, which incorporates measures of market integration.

**Table 1.** Key terms

Term	Definition
Subsistence typology	Overarching terms to which define a group based on their perceived main economic activity. Often divided into foragers, horticulturalists, agriculturalists, fishers, and pastoralists.
Agriculturalists	Depend mostly (56% or more) on intensive agriculture. Intensive agriculture means a variety of techniques are used so that fields can be permanently cultivated. These techniques can include irrigation, terracing, crop rotation, plows, and/or some sort of agriculture.
Foragers	Also called hunter–gatherers; depend almost entirely (86% or more) on hunting, fishing, and gathering for subsistence
Horticulturalists	Horticulturalists depend mostly (56% or more) on simple agriculture (extensive or horticulture) with less usage of permanent field cultivation and/or irrigation.
Fishers	Engage heavily in fishing without engaging in other hunting and gathering tasks associated with foragers.
Pastoralists	Depend mostly (56% or more) on herding of domesticated animals.
Market integration	Market integration is the shift from subsistence-based economic activities to cash-based ones, which is reflected in changes in production, consumption, and acculturation.
Energetics	Study of energy flows, including intake (calorific consumption), expenditure (physical activity), and energy balance. A positive energetic balance is where individuals have higher intake than energy expenditure. Has implications for the allocation of metabolic energy to reproduction.
Fertility	Many operationalizations of this term exist. Here, we are referring to the total number of children born at the end of a woman’s reproductive career.

## The Advantages of Using Individual-Level Data

Previous analyses relied on population-level subsistence typologies which suffer from several interrelated issues. For instance, a study of 57 small-scale populations (5) found that the average fertility of agriculturalists was significantly higher than that of nonagriculturalists in line with expectations. However, this result was driven by the lower fertility of horticulturalists not foragers (agriculturalists and horticulturalists are both farmers, but the former engage in more intensive farming—see Table 1). More importantly, reliance on population-level subsistence types reduces sample size and statistical power, increasing the likelihood of a Type II error (i.e., a false negative), meaning such results need to be interpreted with caution.

Beyond methodological concerns, there are also theoretical reasons why population-level analysis may provide a weak test of whether subsistence strategy affects fertility. The most common explanation for why subsistence may affect fertility is through individual-level, energetic pathways (9). Individual women’s energetic balance may differ when engaging in different subsistence strategies due to alterations in diet (10), work (2, 11), childcare (11), and mobility (12). A more accurate exploration of the relationship between fertility and subsistence requires the use of individual-level measures of fertility and subsistence to avoid the ecological fallacy—inferring individual-level relationships based solely on group-level data (13).

Individual-level data also overcome limitations associated with subsistence typologies, i.e., assuming all individuals adopt very similar lifestyles within populations, and across all populations with the same subsistence type. Despite the impression given by the thresholds presented in Table 1, subsistence typologies are not bounded entities, identical across cultural and geographic range, but are open to interpretation. Taking the example of hunter–gatherers: Within the archaeological and anthropological record, there is clear documentation of mixed subsistence strategies in many such groups [cultivation of plants, domestication of animals, and land management (14–16)]. Typologies hide the myriad of subsistence practices which occur. Moreover, within subsistence types, there is a diverse range of expression of related traits, such as residential mobility, political structures, wealth and resource access, inheritance, and marriage patterns, all of which have implications for fertility (2, 3, 17–19). Such nuance is lost with reliance on subsistence typologies. Unsurprisingly then, population-level approaches may struggle to identify clear fertility differentials between subsistence strategies (5, 7, 8). Such difficulties are only increased by market integration.

## The Issue of Market Integration

Almost all contemporary “subsistence” populations, in which individuals still produce their own food, engage to some degree in the monetary economy. Market integration has long been associated with declining fertility in a wide range of contexts (20–22), driven by individual- and group-level mechanisms. Formal education—required in a skills-focused labor market—is frequently negatively correlated with fertility (23). This has been attributed to several factors, including delays in the age of marriage and first birth (24), increasing the costs of raising children resulting in parents investing more in fewer children (25, 26), and reduced mortality rates linked to increased wealth and knowledge (27, 28). Increased access to mass media and alternative sources of knowledge (e.g., of biomedical contraception) may result in a convergence on low fertility (29), potentially further promoted by less “kin-dense” social networks (21), as kin are predicted to be more pronatal and cooperative (30). Given the number of pathways through which market integration may be associated with fertility, and strong relationships between market integration and fertility in previous research, it needs to be incorporated into any study of subsistence and fertility in contemporary populations. Here, we do not treat market integration as a subsistence “type,” as it occurs in parallel with subsistence-level behaviors; individuals often complement existing strategies with the market in transitioning populations.

We curated a large (n = 10,250) cross-cultural database on women’s individual-level fertility and subsistence practices from 27 small-scale populations (*SI Appendix*, Fig. S1) to explore whether subsistence activities are associated with fertility. Using multilevel Bayesian models, we model individual fertility (computed as predicted cumulative fertility at age 60) to answer three questions. Question one asks: Within subsistence type and populations, how diverse are subsistence practices and fertility? Given our concerns with subsistence typologies, we expect little consistency in fertility and subsistence activities at the population level. Questions two and three ask, at the individual level: Are traits associated with farming predictive of higher cumulative fertility, and are traits associated with foraging predictive of lower cumulative fertility? And do measures of market integration predict lower cumulative fertility? Based on previous results and potential mechanistic pathways (10–12, 21, 31), we predict that individual measures of investment in farming will positively predict cumulative fertility while the reverse will be true of foraging. We expect market integration to be broadly predictive of lower cumulative fertility, but following (32), associations may differ according to which measures of market integration are used.

## Results

### Research Question 1: How Diverse Are Subsistence Practices and Fertility within Populations?

**Fig. 1.**
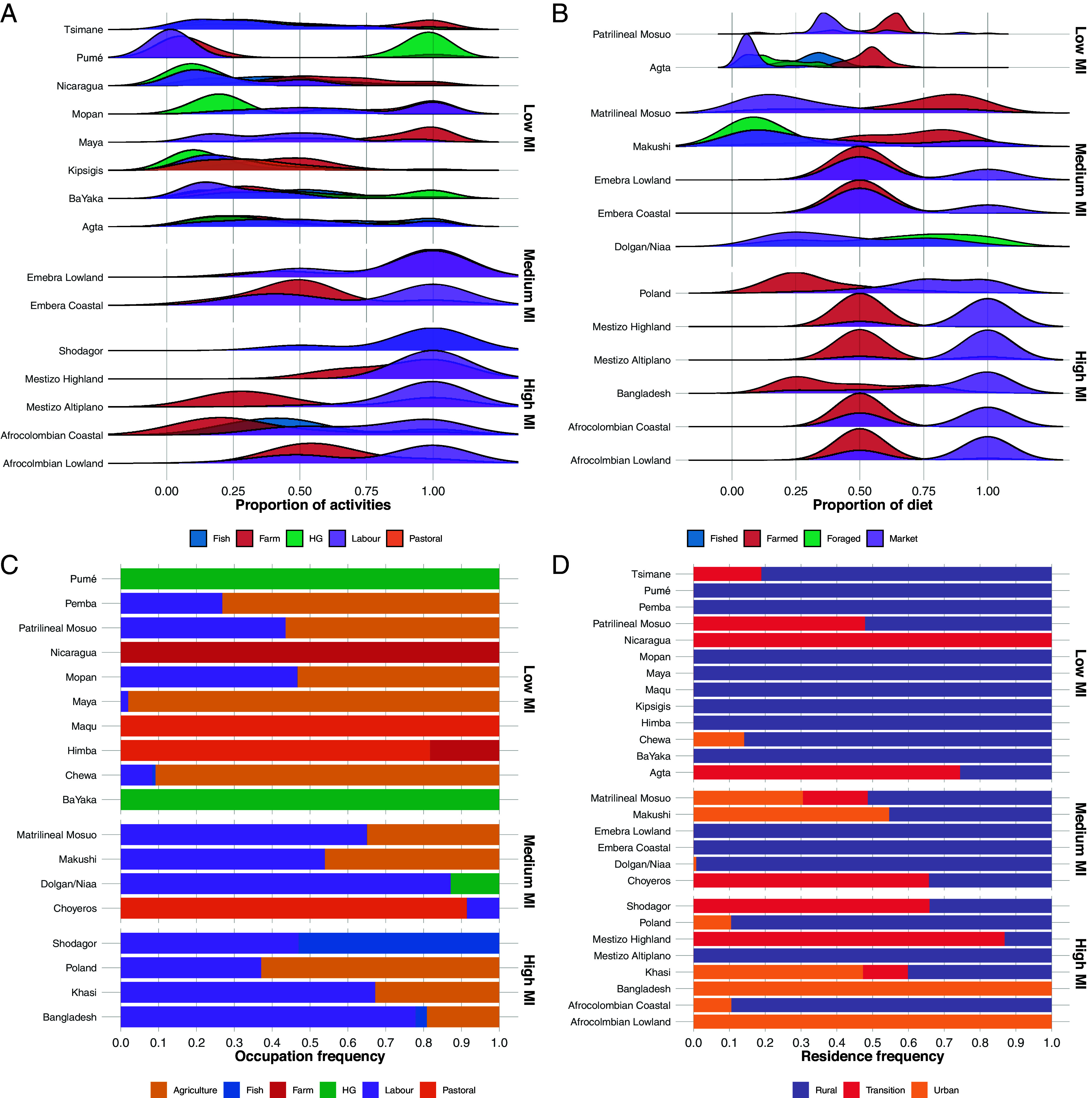
Descriptive statistics for four key subsistence metrics—(*A*) proportion of activities in different modes of subsistence, (*B*) proportion of diet from different sources, (*C*) self-reported occupation, and (*D*) type of residence. Plots are at the population level (*Y* axis), but not all subsistence measures are available for all populations, so those listed vary. Plots are structured by degree of market integration, running from *Top* to *Bottom*: “low,” “medium,” to “high.” The colors of the curves/bars reflect the investment in the subsistence strategy. Note that in plots (*A*) and (*B*), “farm” represents the combined influence of agricultural and horticultural production. The sample size varies by measure; please see *SI Appendix*, Table S1 for further descriptives.

Individuals took part in a wide range of economic activities not necessarily aligned with the subsistence type of their population (Fig. 1). Populations less engaged with the labor market reported more subsistence and dietary diversity. Some populations (e.g., Pumé), while engaging in multiple subsistence practices, noticeably preferred one, producing clear density peaks, suggesting predominant reliance on one mode of subsistence. In contrast, others (e.g., Agta and Tsimane) lacked distinct density peaks, suggesting no one subsistence mode dominated. The results for occupation and residence mimicked these patterns.

**Fig. 2.**
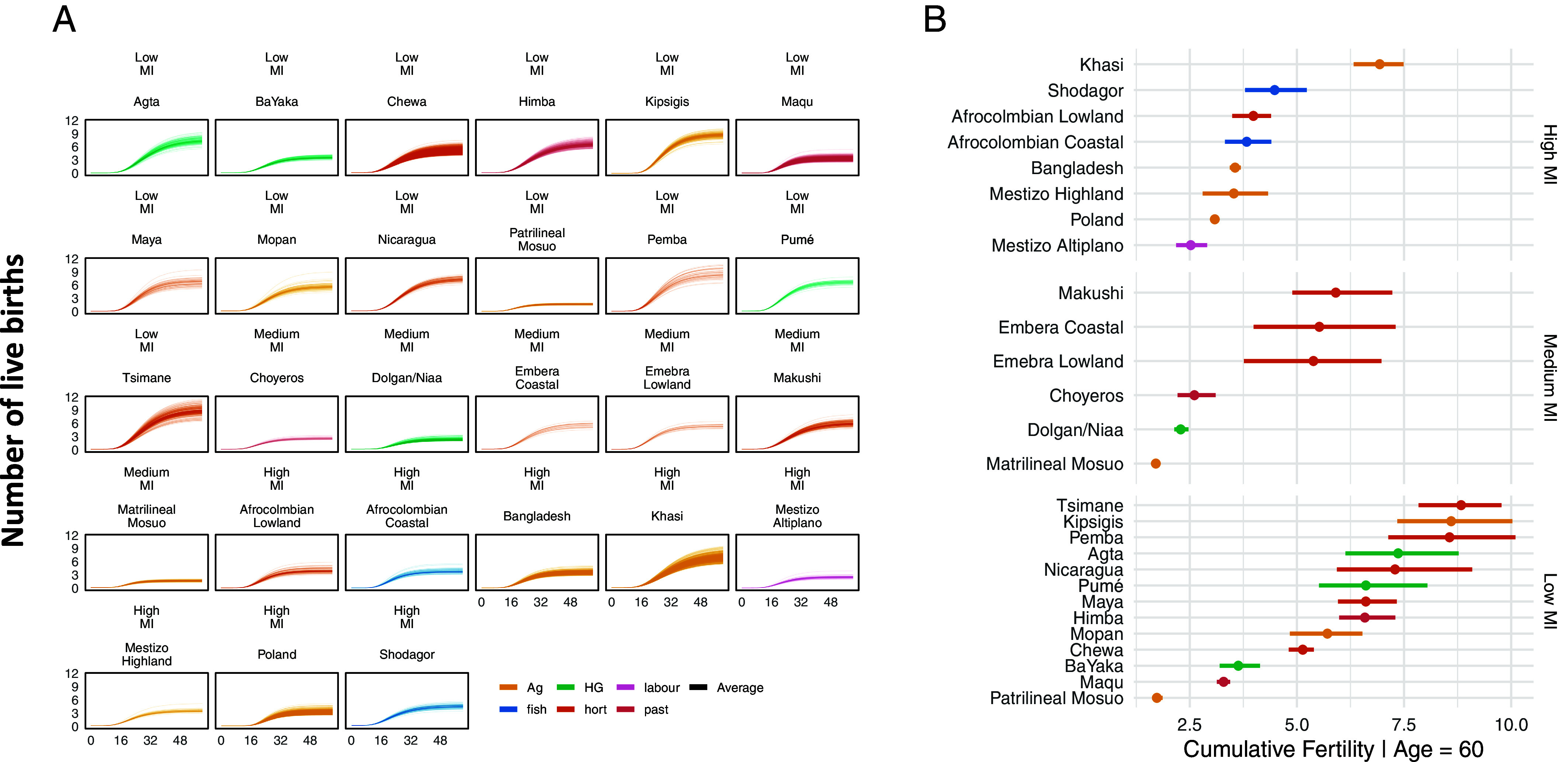
(*A*) Live births as a function of age in each study population. Within populations, each line is the posterior median age function for an individual woman. The dotted intervals denote 90% credible intervals of the posterior predictive distribution. (*B*) Points represent posterior median predicted cumulative fertility ($\tilde{\mathrm{CF}}$). Bars represent 90% CI. Vertical facets on the right denote level of market integration (low/medium/high). In both plots, the main subsistence type is color coded.

Fig. 2 reveals the large amount of individual-level variation in fertility within and between populations, though some populations have greater variation than others. This variation does not clearly map on to subsistence typology (*SI Appendix*, Fig. S3). Hunter–gatherers had a posterior median cumulative fertility of 4.6 (90% (highest posterior density intervals) HPDI [3.17, 6.05]), compared to horticulturalists’ posterior median of 5.61 (90% HPDI [2.99, 5.16]) and agriculturalists of 4.09 (90% HPDI [2.99, 5.16], *SI Appendix*, Table S3). The 90% credible intervals for all groups are large and overlapping (*SI Appendix*, Fig. S3*A*. In contrast, the difference in mean cumulative fertility was more substantial between high (posterior median= 3.53, 90% HPDI [2.62, 4.45]) and low (posterior median= 5.5, 90% HPDI 4.32, 6.81]) levels of market integration ($\tilde{\Delta \mathrm{CF}}$ = −1.96, 90% HPDI [−3.62, −0.34], posterior probability (PP) = 0.972, *SI Appendix*, Fig. S3*B*), though there was still considerable variation within each level of market integration.

### Research Question 2: At the Individual Level, Are Farming Activities Predictive of Higher, and Foraging Activities Predictive of Lower, Cumulative Fertility?

**Fig. 3.**
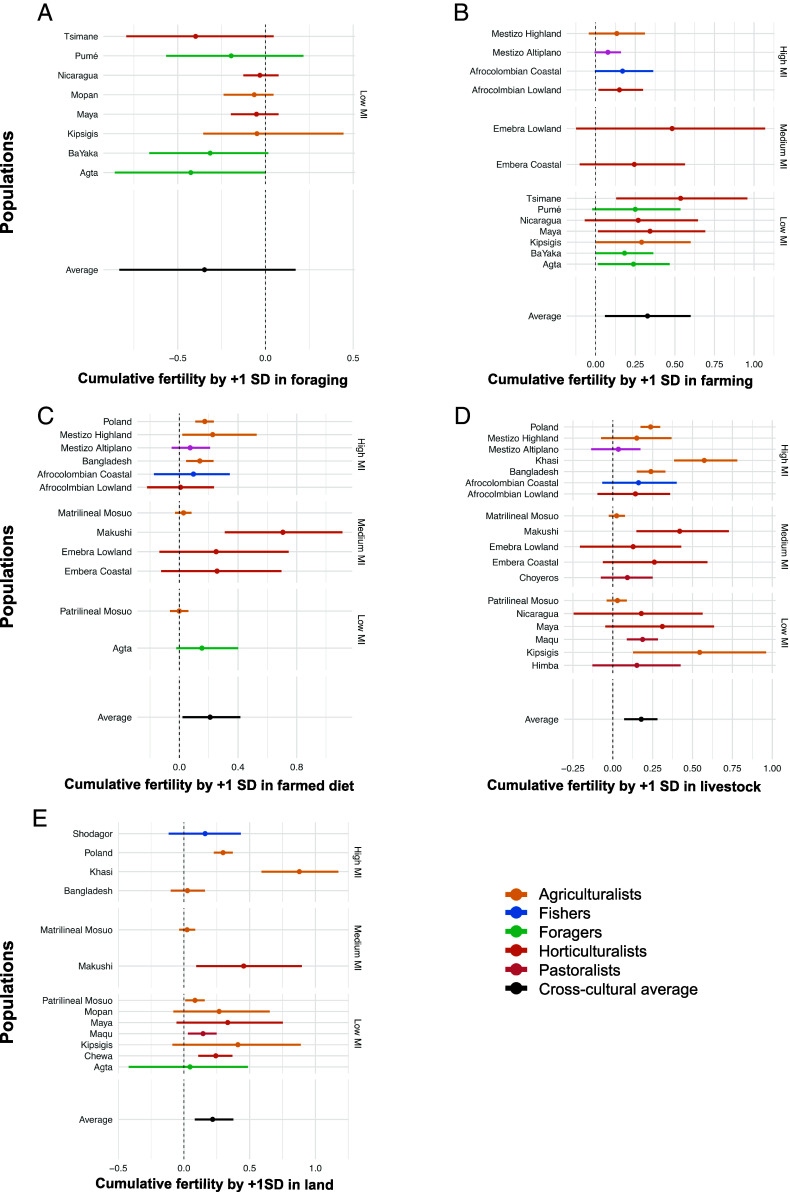
Relationship between five measures of individual subsistence strategy and mean cumulative fertility. (*A*) Foraging activities as a proportion of all economic activities (n = 8), (*B*) cultivation activities as a proportion of all economic activities (n = 13), (*C*) proportion of diet from farmed sources (n = 12), (*D*) log count of livestock (n = 18), and (*E*) log (acres) of land owned (n = 13). Points represent posterior median expected cumulative family size ($\tilde{\mathrm{CFS}}$). Bars represent 90% CI. Vertical facets on the right denote level of market integration (low/medium/high) in all plots except *A*, where all populations are categorized as “low” market integration. The main subsistence type is color coded. Effect sizes in plots (1+SD) are relative to each individual population.

**Fig. 4.**
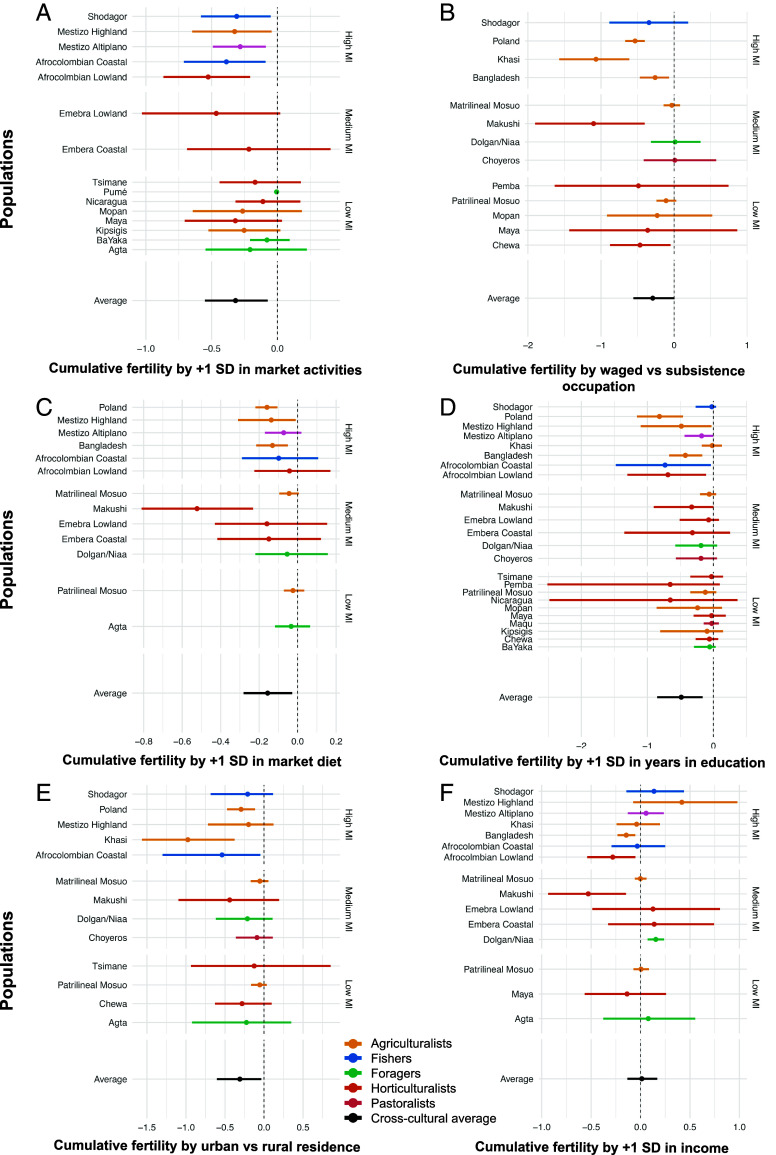
Relationship between six measures of market integration and mean cumulative fertility. (*A*) Wage labor activities as a proportion of all economic activities (n = 15), (*B*) waged versus subsistence occupation (n = 13), (*C*) proportion of diet from market sources (n = 13), (*D*) log years in education (n = 24), (*E*) urban versus rural residence (n = 13), and (*F*) log income (n = 15). Points represent posterior median expected cumulative family size. Bars represent 90% CI. Vertical facets on the right denote level of market integration (low/medium/high) in all plots. The main subsistence type is color coded. Effect sizes in plots (1+SD) are relative to each individual population.

Analysis at the individual level shows that increased involvement in foraging (Fig. 3*A*) was moderately and negatively correlated with reduced fertility. Across the entire dataset A 1 SD increase in the proportion of economic activities dedicated to foraging was associated with a 0.35 posterior median reduction in cumulative fertility (90% HPDI [−0.87, 0.13], PP = 0.89). When considering these associations for each population separately, they were strong for populations designated as hunter–gatherers (*SI Appendix*, Table S5) and moderate to weak for horticulturalists and agriculturalists. Measures of farming were strongly associated with higher cumulative fertility (Fig. 3 *B*–*E*): A 1-SD increase in the proportion of farming activities was associated with a 0.33 posterior median increase in mean cumulative fertility (90% HPDI [0.05, 0.60], PP = 0.981, Fig. 3*B*), and a 1-SD increase in the proportion of the diet coming from farmed foods was strongly associated with a 0.21 posterior median increase in cumulative fertility (90% HPDI [0.02, 0.42], PP = 0.984, Fig. 3*C*). However, these results were weak for a number of populations defined as agriculturalists and stronger for foragers and horticulturalists (*SI Appendix*, Table S7). Similar strong trends were apparent for 1 SD increases in log count of livestock ($\tilde{\Delta \mathrm{CF}}$ = 0.18, 90% HPDI [0.07, 0.29], PP = 0.997, Fig. 3*D*) and land ($\tilde{\Delta \mathrm{CF}}$ = 0.22, 90% HPDI [0.08, 0.37], PP = 0.995, Fig. 4*E* and *SI Appendix*, Tables S16 and S17 include controls for wealth), alongside weak and moderate associations in some populations. Collectively, Fig. 3 provides strong evidence that farming is positively associated, while foraging is moderately associated, with cumulative fertility cross-culturally. Yet, these results do not hold in all populations, suggesting context-specific variation in the relationship between fertility and subsistence.

### Research Question 3: Do Measures of Market Integration Predict Lower Individual-Level Cumulative Fertility?

Increased integration in the market was strongly associated with lower posterior median cumulative fertility for most measures (Fig. 4 and *SI Appendix*, Tables S10–S15). A 1 SD increase in the proportion of economic activities dedicated to wage labor was strongly associated with a −0.32 reduction in posterior median cumulative fertility (90% HPDI [−0.56, −0.07], PP = 0.976). These results were more consistent for populations with greater market integration and those engaged in more wage labor (Fig. 4*A*). Similar results are apparent for the proportion of the diet from market sources ($\tilde{\Delta \mathrm{CF}}$ = −0.16, 90% HPDI [−0.28, −0.03], PP = 0.968, Fig. 4*C*). Individuals who reported a salaried occupation were strongly associated with a lower posterior median cumulative fertility compared to those who reported a subsistence-based occupation ($\tilde{\Delta \mathrm{CF}}$ = −0.29, 90% HPDI, [−0.55, 0.01]. PP = 0.953, Fig. 4*B*), matching the pattern associated with urban versus rural residence ($\tilde{\Delta \mathrm{CF}}$ = −0.34, 90% HPDI [−0.66, −0.06], PP = 0.979, Fig. 4*E*). Again, for both salaried occupation and urban residence, the associations appear strongest in those most integrated into the market, while the inverse was true for those least market integrated. The most consistent predictor of fertility was education: A 1-SD increase in years spent in education was associated with a −0.47 drop in posterior median cumulative fertility (90% HPDI [−0.83, −0.15]. PP > 0.999, Fig. 4*D*). In contrast, the results associated with income were the weakest ($\tilde{\Delta \mathrm{CF}}$ = 0.01, 90% HPDI [−0.13, 0.16], PP = 0.554, Fig. 4*F*) and not consistent across populations, negative in some (e.g., Makushi and Bangladesh) and positive in others (e.g., Agta and Dolgan, *SI Appendix*, Table S15), producing an average weak association between income and cumulative fertility.

## Discussion

Our results suggest that classic subsistence typologies predict fertility poorly since subsistence activities within typologies are varied. Cross-culturally, at the individual level, which provides a cleaner test of our hypothesis, we find moderate evidence that individuals who engage more in foraging have lower fertility and strong evidence that those who engage more in farming have higher fertility. Those who engage more in market activities have lower fertility. While the point estimates across models are consistent in their direction (positive for farming measures; negative for foraging and market integration), when we look at each population separately, the results are noisier. Explicitly, the point estimates and 90% credible intervals hover by, or straddle 0, indicating little effect in some populations, dependent on the outcome. This highlights the considerable variation present in the relationship between fertility and subsistence. In other words, context matters. Inconsistencies in prior research may therefore have reflected the lack of predictive power of subsistence typology at the population level (8, 33). Subsistence, at the individual level, may have implications for fertility, but subsistence alone is not enough to predict fertility.

Our descriptive results highlight that most populations engage in substantial subsistence diversification. This is most evident in the least market-facing populations who engage in three-to-four subsistence strategies. This is in line with both the archaeological and ethnographic record which suggests that daily subsistence activities rarely conform to a discrete typology (14–16, 31). Mixed subsistence strategies may therefore have a deep history in our species. This may explain why subsistence typologies are poor predictors of fertility. Furthermore, beyond direct economic activities, populations also vary in their expression of social and political structures, degrees of sedentism, food storage, and wealth accumulation (34, 35), each of which are independently associated with fertility [wealth (17, 22), social status (36), mobility (12)]. Such traits often, but not necessarily, cluster with subsistence. Given this variability, subsistence typologies and individual behaviors do not necessarily overlap, reducing the explanatory power of any subsistence typology. Our results here show that we should be wary of assuming that subsistence typology is a useful proxy for individual-level behaviors.

In our analysis of fertility, we were only able to use one measure of foraging (proportion of activities spent in hunting and gathering) and found only moderate evidence across the eight populations (who engaged in some foraging) of a negative association with fertility. This association is strongest in populations defined as foragers or horticulturalist-foragers, suggesting that low levels of foraging in more intensive farming contexts may not have sufficient influence to affect fertility. Alternatively, it may be groups engaged in foraging experience the largest fertility gains from small increments of resource extraction intensification. Measures associated with farming (activities and dietary sources, land ownership, and livestock) were strongly associated with higher cumulative fertility. Land and livestock ownership are also metrics of wealth and social status (17); key predictors of fertility in many settings (17, 22). To confirm that wealth is not confounding this relationship, we included material wealth as a control (please see *SI Appendix*, Figs. S4 and S5). This inclusion had no effect on our results, highlighting our variables of land and livestock are capturing variance beyond that of wealth. Our predictor variables are modeled independently for this reason, as they capture different dimensions of varying investment in subsistence strategies: some being longer-term, stable, measures (like land or occupation) and wage labor and others (like diet and daily activities) capturing short-term measures of subsistence activity. Each has strengths and weaknesses, which is why we consider and interpret the trends across multiple models collectively to capture different dimensions of subsistence.

Market integration and subsistence were modeled separately because it is impossible to unpack the relationship between the two, as some modes of subsistence, such as intensive agriculture, are necessarily associated with increased market engagement: Our population-level results hint at this, as they suggest that agriculturalists may have lower fertility than horticulturalists (*SI Appendix*, Fig. S3). Modeling subsistence and market integration separately is then a conservative approach since the associations are in opposing directions (the market negatively predicts fertility, farming positively), making it more difficult to find real associations (if they exist).

Measures of market integration were strongly associated with lower fertility. The exception was income, likely because it measures both increased involvement in wage labor (typically a negative predictor of fertility in market integrated populations) and wealth (a positive predictor in subsistence-level populations). Overall, there is essentially no relationship between income and fertility; when individual populations are considered, the direction and strength of the relationship is not consistent across populations or degree of market integration. Across all the market integration results, in populations with no or little market integration (especially the populations defined as foragers), the associations were close to null, compared to the most integrated populations where the effects were strong, clearly diverging from the null. This may suggest a threshold or a “tipping point” effect. At low levels, the exposure to the market may not be consistent, or powerful, enough to alter long-standing behaviors, cultural norms, and cooperative dynamics.

We have taken an agnostic approach to the mechanisms underpinning fertility regulation. Such an approach is common within human behavioral ecology (37) and demography (38), allowing the full description of overarching relationships. A number of potential mechanisms have been proposed, through which subsistence may be associated with fertility (19). For instance, farming is associated with relatively low mobility, meaning women may experience higher energy availability allowing greater investment in fertility (39, 40). Subsistence activities also affect women’s and children’s work patterns (16), which may both influence energetic availability and the role of children in childrearing (2, 11). Such energetic differences are a common explanation for potential differences in fertility between subsistence strategies. A recent analysis by (9) calculated the subsistence cost and energetic acquisition of a hunter–gatherer (Hadza) and forager-horticultural population (Tsimane), finding that horticulture was associated with improved efficiency compared to hunting and gathering, particularly for women. Horticulture, therefore, is associated with improved return rates, increasing energetic availability to women which arguably can produce shorter interbirth intervals. Future work should explore which specific aspect of fertility (interbirth interval versus age at first or last birth) are predicted by subsistence to provide support for women’s energetics as a proximate mechanism. Yet, other proximate pathways—such as changes in reproductive and marriage norms (33) and alterations in individuals’ social networks (19, 21, 30)—impact fertility, and may do so through both birth intervals and reproductive timing. Furthermore, group-level dynamics, such as the promotion of lower fertility norms in individuals’ social networks (21), influence ideal family sizes and stopping behaviors. Notably, group-level market integration was a stronger predictor (as compared to subsistence type) of fertility, suggesting that the mechanisms driving fertility operate simultaneously at both the individual and group levels (25, 26, 29). To date, there has been little systematic research to test and separate out these diverse pathways, an important focus for future research.

One reason for the widely held belief that farming is associated with higher fertility is that a number of pieces of research explicitly or implicitly suggest that fertility was the driving factor for the rapid population growth associated with the Neolithic Demographic Transition (40–43). Due to necessary limitations of the archaeological record, scholars of prehistoric demography frequently rely on data from contemporary populations to parameterize their models (44). In addition to the challenges of such data transportation deriving from systematic differences between past and present populations, our results perhaps caution against relying solely on the foraging/farmer dichotomy as a causal framework for assuming differential fertility between populations and for understanding the coevolution of human subsistence and fertility more broadly. Developing a deeper understanding of the mechanisms which link subsistence to fertility, and how behaviors vary by socioecological context (even within the same subsistence type), may help the support interpretations of existing archaeological data.

A key limitation is the use of secondary data from existing anthropological sources which have not been collected using the same protocols, making comparisons less than perfect. The second limitation is the cross-sectional nature of the data and the difference in the temporal resolution in the subsistence versus the reproductive data. Subsistence behaviors gathered at one specific time point do not necessarily represent life-long subsistence. While we expect this issue to be more problematic with time budget data, this was a key reason for the use of many different components of subsistence which collectively are less time-varying. Longitudinal and large-scale, comparative projects using equivalent protocols, are becoming more common in the behavioral sciences, suggesting future work can overcome these issues. Another important influence on the accuracy of reproductive histories is the impact of selection and recall bias: Women who survive to report their reproductive histories are a subset of all those born, and as individuals age, they are increasingly likely to miss live births during interviews, artificially reducing their family sizes. By basing our analysis on all reproductive-aged women in the population, we have minimized the bias associated by using only cumulative fertility. Finally, while our sample of foragers is relatively small, this represents contemporary reality. By including individual-level analysis, we have maximized the foraging sample available, by including any woman who engages in foraging, even if not in a “foraging” society.

## Conclusions

There is no evidence—at the population level—that farmers outreproduce other subsistence types, but there is strong evidence across cultures that—at the individual level—fertility is positively associated with farming. While the evidence is weaker, individuals who engage in more foraging activities have lower cumulative fertility. Indicators of market integration have strong, negative relationships with fertility. Nonetheless, these relationships are not consistent in all populations studied. Contra common assumptions, we cannot predict an individual’s fertility based on their subsistence alone, likely because of the influence of socioecological context. Context may both mean that subsistence activities themselves are somewhat different in different ecologies, even among those who adopt the same subsistence strategy, and affect other biological and sociocultural mechanisms which influence fertility.

## Materials and Methods

This project was approved by the LSHTM Research Ethics Committee (25072). As this analysis is based on secondary data analysis all methods were performed in accordance with the original institutional and local ethical approvals and any relevant regulations (see *SI Appendix* for further methodological information including how informed consent was acquired within each population).

Fertility was established from reproductive histories with women who had reached reproductive maturity (aged 14 and above). We computed a summarized variable for the cumulative number of live births for each woman. At the population level, subsistence typologies were provided by each ethnographer (either hunter–gatherer, pastoralist, horticulturalist, agriculturalist or fisher), and each was asked to provide an estimate of whether market integration in the population they worked with as either “low,” “medium,” or “high.” We do not use the ethnographer coded in research questions 2 and 3 given the above discussion about the importance of individual-level data and the lack of clear boundaries between, for instance, hunter–gatherer or horticulturalist populations. We do, however, use these structures in the figures to scaffold our results.

At the individual level, several different measures of subsistence strategy and market integration were collected, though each was only available for a subset of populations. Observational approaches (scan sampling) quantified the proportion of activities individuals devoted to different subsistence activities (fishing, foraging, farming, pastoralism, and wage labor) and were collected by 15 study sites. Here, when we refer to farming, unless otherwise stated, we include all types of cultivation (as distinctions between agriculture and horticulture were not always captured, or possible, when collecting some types of individual-level data). Foraging includes all natural environment food acquisition, incorporating fishing, gathering, and hunting. Researchers from 13 study sites asked participants about the sources of food consumed. We summed the number of instances of food consumed from each source, dividing by the total consumed to create three variables: proportion of diet from either farming (anything grown, including horticulture and agriculture), the market (anything purchased), or foraged (acquired from the natural environment). Household interviews produced data on primary occupations (n = 18, categories included intensive agriculture, horticulture, foraging, fishing, pastoralism, and wage labor), amount of land (acres, n = 13), total livestock units (n = 18), annual reported income (n = 15), and educational attainment (years, n = 24). All collaborators who contributed data defined household locations for each of the research sites as rural, transition, and urban.

We use population-level descriptors such as subsistence typology and degree of market integration as heuristic devices to scaffold our understanding of the results. Our use in the discussion of subsistence types or low, medium, and high market integration is not to suggest these are fixed, bounded entities.

### Analysis.

#### Causal assumptions.

**Fig. 5.**
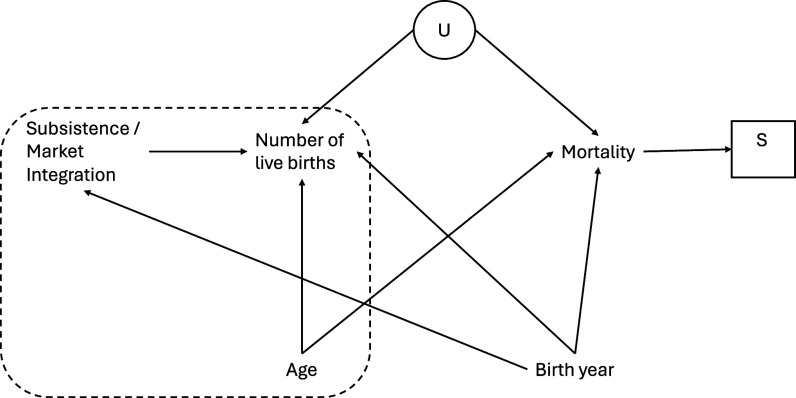
DAG representing our assumptions about the data-generating process. The dashed box contains our main variables, with threats to identification [birth year, selection (S) due to missing records of deceased individuals] shown outside the box alongside of unobserved confounding (U).

The causal assumptions underpinning our models are presented in a directed acyclic graph (Fig. 5, further detail provided in *SI Appendix*). Our predictors are clustered under the exposure subsistence/market integration, the causes of cumulative live births. We purposefully ran our two exposure variables in separate models, not including terms for market integration within the subsistence models and vice versa. We significantly increase our causal and variable independence assumptions when adjusting for market integration since it is not possible to robustly separate the effects of subsistence and market integration. We recognize that we cannot completely isolate the casual roles of subsistence, market integration, and fertility because of the nature of anthropological data. Noncausal paths remain open in our DAG (Directed Acyclic Graph) due to unavoidable unmeasured variables. For this reason, we have sought to keep our assumptions to a minimal level, keeping models simple. Yet, we also believe this to be a pragmatic and useful approach to this analysis to clarify how we expect and thus interpret these relationships.

Our models, based on these causal assumptions are exploring the overarching relationship between fertility and subsistence and as a result do not include other, proximate, variables which exist on the causal pathway. Two such examples include infant mortality and contraception, both factors through which subsistence and market integration may impact fertility. As a result, it is not appropriate to include variables on the causal pathway in the models. Biomedical contraception is used to varying degrees in the populations included in this analysis and on average has a weak to moderate positive relationship with fertility across the sample (posterior median= 0.21, 90% HPDI [−0.24, 0.7], PP = 0.798), in line with previous findings (45). Readers are directed to supplementary analysis for further information.

In recognition that two of our farming measures—livestock and land ownership—may also be (population-dependent) measures of wealth, we ran a supplementary analysis presented in *SI Appendix* controlling for material wealth to ensure that these models were not simply picking up an association between wealth and fertility. Given not all study sites had provided data on all measures (e.g., many farmers conducted no foraging), the sample varied by model.

#### Statistical modeling.

We modeled cumulative live births as a one-inflated Poisson process with rate parameter λ and one-inflation parameter θ. λ is modeled as a growth function derived from (46) and recently used to model other aspects of human life history such as learning and foraging skill by (47, 48). We estimate posterior median differences in cumulative fertility between women within populations as a function of different subsistence and market integration predictors, estimates which were pooled across population in the multilevel model. We define the predicted cumulative fertility as $E\left[CF\right]=E\left[\text{Live}\;\text{Births}\;|\;\text{Age}=60\right]$. In the results, we denote the posterior median of predicted cumulative fertility as $\tilde{\mathrm{CF}}$, and differences as $\tilde{\Delta \mathrm{CF}}$. We report 90% HPDI of posterior distributions from multivariate models. Continuous predictors are z-scores within population, comparing relative variation within populations. We interpret associations between predictions and outcomes as strong when 90% of posterior distributions do not include 0 (e.g., PP equal to, or higher than 0.9), moderate when 80% of posterior distributions do not include 0, and weak when less than 80% do not include 0. All analyses were run in R using the RStan package, which fits Bayesian mixed-effect models (accounting for population random effects) using Hamiltonian Markov chain Monte Carlo (MCMC), assessed using standard diagnostics (number of effective samples, the Gelman–Rubin diagnostic, and visual inspection of trace plots). Sensitivity analyses (wealth and biomedical contraceptive effects) and posterior model checks are presented in *SI Appendix*.

## Supplementary Material

Appendix 01 (PDF)

## Data Availability

Anonymized CSV file data have been deposited in OSF (https://osf.io/8d9n2/?view_only=9e07c25​e06414f7a8​d041e80e8539e5c) (49).
