# Supplementary material for: Women’s subsistence strategies predict fertility across cultures, but context matters
Source: Proc Natl Acad Sci U S A. 2024 Feb 12;121(9):e2318181121. doi: 10.1073/pnas.2318181121 (PMC10907265; doi:10.1073/pnas.2318181121)
Supplement: Supplementary file 1 — Appendix 01 (PDF) [file pnas.2318181121.sapp.pdf]

SI: Women's subsistence strategies predict fertility across cultures, but context matters

Table of Contents

Study site ethnographic and methodological information ***4***

Agta, Philippines **4**
Ethnographic information 4
Data collection 4
Ethics 6

BaYaka, Likouala Department, Republic of the Congo **6**
Ethnographic information 6
Data Collection 7
Ethics 8

Chewa, Malawi **9**
Ethnographic information 9
Data Collection 10
Ethics 11

Rural Afrocolombian, Emberá, and Mestizo Communities; Colombia **11**
Ethnographic information 11
Data collection 12
Ethics 13

Dolgan and Nganasan, Russian Federation **13**
Ethnographic information 13
Data collection 15
Ethics 16

Himba, Namibia **16**
Ethnographic information 16
Data collection 17
Ethics 18

Khasi, India **18**
Ethnographic information 18
Data collection 19
Ethics 20

Kipsigis, Kenya **20**
Ethnographic information 20
Data collection 21
Ethics 21

Makushi, Guyana **22**
Ethnographic information 22
Data Collection 22
Ethics 23

Maya, Mexico **23**
Ethnographic information 23
Data collection 24
Ethics 25

Matlab, Bangladesh **25**
Ethnographic information 25
Data collection 27
Ethics 28

Maqu Tibetans, China **28**
Ethnographic detail 28
Data collection 28
Ethics 29

Mbendjele BaYaka, Republic of the Congo **29**
Ethnographic Information 29
Data collection 29
Ethics 31

Mopan, Belize **31**
Ethnographic information 31
Data collection 32
Ethics 33

Mosuo (matrilineal and patrilineal), China **33**
Ethnographic information 33
Data collection 34
Ethics 34

Mayangna, Nicaragua **35**
Ethnographic detail 35
Data collection 35
Ethics 36

Pemba, Zanzibar **36**
Ethnographic information 36
Data collection 37
Ethics 38

Rural Poland **38**
Ethnographic information 38
Data collection 39
Ethics 40

Pumé, Venezuela **40**
Ethnographic information 40
Data collection 41
Ethics 42

Shodagor, Bangladesh **42**
Ethnographic information 42
Data collection 43
Ethics 44

Rancheros: Baja California Sur, Mexico **44**
Ethnographic information 44
Data Collection 45
Ethics 45

Tsimane, Bolivia **46**

Ethnographic information 46
Data collection 48
Ethics 50

***Extended methods* 50**
Figure S1 50

Activity budgets 50

Dietary sources 51

Occupation 51

Land, livestock, income and education 51

Ethnographer reported information 52

Live births and ages at first and last birth variables 52

Modelling 53

DAG 54
Figure S2 55

***Extended results* 56**
Figure S3 56
Table S1 57
Table S2 61
Table S5 62
Table S6 63
Table S7 64
Table S8 65
Table S9 66
Table S10 67
Table S11 68
Table S12 69
Table S13 70
Table S14 71
Table S15 72

***Sensitivity Analysis* 73**
Table S16 73
Figure S4: 74
Table S17 75
Figure S5 76
Table S18 77
Figure S6 78

Model checks 79
Figure S7 79
Figure S8 80
Figure S9 81

***SI References* 82**

Study site ethnographic and methodological information

Agta, Philippines

Ethnographic information

There are around 1,000 Agta living in the Palanan municipality of north-eastern Luzon, Philippines. Riverine and marine spearfishing provides their primary source of animal protein (54% of economic activities were fishing), supplemented by hunting (3% of economic activities) and gathering (20% of activities), as well as low-intensity cultivation (12% of activities), wage labour (9.5%) and trade (2.1% of activities) (1, 2). The resources on which they subsist are situated in the Northern Sierra Madre Natural Park (NSMNP), the Philippines' largest protected area (1) that consists of a mountainous tropical rainforest and includes the coastal beaches, coral reefs and the marine eco-system of the Pacific Ocean. There is significant variability within the population in terms of mode of subsistence, type of residence and market integration (2–4). The Agta follow a bilateral descent and residence system, which maintains a large and flexible kinship network facilitated by residential mobility (1, 5–8). Previous research has highlighted their extensive cooperation, between kin and non-kin, in the domains of food sharing and childcare (5, 8–11). The Agta practice serial monogamy and have a total fertility rate of 7.7 and a short average interbirth interval of 2.8 years. Infant and childhood mortality rates are high, with an estimated 38.9% of offspring dying before the age of 16 years (3).

Data collection

Data collection occurred over two field seasons from April-June 2013 and February-October 2014, conducted by Abigail E. Page, Daniel Major-Smith and Mark Dyble. In the first season we censused 915 Agta individuals (54.7% of which were male) across 20 camps. During this data collection period we conducted reproductive histories with all reproductively aged individuals. Accurate ages were established for all individuals post data collection since we rarely encountered individuals who knew their own age. To estimate age, we took the mean values from probability distributions of age produced using a Gibbs sampling MCMC algorithm based on age ranking order data and age ranges for each individual (see (12)). In the second season we stayed approximately 10-14 days in ten camps to conduct in-depth data collection including camp scans, interviews and food dairies.

Time budget data were collected by conducting four observational scans each day at staggered intervals during daylight hours. We conducted the first scan between 0630 and 0930 and then recorded three more scans at three-hour intervals. In each scan, we recorded the activity that every member of camp was engaged in (categorised into childcare, domestic chores, leisure and out-of-camp work) and, entering data on individuals as we encountered them. Agta camps are typically concentrated in a small area and the activities of most individuals are visible from a central place. When individuals were out of camp, we asked those in camp what the absent individuals were doing and verified this when the individual returned. In some cases, one of the authors was present with individuals on out-of-camp work. Time spent by individuals in other camps was excluded from our data and, similarly, we excluded visitors to our study camp, including only individuals who spent four days or more in the study camp. Our time allocation categories were modified from those developed for the Agta in a previous study (13). This produced time allocation data of 359 people across ten camps (including 71 adult men and 71 adult women, >18 years) which resulted in a total of 10,706 person-observations. See (2) for further methodological information.

We conducted household interviews to quantify demographics and household features with the mother of the household. Each mother was asked to document each child, in order of their births, and their rough dates of births. If mother's did not know the exact date of birth this was triangulated with known events based on the relative aging method (12). We would always specifically enquire whether a mother had experienced any miscarriages or stillbirths. During these interviews we also enquired how much land and income a household had, as well as the number of years each household member attended school. Interviews were conducted by a field assistant who spoke the local language and an Anthropologist who did not. The field assistant translated the content of the interview. No issues of understanding arose as the anthropologists quickly learnt the language required to quality check interviews over the course of data collection.

Dietary data were collected at the household level at the end of each day by Mark Dyble who asked the mother and the father at the end of the day (between 17:00 – 18:00) what foods

they had eaten that day. These food types were then allocated to a foraged, farmed or market source. No information on amount of food or calorific content was collected.

Ethics

Funding for the data collection was kindly provided by the Leverhulme Trust (awarded to Andrea Migliano), in addition to the UCL graduate fund which further supported this work (awarded to Abigail E. Page). Our thanks go to all the Agta families who took part in the research, in particular for their patience and acceptance. Further thanks are due to our research assistances Ata Christie, Gurly and Amay Crumpez and Imay Crumpez. Ethical approval was provided by the UCL Ethics Committee (UCL Ethics code 3086/003) and carried out with permission from local government and tribal leaders in Palanan. Informed consent was obtained from all participants, and parents signed the informed consents for their children (after group and individual consultation and explanation of the research objectives in the Agta language.

BaYaka, Likouala Department, Republic of the Congo

Ethnographic information

BaYaka who participated in the present study live along the Motaba river of the Likouala department of the Republic of the Congo (14). BaYaka speak Yaka (di.Aka), and many also speak the language of their farmer neighbors and Lingala, the regional trade language (14). Like other hunter-gatherers, BaYaka maintain egalitarian social relations, food is shared widely, and personal autonomy is highly valued (14–18). BaYaka spend about six months of the year in forest camps averaging approximately 22 inhabitants (19), and the remaining year in a multi-ethnic village. In both settings, BaYaka participate in a variety of foraging activities, including fishing, hunting with spears, trapping, collecting tubers, fruit, leaves, mushrooms, nuts, seeds, and caterpillars (20). They also maintain low-maintenance horticultural gardens where they grow cassava, bananas, and other domesticated plants. While in the village, BaYaka work for their Bandongo farmer neighbours, with whom they maintain extensive trade relations regulated by fictive kinship (21–23). There are two primary schools in the village, which BaYaka children attend sporadically (24). While BaYaka have minimal direct interaction with markets, the forest resources they specialize in collecting (ivory, rubber,

duiker skins, honey, pepper, bushmeat) have played an important role in local, regional, and global economies historically and into the present (25–28).

Data Collection

Data collection occurred over two field seasons. As part of the “Effect of Social Networks on Inequality: A Longitudinal Cross-Cultural Investigation” project (https://endowproject.github.io/), Sheina Lew-Levy collected reproductive histories, levels of schooling, and wealth valuations in a village neighborhood in June and July 2018. Reproductive histories were updated in 2020 by Adam H. Boyette. Scan samples were conducted by Boyette and Kiabiya Ntamboudila in March 2020 for a two-week period before fieldwork was cut short by the Corona Virus pandemic. Because BaYaka do not know their age in years, we conducted an age-ranking task, statistically estimated age using the methods outlined in Diekmann and colleagues. (12) , and adjusted for a parent-age minimum age difference of sixteen years. In total, 79 women (age range; 20-80, mean = 44 years) participated in the study.

Whenever possible, observations (i.e., scans) were made three times per day (mean = 2.1 per day) between the hours of 07:00 and 17:00 and were spread across morning (07:00-10:00), midday (11:00-14:00), and afternoon (15:00-17:00) such that sampling reflected activities throughout the day. A random number table was used to choose specific starting times during each part of the day. At these starting times, Boyette and the field assistant (during training observations) or the field assistant alone would tour the BaYaka community, visiting each "hamlet", or group of closely associated households, and record the location and activities of each individual observed using a coding system. Location was broadly coded as either in the forest, at a residential camp in the forest, on the river, in the Bandongo farmer neighborhood of the village or at a garden. For activity, 63 different activity-codes were used to define the primary activity the individual was engaged in upon being observer. As childcare is often a non-mutually exclusive category in this setting (e.g., preparing food can be performed while holding a child in the lap), a sub-set of 21 codes for childcare activities were also coded simultaneously whenever observed. For each resident of the hamlet that was absent during the scan observation, another individual present was asked about their location and what they were doing. Whenever possible, individuals who were absent during a scan were asked

to confirm their location and activity during the subsequent visit. Over the two-week period, 2192 observations were made of 104 people (min. observations per person = 1, max = 41; mean = 21.04).

All individuals of reproductive age were invited to participate in an interview. Each participant was asked to list all the children they had given birth to and cross-referenced this list with that of their spouse if available. Where possible, we estimated child age, either by drawing from the age-ranking task described above, or by asking the participant to name someone of roughly the equivalent age of their child. Each participant was also asked how many school grades they had completed (e.g., if individuals spent more than one year to complete a Grade 1 this would still be recorded as '1').

We visited each household and counted all possessions (purchased or handmade) within them. We also asked present household members to list any possessions currently in use by an absent household member. We valued each item in XFA with the help of the village shopkeeper, which was then converted to USD.

Interviews were conducted by Lew-Levy who is conversational in BaYaka, and a BaYaka translator. Scan samples were conducted in Lingala, the local trade language, which is understood by all BaYaka participants.

Ethics

In-country permission to conduct the research was granted by the Institute de Recherche en Sciences Exactes et Naturelles (IRSEN). We obtained village-wide consent during a community meeting with BaYaka and Bandongo at the start of each field season. We also obtained individual verbal consent before data collection began. For data collection in 2018, ethical approval was obtained from the Cambridge Psychology Research Ethics Committee (PRE.2018.023). In 2020, data collection was conducted in accordance with the ethical guidelines of the Max Planck Institute.

Chewa, Malawi

Ethnographic information

Data were collected from two lakeside villages in the Southern region of Malawi in 1997 during a single round demographic survey, after previous pilot wok in the area in 1996. Both villages were originally settled by the matrilineal Chewa, who are largely subsistence farmers, though some households are moving into the market economy by engaging in wage labour, small businesses and trade. One village has also recently attracted migrants from other ethnic groups, primarily Tonga and Tumbuka, who are patrilineal groups from the north of the country, who moved south to engage in commercial fishing. Residents of the first village were very largely Chewa; about one third of the residents of the second village were Chewa. See (Sear 2008, Gibson and Sear 2012) for details on previous analyses of these datasets.

The Chewa were traditionally matrilineal and matrilocal. Women usually remained in their natal villages at marriage and lived close to their mothers and matrilineal kin. In this sample, over 90% of women reported residing in their own mother's village after marriage. Most women married husbands from the same village, however, as about 60% reported that their husbands' mothers were also available in their own villages after marriage. Women were allocated land by the matriline when they married, and farmed this land along with their husband and children. In this survey, in about 80% of all households' gardens were owned by women; men owned land in the majority of the remaining 20%; in only a handful of households did both sexes own land. The majority of these gardens were reported to have been inherited matrilineally (95% of gardens owned by women). Men also reported that the majority of their land had been inherited from matri-kin, but were more likely than women to report having inherited it from patrilineal kin: 75% of gardens owned by men came from matrikin and 19% from patri-kin (most commonly from fathers – 7% – or paternal grandmothers – 7%). There was little evidence for the importance of the mother's brother, at least in terms of land inheritance, as only two women and no men in this sample reported inheriting land from their mother's brother.

There is some evidence in recent years that the Chewa are adopting more patrilineal customs, with men becoming more likely to own their own land and pass it onto sons. However, there is also a degree of flexibility in land transfers, which may be due in part to increasing land

scarcity in Malawi, so that individuals must obtain land through non-traditional means. In this population, households in which men owned land tended to be somewhat wealthier than those in which women owned land: gardens owned by men were significantly larger than those owned by women; households where men owned gardens were also more likely to farm a larger number of crop types (including cash crops such as tobacco or cotton), and to own livestock and fishing gear.

There was little access to contraception and limited medical care available in this rural area (though information on contraceptive use was not collected during interviews). So, both fertility and mortality are relatively high. For example, 12% of all children born within the 5 years preceding the survey were reported to have died (though this may be an under-estimate as the 1998 Malawian census estimated that 12.1% of all Malawian children died just in infancy (the first year of life) in the year preceding the census). Marriages were usually monogamous: polygyny is not prohibited but in practice is rather rare (2% of women interviewed reported having co-wives), and where it does occur co-wives typically live in different villages. The singulate mean age at marriage for women in this population was 20 years, for men 25 years.

Data Collection

All households in the villages were invited to take part, and there were relatively few refusals. Both women and men of reproductive-age were interviewed, and asked to provide information on the number of children born. Interviews were conducted by a team of field assistants, who spoke the local language, and who translated the questionnaires, in the presence of an anthropologist who did not. Accuracy was ensured by the field assistants translating the questionnaires as a team, ensuring they understood the questions and were able to elicit accurate information from respondents. Checks were included in questionnaires – e.g. respondents were asked for their total number of births, living and deceased children, as well as for complete birth histories, so that discrepancies between answers could be picked up during the interview.

Complete birth histories were only collected from women, from which ages at first and last births, and inter-birth intervals have been calculated. The household head was also

questioned about household economics, including how households earned their living (what were the primary and secondary - where relevant - sources of income for the household). For farming households, information on farm size was collected as a measure of wealth. Additional information was collected on who owned the farm, and from whom they had inherited it (strictly speaking, land is owned by the village chief, but once families have been allocated land by the chief, they are able to pass it on to their heirs). Questions were also asked which indicated whether anyone in the household engaged in the monetary economic: whether anyone in the household engaged in paid work (if so, what kind), whether the household owned a business (if so, what kind), whether they owned fishing gear (which typically meant they engaged in commercial fishing), whether they traded any goods (if so, what), and whether they owned livestock (livestock holdings were generally low, most commonly a few chickens, sheep or goats). Each adult was also asked how many years of education they had completed (primary education became free in 1994).

Ethics

Malawi Data collection for this site was completed long before Institutional Review Board assessment was required. However, following local norms, the project was described at a community meeting and approved by community leaders. Subsequently, informed consent from individuals was obtained verbally prior to data collection.

Rural Afrocolombian, Emberá, and Mestizo Communities; Colombia

Ethnographic information

The data included here come from six Colombian communities living in four sites/locations. At the Coastal and Lowland sites, the population is composed of a majority of Colombians of African descent, along with minorities of Mestizos and indigenous Emberá. The data at each of these sites are based on censuses of one Afrocolombian and one Emberá community. At the Highland and Altiplano sites, the population is almost entirely Mestizo, and the censuses there include only one community per site.

Subsistence and labor practices are quite variable across sites. The Coastal Afrocolombian community relies on fishing and local wage labor for subsistence. There are, however, limited levels of hunting, horticulture, and animal husbandry, as well. The Emberá community subsists mostly on horticulture with some local wage labor. Both communities remain

separated from major urban centers, requiring either long-distance boat or air travel to reach. There is some level of market integration, however, as some fish are sold on the market through local intermediaries. The Lowland community is located in the rainforests of western Colombia, and subsistence for both Afrocolombians and Emberá is based on a mixture of horticulture, local wage labor, hunting, animal husbandry, and artisanal gold panning. The economy of the Highland community is based on small-scale agricultural production of coffee and sugarcane. The Lowland and Highland communities are neighbors, separated from one another by a 40-minute bus-ride; they are both separated from the nearest major urban center by long-distance bus rides of about 3–4 hours. Finally, the economy of the Altiplano community is based primarily on wage labor, especially in companies focused on large-sale flower cultivation. In contrast to the other sites, the Altiplano site is located near a major urban center (Bogotá), requiring only a short 1.5-hour bus ride to reach. Each community is characterized by some level of poverty relative to more urbanized areas in Colombia, especially the Coastal and Lowland sites—which are predominantly Afrocolombian and Emberá.

Data collection

The data presented here were collected by Cody Ross and local research assistants, as part of a wider, longitudinal field study on wealth inequality, demography, and social network structure. Data collection has been ongoing from 2016–2022. With wave 1 data collection occurring in 2016–2019, and wave 2 data collection starting in 2021. In total, the wave 1 database contains records on approximately 1,060 individuals in approximately 270 households. Complete reproductive history, social network, time allocation, and socio-economic interviews were conducted with approximately 540 adults in wave 1. In each site, we attempted to interview all adults residing within a pre-demarcated geographic region. In the Coastal, Lowland, and Highland communities, participation rate was very high (~95%); it was slightly lower in the Altiplano community (~75%).

We conducted reproductive history interviews with all adult household members. Each person was asked to document each child, in the order of their births, and provide their own age at the time of each birth. If individuals could not provide the exact date-of/age-at each birth, we attempted to use relative ages to reconstruct such ages. We also attempted to

triangulate and refine estimates on the basis of interviews conducted with multiple parents, and from children providing information about their siblings. We would always specifically enquire about child mortality. Any discordances in reports were adjudicated by Ross, often by re-interviewing relevant respondents in subsequent trips.

Time budget data were collected via self-report. Individuals were asked to report the amount of time they spend in specific activities on a typical workday: agriculture, animal husbandry, fishing, wage labor, home business, childcare, or ``other''. These data were normalized to yield the fraction of time that people spent in each labor category.

Ethics

All field protocols were approved by the Department of Human Behavior, Ecology, and Culture at the Max Planck Institute for Evolutionary Anthropology in Leipzig, Germany. Informed consent was obtained from each respondent prior to data collection, and from the community leader or local community council, when appropriate. Because of sometimes limited literacy, informed consent was obtained verbally after providing participants with a verbal description (in Spanish) of the research process and explaining how their data will be used (anonymously, for research purposes); in addition, all participants were provided with a written consent document.

Dolgan and Nganasan, Russian Federation

Ethnographic information

There are approximately 500 Dolgan and Nganasan living in the Ust'-Avam settlement in north central Siberia, Russian Federation. Ust'-Avam is situated north of the Arctic circle at the transition of a northern larch forest (Ary-Mas) and low shrub tundra. Administratively, Ust'-Avam is located in Dudinka District of Krasnoyarskiy Krai. Dolgan is the most northerly Turkic language. Nganasan is one of the four remaining Samoyedic languages. Prior to the construction of the Ust'-Avam settlement in the 1970s, the majority of Dolgan and Nganasan lived in extended families, kept herds of domestic reindeer in a number of kolkhozy (collective enterprises), and migrated on a seasonal rotations, while pursuing wild reindeer hunting, fishing, trapping, and trade. Reindeer herds were lost in the 1970s after settlement and establishment of the gospromkhoz (government hunting enterprise) (29). Today, nuclear

families in Ust’-Avam are mostly located in this small community living in government housing with several outlying households. Wild reindeer hunting and riverine and freshwater lake net fishing provide the primary sources of animal protein (90+% of animal protein comes from hunting and fishing) (30, 31). Furbearer trapping provides materials needed for winter clothing and a source of income through trade. Nearly all carbohydrates are imported into the community during summer on barges, except for berries that are gathered locally. Coal, diesel, lubricants and gasoline for snowmobiles are also imported on barges. Wage labor and salaried employment is common in the community as are social welfare payments.

Major changes in traditional descent and residence followed Soviet collectivization in the 1930s (32). Nganasan had bilateral cross-cousin marriage and Dolgan had an Omaha kinship system. Today, there is a good deal of intermarriage and both groups have a bilateral descent system, neolocal residence, and have dropped special kinship terms (33).

Previous research of the property system (34) showed a number of factors favouring common pool property and maintenance of earlier government-assigned territories after the collapse of the Soviet Union. Indigenous enterprises – *obshchiny* (collective holdings) – allocated in the 1990s were more involved with commercial sales of meat and fish, had better access to urban centres, and were more closely regulated through land, tax, and environment offices of local government. There is significant variability within the population in involvement in subsistence activities with some households producing quantities large enough to supply their own consumption, share with community members, and trade to the outside, while others produce little-to-no meat and fish (31, 35). Sharing within the community follows several phases and a number of non-market (sharing) strategies (35–37).

The Dolgan and Nganasan practice serial monogamy. Previous research found that the total fertility rate dropped from greater than 5 prior to the end of the USSR to a rate of 1.3 in 2003 and that short-term reproductive delay alone did not explain the dramatic drop in fertility (38). Age at first birth remained constant, and interbirth intervals increased moderately over that time period. Reproductive cessation was widespread, as the estimated fraction of women who ceased or indefinitely postponed reproducing doubled (for parities 2 through 4) or tripled (for nulliparous women).

Data collection

Data collection occurred September-June 1993-1994, November-June 1995-1996, September-November 1996, March-November 1997, May-November 2001, January-March 2003, and August 2007 conducted by John Ziker. In 1995-1996, JK initiated a base census, including information about employment, social welfare, and education for the entire community, which at that time numbered greater than 700 individuals. This work was based on the community's *pokhoziaistvenaia kniga* (household book) to which the settlement administration gave access, along with interviews of knowledgeable individuals. Ages and causes of death were documented in the household book based on official certificates of birth and death. For individuals born prior to official documentation, we followed the administration's convention of assigning birth days and months (January 1) to known or approximate years of birth. This census was updated in 1997, 2002, and 2007. Interviews were conducted in Russian by John Ziker who was fluent. Russian was the common language that all subjects also spoke and understood. Interview questions were translated and back translated. There were no issues with understanding questions relating to reproductive history.

Information on market integration and subsistence contribution to diet was obtained from 59 adults in 2003 as part of the background questionnaire for a cross-cultural cooperation project (39, 40).

Reproductive histories and kinship relationships were completed alongside verification of the census in 1997, 2002, and 2007. Age at first birth (AFB) was calculated using the difference between the mother's date of birth and that of her earliest born child for births from 1986 through 2007. Only cases (n=119) for which at least month and year of birth were known for both the mother and the child were included. These values were then averaged according to the birth year of the child. We also calculate a five-year trailing average of the focal year and the four preceding years, weighted by the number of observations in each year using data for the years 1982-2007.

Interbirth intervals (IBI) were calculated over the same period using the difference in dates of birth between consecutive children having the same mother. Only intervals for which at least month and year of birth were known for both children were included. Intervals longer than 8.7 years (mean IBI + 2 standard deviations) were assumed to be anomalous and were omitted (n=13). Although long intervals may reflect actual behaviour, we suspect some might also be due to incomplete registration of intervening births or erroneous birth dates and so chose to remove these outliers. The resulting intervals (n=224) were averaged by year for 1986–2007 according to the year of birth of the child closing the birth interval, along with five- year weighted trailing averages using data for the years 1982–2007.

Ethics

Ethical approval was provided by the Boise State University Institutional Review Board in 2007, and the University of Alaska Institutional Review Board in 2000. The research was carried out with permission from the local government and the Association of Indigenous Peoples of Taimyr. Informed verbal consent was obtained from all participants after explanation of the research objectives in the Russian language.

Himba, Namibia

Ethnographic information

Himba are semi-nomadic pastoralists living in the northwest corner of Namibia in a part of the Kunene region referred to as the Kaokoveld. The fieldsite for this project is the Omuhonga Basin, located about 150km from the regional capital of Opuwo. The Kaokoveld has an arid climate, increasingly affected by drought. There are about 50,000 Himba living across Namibia and Angola, with about 1000 people living in Omuhonga. Himba are closely related to Herero, a Bantu group who arrived in Namibia in the middle of the 16th century (41). They speak Otjihimba, a dialect of Otjiherero. Although English is the national language of Namibia, almost no Himba speak it and very little English is currently taught in Omuhonga schools. Himba continue to rely mainly on pastoral production for the majority of their calories. They herd cattle, goats and sheep. Women also have gardens where they grow maize, sorghum, squash and melons. Store-bought items like sugar, rice and pasta are increasingly being added to the diet, particularly during times of drought (42). Himba practice double descent, with wealth inherited mainly between men in the maternal line (e.g., mother's brother to sister's

son), while status and ritual responsibilities are passed patrilineally (43). Everyone is a member of both their maternal (*eanda*) and paternal (*oruzo*) clans. Himba are patrilocal and live in extended family compounds. Polygyny is common, with each co-wife maintaining her own hut and fire. However, women have substantial freedom of mobility and often spend significant periods of time in their natal compounds (44). Divorce and concurrent partnerships are both common and the group has a high rate of extra-pair paternity (45). Other social practices of importance include arranged marriage, the payment of brideprice, and fosterage. Fertility remains high, but contraceptive use is on the rise. Rates of schooling and access to formal healthcare are also increasing with a large secondary school in Omuhonga and the nearest clinic 15km away.

Data collection

Data collection occurred between 2010 and 2018, and was conducted by Brooke A. Scelza and Sean P. Prall. Household censuses were collected in 2010 and again in 2017, with additional demographic interviews conducted annually. Interviews were conducted by the Anthropologist with instantaneous translation from a local field assistant who spoke the language. Responses were recorded by the Anthropologist during the interview in English based upon those translations, with the opportunity to immediately ask for clarification and with follow-up questions. Dates and family relationships were cross-checked across multiple years of fieldwork with members of the same families and through multiple interviews with the same individuals across field seasons. The demographic database currently has information on >2000 individuals (46% male), including some who are now deceased, living in 39 household compounds. A local year-name system was used to age people (see (44) for details).

We conducted individual interviews to quantify demographics with 294 women included in this sample. Each woman was asked to document all births and age and sex all living children. Miscarriages and stillbirths were recorded to the best of our ability, although we suspect we are undercounting perinatal mortality, as this is a very sensitive topic. Wherever possible we asked husbands or women's kin about other births. We also collected paternity assertions, and classified children as being born: (a) within marriage, purportedly fathered by husband,

(b) within marriage purportedly fathered by someone other than the husband, (c) out-of-wedlock.

Cattle wealth was recorded for the head of each household and then linked to the women who live in that household. Women were asked whether they had a garden and then asked to provide a general estimate of relative size (small, medium, large). Education was assessed using a binary measure (ever went to school) since formal education among adults is very rare.

Ethics

Ethical approval was provided by the UCLA Research Administration (UCLA 10-000238) and carried out with permission from Himba chiefs in Omuhonga. Informed oral consent was obtained from all participants, with parental assent given for children under 16. All consents and interviews were conducted in Otjihimba with the help of local translators.

Khasi, India

Ethnographic information

The Khasi are a tribal group speaking a Mon-Khmer language and living in the eastern hills of the state of Meghalaya, in North-East India. They have followed a subsistence pattern of swidden agriculture on the hills and paddy cultivation in the valley floors in the past, continuing today with the growing addition of cash crops (ginger, broomstick, betal nut, and pineapple) and commercial enterprises such as various small businesses, driving taxis and lorries, small scale coal mining. A small number are also involved in professional pursuits and government services. The Khasi follow a matrilineal form of kinship reckoning, with the mother’s clan name being passed to her children. Daughters inherit land (if any is owned) from their mothers and the youngest daughter inherits the house and spiritual leadership of the lineage. Women are active economically - owning land, working the fields, selling and buying produce in the markets, running small businesses, hiring out as wage labor or even working in businesses or as professionals (a small minority). A large proportion of reproductive-age women also report themselves to be homemakers with no employment income or field cultivating activities. Poverty is common and possessions few.

Marriages are usually “love” unions and median age at first marriage is age 19 for women and age 23 for men. Husbands contribute labor in their wife’s fields and in house construction but usually bring no wealth initially to the marriage. They participate in wage labor and other jobs and so often have independent earning capacity from which they contribute to their wife’s and/or their matrilineal household. Women, after marriage, often live with their mothers for a time (sometimes until one or two children are born) before moving into houses built by their husbands. A husband may continue to reside with his natal family (contributing a portion of his earnings/produce to his wife’s household) or move in with his wife’s family on a permanent basis or until a separate home is constructed. Divorce is common with some women having children by three husbands. If men leave the marriage the house and land remains with the wife.

Data collection

The sample included Khasi women less than age 65 years (n=772, 105 were post-reproductive, >47yo). Interview data were recorded by local Khasi women we trained who were qualified by professional background (i.e., nursing, pharmacy, teaching, etc.). Data were collected on the composition of the households (including presence of a resident husband), reproductive histories of live births along with survival information on each child. Deceased children may not have been mentioned although we probed for such information and are fairly confident we recorded most of them. Miscarriages and stillbirths were probed for but confidence is low and they are not included. Included is which husband fathered each child. Husbands were recorded only if they fathered a child in the reproductive history of the woman, thus non-reproductive unions and their dissolution are not visible and not counted. Occupation and education of each household member and household agricultural production, and wealth (income, land, house quality and possessions) were recorded. Houses were graded from poor (thatched), to better (wooden/semi-pucca), to good (concrete). We asked about 11 items (hand cart, cycle, vehicle, scooter or motorcycle, radio, fan, fridge, telephone, tape, sofa, tv) with 99% of households holding less than three of these items. The percentage of men’s income given to his wife was asked. The only dietary measure is kg consumed of rice annually for each household.

A local field assistant trained as an anthropologist translated the content of the interview, and there were no issues with understanding as the questions on reproductive history were simple and straightforward.

Ethics

Research protocols were approved by the Human Subjects Committee at the University of Washington, Seattle Washington. Verbal consent was obtained prior to the interviews. Interviews were conducted by literate field assistants who spoke the local language and trained by the Anthropologist who did not.

Kipsigis, Kenya

Ethnographic information

Kipsigis are a Nilotic agropastoralist population living in southwestern Kenya, primarily in Kericho District, speaking a Kalenjin language. Although formally integrated into the Kenyan structure of locations and sublocations, the primary unit of significance is the neighbourhood (*kokwet*, a collection of dispersed homesteads across hillsides), governed by its elders council. Religious practice is highly eclectic, with a strong presence of Protestant and Catholic churches, and continued beliefs and practices associated with the traditional deity Asis and with family altars (*mabwaita*). Residence is strongly patrilocal, and inheritance of land and livestock strictly patrilineal. All men are organized by age set (*ipinda*), into which they are initiated through circumcision events. Similarly, women undergo clitoridectomy as they become eligible for marriage. Marriages are characterized by substantive bridewealth payments. Production is agropastoral; while livestock (cattle and goats) are central to ethnic identity, Kipsigis are reported to have farmed small gardens in all ethnographic reports, with maize introduced by the colonial authorities in the early 20th century. In the latter part of the 20th century commercial maize production grew substantially, as did the marketing of livestock products. At the time of study approximately 40% households sold some maize regularly into the market, and many fewer sold milk regularly (estimated at 5%). Men primarily benefit from the sale of these products although production depends on the work of men and women. A few men have additional sources of income (e.g., watchman, day labour) but there is very little outmigration for work. No women in the sample had regular income, although some would earn small amounts of cash from craft production, beer sales

or local sales of milk. Maize production during the study period was both by hand-held hoe, and with oxen-drawn hand-held wood or metal scratch ploughs. The majority of children were attending primary school during the study period, but secondary schooling, available adjacent to the study site, was attended by only a quite small proportion of children. Family sizes were still large with TFR across the province averaging 7.8 children, and there were very limited levels of family planning uptake in evidence.

Data collection

Borgerhoff Mulder collected both demographic and time allocation data across a 20-month period in 1982-1983, updating demographic records again in 1991. Time allocation data came from a full census of all households in four *kokwotinwek* (plural *kokwet*) collected between November 1982 and December 1983. The methodology was based on randomly timed behavioural scans, supplemented with reported data for absent household members; for the full methodology and rationales see (46), and for the full data see (47). The demographic and economic data were collected across a much larger sample of households between April 1982 and December 1983, based on an interview with the household head (male or female); the data were trimmed to match the time allocation sample, and the full the methodology is reported in (48, 49). Research permission was granted to Borgerhoff Mulder in 1981 from the Office of the President, Nairobi, Kenya.

Interviews were all conducted by the anthropologist competent conversationally in the local language (Kipsigis), with a local field assistant who was also a guide to the community who made introductions to each household and could, if necessary, translate any issues not understood by the anthropologist.

Ethics

The Kipsigis data included here were collected by Borgerhoff Mulder between 1981 and 1984 under annual permits from the Office of the President, Nairobi, Kenya. At the time of data collection, institutional review processes for demographic data collection were not in place at the researcher’s home institution. Individuals were nevertheless included in the sample only if they volunteered to participate in the study. All data are anonymized.

Makushi, Guyana

Ethnographic information

The Makushi inhabit the Rupununi savannahs of southwestern Guyana. Living along the border with Brazil, this ethnic group shares cultural traits with other groups from the Xingu basin. They include shifting cultivation centered on bitter cassava, matrilocal marriage, the performance of bride service, and fairly egalitarian gender relationships (50). Whereas premarital sex is not disapproved of, and is indeed an expected avenue to secure a partner (51), the Makushi generally marry monogamously and extended families typically share one residential area (52). Makushi marriages are endogamous, with mates usually selected from within the village community (51). As elsewhere in Guyana, differential migration affects family dynamics and has led to considerable between-community variation in the adult sex ratio (53), as men and women search for economic opportunities. Principle activities for men are mining, cattle ranching, agricultural work and logging, activities which occur mainly in the more remote areas of the Rupununi or in the forested regions at the center of the country, whereas women are attracted to urban areas (such as the capital of Roraima in neighboring Brazil) and the larger interior Guyanese towns (such as Lethem) in search of shop and domestic work (54). Such migration strongly affects the availability of potential mates for endogamous marriage but men are still expected to perform bride service in order to marry. This traditionally involved a year of service by the prospective husband in which he cleared and farmed fields for his in-laws while building a new dwelling nearby for himself and his wife. Men and women typically marry only once or twice, and conventions are similar across all marriages, with men providing bride service and thereafter bearing considerable responsibility to provide for their wives and children (including stepchildren who are valuable helpers) through farming, fishing and various forms of wage labor (51). At divorce, children remain largely the responsibility of the mother and her family, although they are provided for by a stepfather if she remarries.

Data Collection

Fertility and subsistence data were collected from January 2010 to April 2011 across eight Makushi communities. In each community, data collection began by way of a full census to determine community size and composition. Next, a minimum of 30 individuals from each village were randomly sampled (for a total of 148 men and 152 women) for in-depth

interviews on reproductive histories and household subsistence patterns. Interviews among the Makushi were conducted in English, which was spoken fluently by participants, by Ryan Schacht and a field assistant. To counter concerns regarding response-bias, we spent several months in each community building rapport and also gender-matched interviewers and interviewees. Individuals sampled were asked to share information regarding each of their children (e.g., birth dates, ages, birth order). All data was confirmed with local community health workers, who are responsible for reporting births and administering immunizations. Interviewees also reported monthly household income, size of farms, number, and types of livestock, as well sources of food that were consumed by the household (e.g., grown vs purchased).

Ethics

Ethical approval was provided by the UC-Davis IRB and all work was carried out only after receiving permission from the Guyanese Ministry of Amerindian Affairs and local government and tribal leaders in each of the eight communities.

Maya, Mexico

Ethnographic information

The Maya are subsistence maize farmers who live in the low canopy tropical forests of the Puuc region in the interior of the Yucatan Peninsula, Mexico. At the time of the study in the early 1990s used here, villagers cultivated maize, bean and squash, depended on some domestic animals, hunted game and collected forest plants. Honey and small quantities of maize might be exchanged for limited goods. Otherwise, no cash crops were grown. Families lived in wattle-and-daub, dirt-floor houses. There was no electricity, running water or access to motorized transportation. Each household grew its own food and furnished the labour to provision the household. Maya children lived and worked in their parents' households until they married and began families of their own in their late teens to early twenties. The Maya are life-long monogamous, no marriages dissolved because of divorce. If widowed, few remarry (Kramer, 1998, 2005).

As soon as children are able to walk, they frequently are found away from their homes visiting friends and relatives, running errands, working in the fields or accompanying their parent into

the forest. By the age of six to eight, Maya children participate in a variety of domestic and field activities. The increase in the time spent working then sharply rises at about age 10 and reaches a plateau, approaching adult levels of work in their mid-teens. While there was a rustic primary school, classes were infrequently held and school rarely interrupted children's work activities. Maya is a child's first language and the primary language throughout adulthood. Despite their predominant maize diet, the Maya are well-fed and in general good health. Child mortality was low (96% of children survive to reproductive age), and probably was aboriginally, in part because water is drawn from closed limestone wells (there are no rivers or flowing water in this region of the Yucatan), attenuating a primary vector of child mortality (55, 56).

Data collection

Longitudinal reproductive histories and censuses have been collected by Anthropologists Kramer and Greaves in this Yucatec Maya community since the 1990s. A cross-sectional sample of women who were married in 1993 is used here to quantify fertility characteristics under natural fertility and pre-market economic conditions. Reproductive history interviews were conducted by the Anthropologists in the Maya native language. During interviews, mothers were asked about their marital status, and to list their spouses, children from each marriage and to indicate whether they were living or deceased. The Maya are life-long monogamous, and seldom remarry after a spouse's death, which usually occurs in older age (Kramer, 2005). In the sample used here, one person had remarried due to the death of his spouse in his 20s. Interview questions were piloted and back translated with the assistance of a local Maya liaison, and reproductive histories have been verified and confirm during annual field visits between the 1990s and present day. The Maya are forthcoming in discussing marriages, deceased children, and spouses. Mothers were asked to include infants who had died very young in their list of children. Because of the difficulty to obtain consistent information about miscarriages (women define and think about differently), the count of children includes only full-term births.

The time allocation data for this study were collected in 1992-1993 as part of Kramer's dissertation research, and part of a longitudinal demographic and life history project. Instantaneous scan samples were collected over the course of a year. An observation period

lasted three to four hours, during which a participant's activity was recorded every 15 minutes. The observation day was limited by daylight hours from seven in the morning to six at night. Variables recorded during a scan sample included the individual, his or her activity, the object of the activity (a person, in the case of childcare), location, date, and time. Behaviors were coded using a hierarchical coding scheme that included over 400 possible activities. The sample included about 40% of village residents. Ages are known to the month and year.

Ethics

The Maya research was funded by the National Science Foundation (2051261, 0964031, 1632338), the NIH (AG 19044-01), the Milton Foundation, Harvard University, and the University of Utah. The Maya research has been approved by the Human Subjects IRB at Stony Brook University, Harvard University and the University of Utah, and by members and leaders of the Maya community. Informed consent was obtained from all subjects or, if subjects are under 18, from a parent and/or legal guardian.

Matlab, Bangladesh

Ethnographic information

Matlab, Bangladesh, is a rural area located in Chandpur District. Villagers in Matlab traditionally practiced agriculture, fishing, and small-scale trading (57, 58); however, these practices have been changing in recent decades in response to decreasing land ownership and increasing access to local, national, and international labour markets (59, 60). Agriculture has decreased as a primary occupation and villagers have increasingly adopted wage labour, primarily in construction, transportation, and business, within Matlab. At the same time, labour outmigration has become increasingly common among men who travel to cities in Bangladesh or abroad to work as unskilled or skilled laborers, primarily in the construction and maintenance sectors (58, 60). Household income is thus generated from a combination of agriculture, fishing, day labour, handicraft production, small businesses, salaried work, and remittances from family members working in cities or abroad (58). Average annual income was an estimated $1,584 in 2010 purchasing power arity dollars (61). Education levels vary considerably, but 30% of the population—primarily older people—has no schooling (58). Since the 1990s education has become more widely available, and a limited but growing

number of men and have obtained education-based employment. Education has also become more acceptable for women, a small number of whom have entered the labour market (62).

The majority of Matlab villagers are Muslim (89%); a minority are Hindu (11%). Patrilocal residence and patrilineal inheritance are most common, and families may be either extended or nuclear but often live in close proximity to kin in the context of a bari or patrilineal compound (63). Although polygyny is legal in Bangladesh, it is uncommon in Matlab and decreasing in frequency (64). Wealthier families' daughters marry later (often so they have time to attain higher levels of education; e.g. (65) and require large dowries to marry high-status grooms (66), while poorer families for whom dowry expenses are likely to be burdensome may marry daughters early to lower-status grooms in order to avoid higher dowry payments which may increase with the age of the daughter and perceived risks to her chastity (67).

Women in the area are primarily housewives (94.2% of women interviewed in 2018), whose major work entails cooking, housework and childcare; housewives may also engage in subsistence tasks including harvesting or processing of agricultural products, caring for chickens or ducks, or making handicrafts, though these tasks vary greatly across women. Women in rural Bangladesh have only entered the labour market in small numbers, so most women stay at home with their children as primary caregivers. Childcare help is also given by female relatives and neighbours (68), while fathers also contribute both direct care and high levels of indirect care and other male relatives also contribute indirect care (69).

The demographic transition in Bangladesh has been studied since the early 1980s. Between 1966 and 2019, total fertility rates have fallen from 6.7 to 2.6 children per woman. Conversely, life expectancy at birth has risen from 52.6 to 71.0 years for men and from 5.70 to 72.8 years for women, owing mainly to decreases in infant, child, and maternal mortality (70). Contraceptives have been available in the Matlab area since the 1970s; they were provided free to half of the population beginning in 1978 as part of a long-standing public health and family planning campaign by the International Centre for Diarrhoeal Disease Research, Bangladesh (ICDDR,B), but others in the area did not gain free access until the 1980s and 1990s. Contraceptive use rates in our sample were 15.9% at time of interview, and most

research suggests that the vast majority of women in the Matlab area use contraceptives either to space births or to cease reproduction once they have reached their desired number of children (71–73). Delaying first births with the use of contraceptives does occur, but it is relatively rare and violates local social norms that reward having a first child soon after marriage (74). Many women opt for tubal ligation after cessation of childbearing.

Data collection

Data were collected in rural Matlab, Bangladesh, by Shenk in collaboration with Alam and the International Centre for Diarrhoeal Disease Research, Bangladesh (icddr,b) in both 2010 and 2017– 2018. Icddr,b has run a large Health and Demographic Surveillance System (HDSS) site in Matlab since the late 1960s documenting demographic events (births, deaths, marriages, migrations) on an ongoing basis and collecting periodic socioeconomic survey data for a current population of approximately 200,000 people. The Matlab study area is divided into an ICDDR,B Area, where many public health interventions have been made, and a Government Service Area, functioning as a control area where more limited health services are available from the Bangladeshi government. Our sample was drawn randomly from a list of all women aged 20–64 in the HDSS sample. Equal numbers of women were drawn from the ICDDR,B Area and the Government Service Area and, within each area, from three 15-year age categories (20–34, 35–49, and 50–64), allowing for better representation of older women because rapid population growth in the Matlab area has resulted in much larger cohorts of younger women than older women in the population. Data were collected from 944 women in 2010 then follow-up surveys were conducted in 2018 with 765 of the same women; most attrition in the sample was due to family outmigration, mainly to cities in Bangladesh. Surveys covered topics such as family demographics, education, marriage, occupation, income, migration, health, and risk. Surveys were conducted via interview in the respondent's bari (family compound) and took around one hour to complete. Interviews were conducted by local field assistants for whom the local language of Bengali is their mother tongue. The survey was originally drafted in English, translated into Bangla (Bengali) by a local Bangladeshi researcher, then extensively pre-tested in the fieldsite, including updating of translations, by Shenk (who speaks intermediate Bengali) and a bilingual Bangladeshi researcher, until all concerns about question meaning, clarity, and translation had been resolved.

Ethics

All data were collected in accordance with human subjects research protocols approved by the Institutional Review Boards at the International Centre for Diarrhoeal Disease Research, Bangladesh, and either the University of Missouri or the Pennsylvania State University, and have therefore been performed in accordance with the ethical standards laid down in the 1964 Declaration of Helsinki. As specified in approved research protocols, all research participants gave informed consent prior to their inclusion in the study.

Maqu Tibetans, China

Ethnographic detail

Maqu County is an administrative district in the southwest region of Gannan Tibetan autonomous prefecture in Gansu province. Maqu is a part of Amdo Tibet and lies at the intersection of Qinghai, Gansu and Sichuan provinces. Maqu spans an area of about '10190 Square Kilometers and is home to 54900 people' according to a census in 2011. The average altitude of Maqu reaches '3500~3800 meters above sea level with an average temperature of 1.2 ℃ across the year and annual average rainfall of 611.9mm' (information from Maqu records). Most of the native people are 'Brog pa', herders making their living from selling yaks and sheep. Maqu, named after the Yellow River that runs through the County, is made up of one town and seven 'Xiang'. Nima town (meaning sun in Tibetan), is the political, cultural and economic centre of Maqu with several surrounding villages. A harsh environment on the Tibetan Plateau means Tibetan people preferably works in livestock production over traditional agriculture. The Tibetan grazing land has supported pastoralism for thousands of years with nomads moving at high frequency between seasons.

Data collection

Most of the data was conducted in the year 2014-2015 by Juan Du and local assistants. During this data collection period we conducted reproductive histories with all reproductively aged individuals. The interview was conducted by an anthropologist with the help of a local assistant. The anthropologist asked the local people directly in the local language about demographic data and occasionally received assistance from the assistant when the questions or answers were difficult to understand for either party. The anthropologist then wrote down the answers accordingly. We asked each individual the information of their parents, their

birth year, age and Chinese Zodiac (Chinese Zodiac helped estimate the birth year of the previous generation). The second part of the survey is about the herding system, the number of livestock, and the number of births and deaths of animals in the past year.

When collecting data on reproductive activities, males and females were separated to different spaces to avoid disturbance and get direct opinions from the male and female side, because females relied on their husband a lot, if asking questions to females while their husband around, they will concur with her husband's answer. It is also much easier for females to answer some private questions i.e., marriage and birth history and contraceptive use, without any male nearby. Each mother was asked to document each child, in order of their births, and their rough dates of births. Including live and death births/miscarriage.

Ethics

This research was approved by The College of Ecology Lanzhou University and University College London. Informed consent was given by all participants.

Mbendjele BaYaka, Republic of the Congo

Ethnographic Information

The Mbendjele reside in the rainforests of the Republic of Congo and are a subgroup of the BaYaka meta-population. Traditionally, their subsistence relies on hunting, trapping, gathering, honey collecting and fishing; and some exchange of forest products with neighbouring Bantu people for alcohol, cigarettes, and manioc (75). They are mobile with bilocal residence, politically egalitarian, and marriages are typically serially monogamous with some incidence of polygyny (5, 76). Like most immediate-return hunter-gatherers, cooperation in the domains of food sharing and childcare is prevalent (8, 77). Given the Mbendjele are in the process of socio-economic transition, there is substantial variation across communities with respect to levels of mobility, market integration, practice of horticulture and engagement in wage labour (78, 79). The total fertility rate for Mbendjele forest camps is 6.1.

Data collection

Data collection took place during July 2018 by Gul Deniz Salali, Nikhil Chaudhary (genealogy data), and Inez Derkx (camp scan data) in a hunter-gatherer camp in the Republic of Congo

for approximately 2 weeks. Salali and Chaudhary had full genealogies of many individuals from this camp from previous fieldworks in 2013 and 2014. In 2018, these genealogies were updated based on new individuals encountered and children born after 2014. The camp is situated beside a logging road in Congo's Ndoki forest and has an unstable camp size as the Mbendjele move between campsites based on seasons and food availability. In 2018 at the time of fieldwork, there were two Bantu families staying in the camp alongside Mbendjele, and in turn some trade and labour opportunities for people living there. Additionally, the Bantu had set up two stalls where alcohol, and some agricultural food such as manioc flour was available for sale.

Salali and Chaudhary had full genealogies of many individuals from this camp from previous fieldworks in 2013 and 2014. In 2018, further genealogical interviews were conducted with any participants they had not met before. In these interviews participants were asked to name their offspring, parents, parents' siblings, and grandparents. The questions were asked by a local translator in Mbendjele language, and either NC or GDS were present to draw up the genealogies, and to ensure all necessary questions were asked, understood, and translated appropriately. This was possible as both researchers were familiar with the Mbendjele translation of, and standard responses to, the questions. Moreover, responses were cross-checked with genealogical information that had been previously provided by related interviewees, this ensured there were no inconsistencies.

Prior to conducting the interviews, the researchers prepared the questions in French, which they and their translators can speak fluently. They then spent time with each translator and asked them to practice conducting the interview in Mbendjele on one of the other translators whilst also translating the responses back to the researchers in French.

At the time of data collection, this camp had 152 inhabitants of whom 66 were females and 86 males. As most individuals did not know their age, age category estimates were created based on age ranking and previous age estimate data for the same individuals. We collected genealogical information including reproductive histories and performed systemic camp scanning.

Camps scans were performed over a period of 9 days at 2-hour intervals throughout the day alternating daily between collection hours of 06:00-18:00 and 07:00-19:00 to prevent activity time bias. The resulting 55 camp scans all lasted between 45 and 90 minutes. During each scan, the activity of all observed individuals in and around the camp was recorded and classified into domestic, childcare, out-of-camp work and non-work activities. Most out-of-camp activities were not recorded unless an individual was within visible sight of the camp, for instance during children's playgroups in the nearby forest that could be heard from (near) the camp. Apart from those instances, a set route throughout the camp was followed for each scan. Activities were subsequently recorded when an encountered individual was observed performing a certain behaviour. If an individual was performing multiple behaviours simultaneously or occurred several times within one scan because they had moved within the scan period, all behaviours were recorded. In total, 148 individuals were sampled, including 65 females and 83 males.

During the genealogical interviews, interviewees were asked whether each named offspring was alive or deceased. In cases where one of the offspring was deceased, they were asked to name a current co-resident camp member who was of approximately the same life-stage or age as their child was when s/he passed away. They were also asked to specify the cause of death. From here, full reproductive histories were defined as total number of live births per individual and were collected for 39 females and 35 males.

Ethics

Ethical approval for this project was granted by the University College London Research Ethics Committee (UCL REC 1312/001). Additionally, all study participants gave free, prior and informed consent (FPIC) and the project was supported by local authorities and the Congolese Ministry of Scientific Research and Technology.

Mopan, Belize

Ethnographic information

An ethnographic and geographic description of Santa Cruz, Toledo, Belize, can be found in (80). By 2012 there were 428 residents (207 male, and 221 female) distributed in 82 households in Santa Cruz. Most of them spoke Mopan Maya, but a few also spoke Kekchi or Spanish at home. Considering household heads (ego subjects) only, the prevailing economic

activities were agriculture (3.75%), wage labor (1.74%), marketing (1.26%), reciprocal labor (1%), and foraging (0.77%). The community lands on which Mopan Maya subsist are situated in the uplands of Toledo District.

Data collection

During successive field seasons Kristina Baines, Carmen Cortez and Luis Pacheco-Cobos collected Time Allocation data from 2011-05-06 to 2013-05-03. (80) electronic supplementary material provides details of the Time Allocation (TA) and Household Demographic Survey (HDS) field methods that we used to record the fertility and subsistence information shared for the current cross-cultural analysis. Toward the end of his fieldwork LP-C's joined CC to conduct the HDS in 76 (92%) households. To complete the seven surveys' sections, one to three sessions with each household head were needed. The TA records and HDS survey forms have been coded by Chloe Atwater (81, 82) into a searchable R database. Interviews were conducted in English, Belize's official language, by the ethnographers affiliated to the project. Although ethnographers did not sustain fluent conversations in Mopan-Maya, the local language, they lived in the village and had good communication with collaborators. When elder women (> 60 years of age) were the household heads and did not speak English, a household member assisted the ethnographers with translations. Questions on reproductive history were clear to the interviewees, and pauses and clarifications were made by the anthropologists when needed, until both parts agreed with the context of the talk.

We made TA recordings every sixth day using a randomized spot-check methodology, for ten households and ten visiting times. Scans were conducted between 06:00 and 20:00 hours using 30 min intervals. We observed and recorded the activity for every member of the household. The activities reported by other household members were verified later with the individuals absent at the time of the visit. Our TA categories included: agriculture tasks, home tasks, firewood gathering, hunting and gathering, leisure, labor tasks, childcare, eating, meal preparation, religion, hygiene, sickness, school, marketing, visiting, unknown, and other.

The Household Membership section in our HDS includes all the reproductive history information shared by household heads. Exact marriage and birth dates were registered when available, though we mainly recorded the year. We asked interviewees to consider living and

deceased children during surveys. Other sociodemographic details (e.g., education, migration, land use) were also recorded during the HDS.

Ethics

Ethical approval was provided by the NSF (Division of Behavioral and Cognitive Sciences), Human Systems Dynamics, Collaborative Research. Development and Resilience of Complex Socioeconomic Systems: A Theoretical Model and Case Study from the Maya Lowlands [Proposal# 0827277]. Additionally, the Institute for Social and Cultural Research, Belize issued research permits ISCR/R/1/11 (6) from June 30th, 2011 to June 30th, 2012 and ISCR/R/1/12 (5) from September 28th, 2012 to September 28th, 2013. Consent was done verbally with household heads while running our Household Demographic Survey. Each of the team participants in the project, upon arrival in the field also got permission from the community's leaders to conduct research during a presentation in the community's assembly. Ethnographers participating in the project explained their research interests and plans for observing and helping community members with their daily activities (e.g., cultivating milpa, house building, hunting and gathering, cooking, etc.). Local authorities verbally agreed and authorized ethnographers to live in the village, walk freely and participate in residents' activities whenever they agree with them to do so.

Mosuo (matrilineal and patrilineal), China

Ethnographic information

The Mosuo are one of China's 55 ethnic minorities, with a population of over 40,000 individuals (83). Most Mosuo reside in southwestern China near the border of Sichuan and Yunnan Provinces in communities centered on Lugu Lake. Although best known for predominant matrilineal inheritance and descent, as well as a reproductive system that is less institutionalized than many (Shih, 2010), the Mosuo in fact have has two subpopulations with different kinship systems, including patrilineal enclaves that are geographically distinct from the matrilineal communities (Mathieu, 2003; Mattison, Sum, et al., 2021; Shih, 1993). Matrilineal villages are typically found in flat basins surrounding the Lake, whereas patrilineal villages are located at lower altitude in steeper geographical terrain (Mattison, MacLaren, et al., 2021). Patrilineal Mosuo practice patrilineal inheritance and descent, patrilocal stem family post-marital residence, and monogamous marriage. They share many features in common with the matrilineal Mosuo, including language and rites of passage, but differ in

terms of kinship and gender norms (89), as well as in aspects of economic development. Since the 1980s, the Mosuo have experienced intensifying integration with regional markets stemming primarily from increasing tourism (90–92). In areas with significant tourism, agriculture is giving way to tourism-based wage labor; this process is more prominent in matrilineal communities and more nascent in patrilineal ones (Mattison et al., in revision). The Mosuo are thus variably market integrated, with significant reliance on agriculture and animal husbandry to subsist in areas removed from markets.

Data collection

The research project furnishing these data is led by Dr. Siobhán Mattison of the Human Family and Evolutionary Demography Laboratory. Data were collected among matrilineal (300 households) and patrilineal (205 households) communities during the summer of 2017 and 2018. The data collection efforts associated with this dataset were led by Chun-Yi Sum.

Chun-Yi and her team conducted household interviews to characterize the socio-demographic characteristics of each household, as reported by a primary adult respondent. Each respondent was asked to document information on household-level characteristics, as well as those of each member of the household, including attributes related to gender, age, ethnicity, literacy, wealth (e.g., land size, material assets, income), education, migratory history, market integration (e.g., % calories consumed from market versus home-produced), and health. We depicted relationships among individuals based on identifying unique mothers and fathers for each person named in the dataset.

For Mosuo participants, interviews were conducted by the anthropologist (Sum), who is fluent in Mandarin Chinese, with assistance from a local field assistant, who translated as necessary to Naru, the local language. Simple language was used to ensure comprehension; many participants were literate and there is limited reason to believe that they would fail to understand our questions.

Ethics

Funding for the data collection was provided by the U.S. National Science Foundation (BCS 1920812). Ethical approval was provided by the UNM IRB and carried out with permission

from Fudan University's ethical review board. Informed consent was obtained from all participants.

Mayangna, Nicaragua

Ethnographic detail

The Mayangna are indigenous horticulturalists who reside in the forested areas of northeastern Nicaragua. They primarily live in sedentary communities along the rivers of the region. In terms of subsistence, the Mayangna complement their horticultural activities with fishing, hunting, and the consumption of small livestock. For monetary income, artisanal gold panning is the primary source of income for many households, though a smaller number of individuals earn money via mercantilism and government contracts as schoolteachers. Men are responsible for most agricultural activities and hunting. For other activities, including fishing and gold panning, women sometimes accompany their husbands and other male relatives to provide assistance (93). Wealth inequality is high. There are no permanent political positions. The Mayangna exhibit an uxorilocal residence pattern (94). Descent is traced bilaterally. At the time of data collection, fertility was high, with total fertility rates exceeding 7 births per woman (95). Before the age of 13, children rarely leave the community unaccompanied by older individuals. Adolescents occasionally embark on solitary excursions, such as gathering firewood or fishing, but in general, they are also likely to accompany older individuals. School is normatively compulsory for children between ages 5 and 12. At the time of data collection, participation in schooling by adolescents was sporadic and largely occurring via boarding schools outside of the study community.

Data collection

Observational data collection occurred over a yearlong study period from August 2004 to September 2005. Demographic data and reproductive histories were first collected by Jeremy Koster in 2004 and updated in 2013. Ages were estimated using the methods of anthropological demography (96).

Reproductive history interviews were conducted in the primary language of the interviewee, either Mayangna or Miskito. A tri-lingual research assistant served as the translator. Verbal responses were augmented by government birth records, which were available for most

children born after approximately 1993. The anthropologist was present for all interviews and asked follow-up questions as needed. Reproductive histories were conducted with all reproductive-aged adults in 2013 using standard methods (97). In addition to live births, the interviews also addressed miscarriages. At the time of data collection, access to contraceptive methods was limited.

Time allocation was documented using a version of instantaneous scan sampling from September 2004 to August 2005 in a community of approximately 25 households (93). The initial observation was scheduled randomly between 5:30 and 6:00, and then subsequent observations were scheduled every 30 minutes, concluding no later than 18:00. Observations were organized by household. During an observation, Koster documented the activities of all household residents. Households were sampled without replacement such that no household was observed more than once per day.

Ethics

Funding for fieldwork in Nicaragua was provided to JK by a Fulbright student grant, the National Science Foundation (Dissertation Improvement Award #0413037), the Hill Foundation, and a William Sanders dissertation grant. Ethical approval was provided by the UC IRB and carried out with permission from local government and tribal leaders in Nicaragua. Informed consent was obtained verbally from all participants.

Pemba, Zanzibar

Ethnographic information

Mitini (pseudoname) is a small village of approximately 40 households in the Southern Region of Pemba, Zanzibar, a politically autonomous region of the United Republic of Tanzania. Pembans are ethnically “Swahili people”, but as a result of Zanzibar’s complex maritime and imperial history constitute a mix of mainland, Arab and Persian ancestry. Religious affiliation is exclusively Islam, with multiple mosques and madrassa in the small village. Residence strongly patrilocal, inheritance largely patrilineal (following Islamic division of half shares to daughters), and polygyny permitted. Mitini, established in a government resettlement after a mid 1970s drought, lies in the rich western clove belt of the island although the soil is poor for agriculture. It is a relatively remote village, made up of four sub-villages (within 1500m of

each other), and much of the year can only be reached on a rough 3km trail through the hills from the ward town that lies on a small feeder road off the main North-South arterial road of the island. The ward town has a school and a couple of shops selling basics (about 15-20 products) brought by lorry from Chake Chake or Mkoani regional centers approximately 20/14 kms away. The village has one small kiosk with about 5-7 products usually available, brought by bicycle from the ward shop.

Farming is typically silva-culture, relying on multiple tree crops, primarily cloves. Cloves are harvested 2-3 times every two years (very irregular and unreliable), and are sold directly to a national marketing board. Also grown are a range of other spices and fruits, small amounts of cassava, and rarely rice or maize. Many households engage in small business, typically craft production (basket, mats, arrowroot, spices, dyes) or in providing services (traditional medicine, Islamic coaching, henna application, hair plaiting, cooking snacks, selling fruits, etc.) for small sums of money. About 85% of children were attending primary school, although many leave after 4-5 years. Secondary schooling is only available outside the study site (approximately at 7 km) as is attended by only a small proportion of children. Completed fertility appears to be very high, with no evidence whatsoever of use of family planning methods.

Data collection

Borgerhoff Mulder, with collaborator Emmanuel Maliti, Asha Yussuf Nour and Assaa Sharif Ngwali, collected data in July-August 2018 in a full census of the village as part of the ENDOW protocol focused on a full household membership census, economic status and social networks. Demographic data were collected, exclusively from all women aged >=15 years, by Borgerhoff Mulder and Nour, in conjunction with the ENDOW survey, with interviews focused on complete reproductive and marital histories.

Interviews were all conducted by the anthropologist competent conversationally in the local language (Swahili), with a local field assistant who was also a guide to the community who made introductions to each household and could, if necessary, translate any issues not understood by the anthropologist.

Ethics

Research was approved by the Second Vice President’s Office of the Revolutionary Government of Zanzibar (RGZ), and ethical clearance was obtained both through the University of California at Davis and the University of Cincinatti (IRB 1223476-1)
and informed verbal consent acquired from all participants.

Rural Poland

Ethnographic information

The study site is located in an economically disadvantaged area of Poland, characterised by centuries of peasant subsistence farming, and which was historically one of the poorest regions in Europe. The area is now undergoing a rapid transition away from farming and towards a more exclusive dependence on labour-market income sources. Although Poland has had a below-replacement fertility rate since the early 1990s, the study communities have remarkably higher fertility than national estimates would suggest (mean completed fertility in the sample is 3.81 [s.d. 2.15] children per woman). Households are multigenerational and range in size from one to 15 inhabitants, with an average of 5.36 inhabitants (s.d. 2.27) at the time of the survey.

Plot sizes today remain small; mean total land ownership in the sample is 2.33 hectares (s.d. 2.81) and there is large inequality in plot sizes with a right-skewed distribution; median plot size is 0.25 hectares. Mean arable plot size is just under one hectare (mean = 0.90, s.d. 1.35). Nonetheless, 65% of all sampled households derive some subsistence goods from their own cultivated produce, with 29% producing half or more of their household’s food in the winter and 35% producing half or more of their own food in the summer. Over 56% of farmers in the sample keep at least one cow, from which they can make their own cheese in addition to obtaining milk and cream (cattle are rarely used for meat). Bee keeping is also popular in the study area. 21% of farmers in the sample keep rabbits, 77% keep fowl, 8% sheep, 13% horses, and 15% pigs. Seasonal foods such as mushrooms and blueberries are gathered in the local forests, and those who own forested land can additionally use their wood for heating. Currently, over 65% of respondents (n = 1,255) live in households still practicing subsistence farming in addition to other income generating activities. However, there is a great deal of variation in income- generating strategies in this population, with 28% of households

subsisting mainly on state benefits, pensions and subsidies, and 65% deriving their principal source of income from the wage-earnings of householders.

Given the changing outlook for farming as the population becomes more dependent on wage-labour income, and as family sizes decrease, parents appear to be diversifying their investments away from the land, particularly focusing on the education of their children.

Data collection

Our sample of communities (21 villages and one town) was randomly drawn from four neighbouring municipalities containing a total of 34 potential study populations. All 22 of our sampled communities agreed to participate. We did not have access to a list of names or a map of households, so within each community every third house was sampled, with every adult woman (≥18 years) present in the house invited to take part in the survey. This strategy was further stratified in the town, by randomly selecting streets from a list obtained from the local government and approaching every third house/apartment on that particular street. We returned to houses that were unoccupied at the time of selection on up to three occasions. All consenting adult women were interviewed. All eligible women who declined to take part were noted as non-responders, as were women ineligible to take part due to age or illness. 52.4% to 89.4% of respondents who were approached in any village agreed to an interview, and the average response rate for the entire sample was 75% (total interviews ÷ [total responders + non-responders]).

Our sampling strategy means that important variables such as age are approximately normally distributed in all groups. The sample should not be considered representative of the country as a whole, but of the wider rural population of this particular region. Our samples are representative of the villages women inhabit and the aggregates we use are therefore appropriate to our research question.

Interviews were conducted by Colleran or by a trained local field assistant, in the Polish language, usually at the home of the respondent. Colleran spoke fluent Polish at the time of data-collection. The questionnaire was co-designed and written with Polish researchers, so

both back- and forward-translation was part of the initial design. Some changes were made after the pilot study to further tailor the questionnaire to local concepts and expressions.

Ethics

Data were collected between 2009–2010 as part of an anthropological- demographic project, approved by the Ethics Committee of the Department of Anthropology at University College London (UCL). Study aims and protocols were explained first to community leaders (in Polish) who themselves made a public announcement in local Churches prior to data collection in a particular village. Individual participants were provided with written documentation about the project aims and protocols and signed written consent forms before participating in interviews. All documentation and consent discussions were provided / carried out in Polish by Colleran or by a local field assistant.

Pumé, Venezuela

Ethnographic information

The Savanna Pumé are mobile hunter-gatherers indigenous to the llanos (savannas) of west-central Venezuela. The Savanna Pumé number about 650, dispersed in 24 bands over a 2800 km2 area. Because of the region's political instability, their geographic isolation and a poor terrestrial environment, the Savanna Pumé are largely buffered from outside encroachment and continue to live a hunting and gathering way of life. The Savanna Pumé are related to the River Pumé, their horticultural neighbours, with whom they exchange some goods, and infrequently marriage partners. The data for this study were collected in 2006 and 2007 by Anthropologists Kramer and Greaves as part of a longitudinal demographic and life history project (98, 99).

The Savanna Pumé move 5-6 times a year in response to changes in rainfall and the water table. During the six-month dry season, food is relatively abundant and subsistence centres on aquatic resources and wild fruit. When the llanos flood during the wet season, fish are difficult to locate and the subsistence base shifts to terrestrial game and tubers. Males, both adults and children, almost exclusively fish and hunt, while women and girls gather tubers and do most of the food processing. Both males and females collect wild fruit. Male- and female-foraged foods are both critical to the diet and are widely shared within and across

families. Women and children provide most of the childcare. The Savanna Pumé have no schools, and no access to health care or market foods. They a monolingual, and Pumé is a child's first and only language. Savanna Pumé women marry on average at age 15.1 (SD ±2.5; n=59) and males at age 18.0 (SD ±4.3; n=51). Although first marriages are often arranged, young people are not obliged to accept these matches, and have autonomy about when and whom they marry.

Data collection

Reproductive histories for all married Savanna Pumé women alive in 2007 and living in two bands were used to quantify fertility characteristics. Reproductive histories have been collected in these bands since the early 1990s, and most recently updated in 2005, 2006 and 2007. During interviews, mothers were asked about marital status, and to list their spouses, children from each marriage and to indicate whether they were living or deceased. The Pumé are forthcoming in their discussion of previous marriages, children from previous marriages, deceased children, and spouses. Mothers were asked to include infants who had died very young in their list of children. Because of the difficulty to obtain consistent information about miscarriages (women define and think about differently), the count of children includes only full-term births. Interviews were conducted by Anthropologist Greaves in the Pumé maí language.

The time allocation data were collected using instantaneous scan sampling in one band of Savanna Pumé. Daylight hours (from 7am to 6:30pm) were broken into three blocks, two of which were sampled each data collection day. During a block, a scan sample was recorded once an hour for each individual in camp. Variables recorded during a scan sample included the individual, his or her activity, the object of the activity (a person, in the case of childcare), location, date, and time. Children's behavior was coded using a hierarchical coding scheme that included over 400 possible activities. Although the Savanna Pumé do not use ordinal numbers greater than ten, ages are known to the year using several ethnographic methods including recording reproductive histories from multiple relatives and in kin terms, which are specific to sex, birth order and rank. Many children's ages can also be anchored to observed births during or shortly before field seasons.

Ethics

Funding for the Savanna Pumé research was provided by the National Science Foundation (0349963 and DBS-9123875, in 2004 and 2006, respectively), the L.S.B. Leakey Foundation (awarded to Russell Greaves, 1991), the Milton Fund and Harvard University. Informed consent was obtained from participants in accordance with Venezuelan local legislation and national guidelines from the Dirección de Asuntos Indígenas (DAI) of the Ministerio de Educación y Deportes, from CONICIT, and from the General Sectorial de Parques Nacionales (INPARQUES) of the Republic of Venezuela. Thumb prints from acknowledged leaders (both men and women) were obtained as documentation of permission to perform research in these communities. Procedures and consent scripts were approved by the IRB committees at Harvard University and at Stony Brook University.

Shodagor, Bangladesh

Ethnographic information

Shodagor communities in Matlab, Bangladesh are traditionally semi-nomadic, boat-dwelling fishers and traders who are culturally distinct from the majority ethnicity in the country. Matlab, the mostly rural sub-district where this research was conducted, is home to approximately 500 Shodagor families as well as 230,000 Bangladeshis who are majority Muslim, minority Hindu and primarily work as agriculturalists, wage laborers, and housewives and do not identify with the Shodagor ethnicity (ICDDR,B, 2018). Branches of the Meghna River make up the northern and southern borders of the region, which is also bisected by a second large river, the Dhonagoda River, its streams and canals. At the time of data collection (2014), the Dhonagoda was home to around 150 Shodagor families in five communities who are the primary focus of this study. These families reside on small, wooden houseboats, clustered within five distinct groups along the rivers and canals, or have moved onto the land within the previous 10 years and live in make-shift houses on the riverbanks.

Shodagor engage in a mixed cash and subsistence fishing economy. Ninety-five percent of men and forty-five percent of women in the Matlab Shodagor communities fish as their primary occupation. Most families report regularly eating some of their fish catch, but most fish caught are sold in the nearby markets in exchange for cash. Fifty percent of Shodagor women in Matlab work as traders, selling household goods door-to-door in the villages in

exchange for cash. Cash is then used to buy food and other goods in the markets. Shodagor families observe bilateral inheritance rules: Shodagor identity is inherited from fathers and property is passed from both mother and father to children. Shodagor postmarital residence patterns are multilocal, with families living virilocally (near husband’s kin), uxorilocally (near wife’s kin), near both spouses’ families, or neolocally after marriage. For semi-nomadic families, residence patterns change approximately twice per year after marriage. Most marriages are monogamous unions with only occasional polygyny, and most families live in nuclear family households in which most economic and childcare responsibilities are concentrated.

Data collection

Data collection took place over 9 months, between March and November, in 2014. During this time, a full census of boat-dwelling and newly land-dwelling Shodagor communities in Matlab, Bangladesh was conducted. All adults in all five of these communities (N = 170: 76 men, 94 women) were then asked to participate in interviews in which detailed demographic, economic, and parenting data were collected, in addition to full reproductive histories for all adults. During this time, observational data were collected via scan samples for all community members and focal follows of children under age 5, and anthropometric measurements were collected seasonally from all adults and children.

Demographic interviews were conducted with every adult in the study communities who consented to participation in the project (1 adult male and 1 adult female declined participation for no specified reason, and 3 adult females declined participation due to old age and illness). All participants were asked detailed information about their current household members, natal family members, marriage histories, and ever-born children. They were also asked about their annual movement patterns, land ownership, primary, secondary, and tertiary occupations, income earned from those occupations, time spent working in each, and consumption of fishing catches. All women were then asked about any pregnancies they ever experienced and the result of each pregnancy (miscarriage, stillbirth, live birth), as well as information about contraceptive use. Interviews were conducted by a field assistant who was a native speaker of Bangla, the local language, and also fluent in English. The Anthropologist Starkweather was also present for all interviews, but is not fluent in Bangla.

Questions were translated into Bangla prior to the interviews taking place with a great deal of discussion between field assistant and Anthropologist. Answers were recorded in English during each interview. The Anthropologist has enough proficiency in the Bangla language to understand respondents' answers on a basic level and any nuances were discussed in detail with the field assistant after the interview to ensure no information was lost.

Ethics

This research was reviewed and approved by the University of Missouri's Institutional Review Board, as well as the Research Review and Ethical Review Committees at the International Centre for Diarrhoeal Disease Research, Bangladesh (ICDDR,B). Informed consent was obtained from all participants. Child assent was obtained and parents signed informed consent for their children.

Rancheros: Baja California Sur, Mexico

Ethnographic information

There are approximately 4000 rural ranchers who live across the Sierra de La Giganta mountain range of BCS, Mexico. This is a hot, arid landscape with minimal infrastructure development and the only sources of fresh water come from desert springs and seasonal rains associated with the North American Monsoon (100). These people are descended from Euro-American colonists who first settled the region alongside Jesuit missionaries in the late 17th and early 18th centuries, followed by additional waves of colonization over the last 250 years (101). Households engage in goat and/or cattle ranching, with many keeping small gardens for crop production (102). There is considerable variation in the size and composition of herds which is determined by access to land and capital (103). Cattle are typically reserved for sale on commercial markets and require greater capital inputs relative to goats, while goats are often a staple food source. In addition to the sale of animals and their byproducts, non-local wage labor has become a secondary source of income for many families. Households also supplement their diet through governmental sponsored food programs and via goods purchased in regional markets. Descent is traced bilaterally and Eskimo kinship terminology predominates. Households are fairly independent and separated by large distances based on the location of desert springs (104). As a Catholic population, monogamy predominates and divorce is uncommon. Total fertility is 3.7 children with an average

interbirth interval of 3.6 years (104). If prospective mates are not present locally, males and females will seek marriage partners from other nearby ranching communities. As a result, post-marital residence is flexible and young couples may reside *with* the groom or bride's family, *near* the groom or bride's family, or in a separate location altogether. However, women are statistically more likely to disperse from their natal community relative to men following marriage (102).

Data Collection

Data collection occurred in the sub-delegation of Santa Maria de Toris which is located in the southern portion of the Sierra de La Giganta mountain range. Data were collected over two field seasons between July-August 2015 and May-July 2017, conducted by Shane J. Macfarlan and Juan Jose Garcia. During the first field season we conducted a community-wide census of all 32 households (population size: 113 individuals) in the community. In the second field season we conducted reproductive histories with all reproductively aged individuals. Accurate ages were established for all individuals by asking household heads their date of birth as well as that of their children and other household occupants.

We conducted household interviews to quantify demographics and household features with the mother of the household as well as other resident women with children. Each mother was asked to document each child, in order of their births, and their dates of births. We would always specifically enquire whether a child was living or dead (and the age at death, if applicable). During these interviews we also enquired about their herd size and composition, as well as the number of years each household member attended school (102). Interviews were conducted by our team of anthropologists who spoke the local language (Spanish). Two local field assistants initially assisted our anthropological team to use specific phrasings of questions on reproductive history to ensure no information was lost in translation.

Ethics

Ethical approval was obtained through the University of Utah Institutional Review Board office (IRB 00083096) and carried out with permission from the local governmental representative of Santa Maria de Toris. Informed consent was obtained from all participants via a verbal consent process performed in Spanish.

Tsimane, Bolivia

Ethnographic information

Tsimane are a subsistence-based society of over 16,000 forager-horticulturalists living in over 90 villages with a relatively low level of external market interactions. Horticultural fields containing a mixture of plantains, rice, corn and sweet manioc are fairly small (<1 hectares) and are left to fallow after several years of use, with new fields created based on availability, and ownership based on usufruct. In more acculturated villages, fields are often larger because rice is also sold as a cash crop. Fishing is common in all Tsimane villages located near water rivers, oxbow lakes or lagoons. Hunting with shotguns, rifles and bow and arrow is common in interfluvial villages. These foods together provide more than 90% of the calories in their diet, with the remainder coming mainly from store-bought items or trade with itinerant merchants. Villages are comprised of clusters of related households who often pool resources and labor.

The average Tsimane woman has 9.1 births over her reproductive lifetime (105), and effective contraceptive use is rare and inconsistent. Average ± SD age at first birth is 18.1± 2.7 years, with average ± SD interbirth interval of 30.7±10.6 months (4.8% < 18 months, 16.7% between 18 and 24 months)(106). Mean ± SD weaning time was estimated at 19.2 ± 7.3 months based on recall, although median weaning time based on prospective study is longer (27.5 ± 1.2 months)(107). Women commonly report breastfeeding up to and often after the onset of pregnancy.

Polygyny is widely accepted except in the two villages with an active missionary presence. Overall, only 5-10% of men are polygynously married, almost always with two sisters. Marriages are stable among the Tsimane with less than 20% of marriages ending in divorce (15 of 76 marriages begun over twenty years ago). Post-marital residence patterns tend to be matrilocal until the first child is born, followed by patrilocal residence. However, there is much flexibility in this pattern. Nuclear families are typically the unit of production, particularly for garden foods. Consumption occurs within extended family units living in close proximity to each other. Spouses engage in extensive cooperation and gender roles are well-defined. Women are responsible for providing childcare and preparing food and chicha (homemade

beer). Men acquire game and fish and engage in wage labour. Both genders collect forest fruits, fetch firewood and water, and work in horticultural gardens. Schools exist in most villages, although attendance is sporadic.

Tsimane villages vary in their degree of market access and interaction with Bolivian nationals. Acculturation takes several principal forms: visits to the main market town, San Borja and the selling of agricultural produce, wage labor with loggers or colonists, debt peonage with river merchants, and formal schooling. Portable radios that transmit messages and music from the New Tribes radio tower outside of San Borja are also available in many villages. Market items that are highly valued by the Tsimane include clothing, aluminum pots, utensils, salt, sugar, kerosene, and school supplies. Schools exist in over two third of all Tsimane villages, having been established anywhere from 2 to 20 years ago. Tsimane make occasional visits to San Borja during town festivals, and some sell agricultural produce or handicrafts. Near San Borja, some Tsimane work as farm hands for local ranchers. Along the upper Maniqui River, Tsimane sometimes collect jatata palm leaves and weave them into roofing panels. These panels are then traded with itinerant merchants who provide market goods and alcohol. The exchange rates vary among merchants, but most are unfavorably low.

Traditionally, there were no official leaders; older men and shamans wielded community-wide influence (108). Very few shamans remain today. In recent decades, Tsimane villages have adopted a system of elected chiefs (*corregidores*) and other officials in larger villages for representation purposes and interaction with outside interests. Chiefs wield no real power; their main tasks are to hold and conduct meetings in the event of conflicts, help organize community labor events, and represent village interests in transactions with outsiders. There is little accumulated wealth among Tsimane and no consistent, robust associations between market access and wealth inequality have been demonstrated (109). Income is earned through sporadic wage labor opportunities with loggers, merchants, and ranchers, while a small number of mostly men have been trained as bilingual elementary education teachers. Another source of wealth includes domesticated animals such as chickens, ducks, and in some rare cases, pigs and cows.

Data collection

Demographic interviews were conducted in the Tsimane language among all available adults over age 18 by Micheal Gurven during 14 months from July 2002 to August 2005 with the assistant of a bilingual Tsimane (Zelada). Deaths were elicited from retrospective reproductive histories of interviewees and their parents and siblings, whether alive or dead. This process yields redundant reproductive histories (e.g., if more than one sibling is interviewed), allowing for cross-validation of information. On the basis of these interviews, all living and deceased Tsimane' in the sample were assigned estimated ages. The Tsimane' have no taboos against speaking the names of deceased relatives, including small children.

Years of birth and death were assigned based on a combination of methodologies employed by researchers among the !Kung (110), Ache (111) and Hadza (112). These include using known ages from written records, relative age lists, dated events, photo comparisons of people with known ages and cross-checking of information from independent interviews of kin. Catholic missionaries have recorded the dates of 1,110 births among the Tsimane since 1952, many of the deaths occurring during the same period, and age estimates for an additional 120 individuals who were baptized as small children or as young adults during the early 1950s. We have also obtained birth records for an additional 310 individuals associated with the Evangelical Mission, La Cruz. For individuals born prior to record keeping, four procedures were used to ascribe ages to individuals from the reproductive histories.

The second method ranked all individuals, both living and deceased, in the sample of reproductive histories by relative age, beginning first with 5-year estimated age classes for relative age rankings. Multiple informants were used for each age class and inconsistencies were investigated and resolved. In addition, significant age-related relationships were investigated to augment the relative age lists. These include 'hip-child', hunting mentor, and playgroup companions.

Third, ages were also estimated using historical information and known historical events. A Catholic missionary, Father Marcelino, began working with the Tsimane' in 1952, and Father Martin Bauer in 1958. Both missionaries are widely known among most Tsimane in the Maniqui region. Another missionary was murdered in 1848, and many Tsimane scattered to

other regions downstream and in the interior forest back in the late 1920s. The first dirt highway was cleared in the interior forest in 1970 and then refurbished again in 1985. The Tsimane government organization started in 1989. We investigate which people were born and approximate ages of other individuals, such as younger siblings, or smallest child, with respect to these events. When interbirth intervals are short, as is common among the Tsimane, the use of sibling comparisons and dated events can be an effective tool in age estimation. A final method used a sample of seventy photos of individuals with known ages. For older individuals, fifty photos of men and women from ages 50 through 75 were used. These photos were used as a means of aging dead individuals at the time of their death, and for aging old interviewees. This method worked in conjunction with comparisons of dead individuals to known individuals in the community and surrounding region.

Each of the above methods provides a roughly independent estimate of age. When all estimates yield a date of birth within a 3-year range, the average was used unless one or two estimates were judged to be superior to the others. Individuals for whom confident ages could not be ascertained are not included in this analysis. These individuals are mostly people whose name appeared only once in the interviews, distant siblings without other interviewed kin in the sample, and estranged individuals not seen or heard from in many years.

Scan sampling was conducted in four communities between June 2002 and June 2003, and in an additional three communities throughout 2005 and 2006 by graduate students on the Tsimane Health and Life History Project (113). Both field assistants and researchers spoke Tsimane. To collect time allocation data, households were first divided into residential clusters, typically consisting of extended families. Each cluster was sampled randomly without replacement from 7:00 to 19:00 in three-hour time blocks in 2002-2003, and in two-hour time blocks in 2005. During these time blocks, the activity, location, and interactants were recorded every half-hour. An individual could be coded as participating in up to two activities simultaneously. For this study, only individuals who had more than 50 instantaneous scans were included in analyses. If coded activities were not directly observed by the researcher or later confirmed by the individual in question, the scan was not included in analyses. Individuals within the sampled age range for the present study who were parents were excluded. Household wealth describes the sum monetary value (based on the buying price in

the nearest town) of shotguns, rifles, watches, radios, bicycles, and domesticated animals among all nuclear family members. Men's responses were checked against women's responses (either to corroborate or to identify a discrepancy which was then resolved).

Ethics

Funding to support research in Bolivia was provided through the NIH and NSF. JS and HED thank the Tsimane for participating, and THLHP personnel for providing logistical support. JS and HED also thank Stacey Rucas, Jeff Winking, Amanda Veile, Lisa Levenson, Sara Mulville, and Chris von Rueden for collecting behavioural data. Consent was provided from the Tsimane government (Gran Consejo Tsimane), village leadership, and study participants. All procedures were approved by the IRB at the University of New Mexico and the University of California-Santa Barbara.

Extended methods

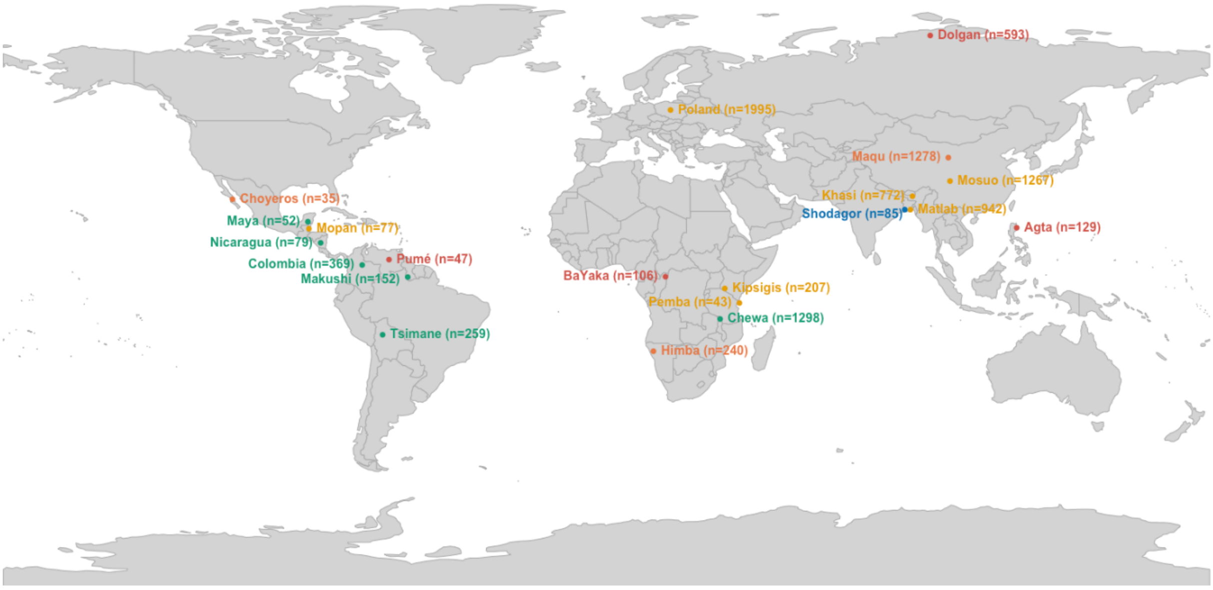

Figure S1 Map of the 27 study populations and sample sizes of women aged 14 and above years in each population, coloured by ethnographer-reported subsistence type. Hunter-gatherers (n = 4) are red, pastoralists (n = 3) orange, horticulturalists (n = 11) green, agriculturalists (n = 7) yellow and fishing (n = 1) in blue. N = 10,250. Please note, the countries with multiple groups from the same study are only noted by one country name.

Activity budgets

We created three variables from observational data on daily activities – *proportion of total production time spent foraging, farming* (to simplify analysis since rarely enough detail was collected to separate out 'horticultural' and 'agricultural' modes of production), and *wage*

labour – each of which are created by summing the number of instances each individual was observed to partake in each category, divided by the total number of directly observed production tasks witnessed. In a few instances, women were not documented to have undertaken any economic activities (e.g., they were mainly engaged in domestic and childcare tasks, which was common among mothers with infants). In this case, as we also had the activity budget data for her partner, we used this information as it was reflective of the household's investment in a given subsistence strategy.

Dietary sources

Researchers from 13 study sites asked participants about the sources of food consumed. This either took the form of daily food dairies, where at the end of the day households were asked what foods they had eaten that day and their source, or were asked to estimate the percentage of foods they consumed from different sources in general (e.g., 25% market, 75% farming). In the former, we summed the number of instances of food consumed from each source, dividing by the total consumed to create four variables: *proportion of diet from either foraging, farming* (again a combination of horticultural and agricultural food production) and *market purchases*.

Occupation

Household market integration was established from primary reported occupation during interviews in 18 study sites. In 5 sites, some women reported no occupation or labelled themselves as housewives. Here, we used the primary occupation from the partner matching our approach in the activity budgets. These data were reduced to a binary variable, representing subsistence occupations (agriculture, fishing, farming, pastoralism and hunter-gathering) versus market occupations (market labour).

Land, livestock, income and education

The remainder of the interview-based economic information came from four variables: *amount of land* (acres, n = 13)*, total livestock units* (summed count of smallstock (goats, sheep, pig) and largestock (cow and oxen) owned by a household, n = 18)*, annual reported income* (from wage labour and sell of cultigens in local currency – standardised within each population and log transformed, n = 15) and *educational attainment* (years - truncated at 12

years and above and coded as 13 due to lack of observations above this threshold, n = 24). Amount of land primarily denotes engagement in either cultivation while total livestock units captures investment in both cultivation and pastoralism.

Ethnographer reported information

Ethnographers provided a description of the spatial aspects of the residential units (n = 27) was reported to create the '*residence*' variable which was categorised at three levels: rural, transition and urban, following consultation with the data collection team. Secondly, ethnographers categorised the population according to one of the following subsistence strategies: hunter-gatherer, pastoralist, horticulturalist, agriculturalist and fisher. Finally, ethnographers ascribed degrees of market integration as either 'high', 'medium' or 'low' degrees of market integration.

Live births and ages at first and last birth variables

The majority of study sites provided reproductive histories at the level of the child, with their date of birth, death if applicable and parental information. From this we computed a summarised variable for all *live births* per mother. Any conception reported not to result in a live birth (e.g., spontaneous abortion) was not included in this variable. For all individuals who reported 0 live births we queried with each study site that these were true 0's rather than NAs (e.g., had not been interviewed directly, in which case they were removed from the analysis). Using mother's current age and children's date of birth we also computed the *age at first birth (AFB)* and *age at last birth (ALB)*. Several (n = 3) study sites provided this data already summarised. After processing, we (AEP and ER) confirmed these computed values with a number of checks: 1) if the age of first birth was below age 11 or age of last birth was above 50 we flagged the reproductive histories with the data collection team for that study site, who either corrected the dating errors, removed the observation due to lack of confidence or confirmed this was correct. Early (from age 10) and late births (up to age 60) have been retained in the analysis as a result of the relative distribution in AFB and ALB in the sample. 2) We confirmed that no individual's current age was younger than their reported ALB, where this was the case, we confirmed this was a rounding issue, or asked the data collection team to confirm dating. 3) We confirmed that all instances where the AFB equalled ALB, but individuals reported having two or more children was the product of a multiple

pregnancy. Where this was not confirmed, we asked the data collection team to review, remove or update the entire reproductive history of the relevant individual. Individuals without ages were removed from the analysis.

Modelling

We first modelled cumulative live births as a one-inflated Poisson process with rate parameter λ and one-inflation parameter θ. λ is modelled as a non-linear function of age using a growth function derived from (114) and recently used to model other aspects of human life history such as learning and foraging skill by (115–117):

$$\lambda = \text{Cumulative live births at age } x = \left[\left(1 - e^{-kx}\right)^{b}\right] \alpha$$

Where k controls the rate at which fertility increases with age (x), b is an elasticity parameter that controls the proportional change in fertility with age, and α is the scale parameter. α is a log-linear function of covariates other than age. We make the innocuous assumptions that fertility is zero at birth and that cumulative live births are monotonically increasing with age. Within the bounds of these constraints, many diverse fertility schedules can be described. We allow k and α to vary both between populations and between individuals. In all models, we include a linear effect of birth year on log(α) to account for differences in average fertility over calendar time. We assume b and θ are exogenous and thus only vary at the population level. The probability mass function was thus defined as:

$$(y_{n,i,j} | \lambda_{n,i}, \theta_j) = \begin{cases} \theta_j + (1 - \theta_j) \times \text{Poisson}(1 | \lambda_{n,i,j}) \text{ if } y_{n,i,j} = 1 \\ (1 - \theta_j) \times \text{Poisson}(y_{n,i,j} \mid \lambda_{n,i,j}) \text{ if } y_{n,i,j} \neq 0 \end{cases}$$

We also experimented with beta-binomial and zero-inflated likelihoods to capture potential overdispersion and excess zeroes in number of live births, but the over-dispersion and zero-inflation parameters were estimated to be extremely small, leading to poor model convergence indicative of misspecification. In our main analyses, we focus on how the predicted (mean) cumulative fertility varies between populations, individuals, and as a function of different subsistence and market integration predictors. We define the predicted cumulative fertility as: $\mathbb{E}[\text{CF}] = \mathbb{E}[\text{Live Births} \mid \text{Age} = 60]$. In the results, we denote the

posterior median of predicted cumulative fertility as $\widetilde{CF}$, and differences as $\widetilde{\Delta CF}$. In addition to these point estimates, we report 90% highest posterior density interval (HPDI) and the posterior probability (PP) that differences ($\widetilde{\Delta CF}$) are greater than or less than 0, depending on the hypothesis being tested.

All analyses were run in R (118) and all models were fit using the RStan package (119), which fits Bayesian mixed-effect models (accounting for population random effects) using Hamiltonian Markov Chain Monte Carlo. Markov chain convergence was assessed using standard diagnostics (number of effective samples, the Gelman-Rubin diagnostic, and visual inspection of trace plots). Our modelled estimates vary from previously published estimates since they are based on reported live births from both reproductive aged and post-reproductive women and thus are not directly comparable to classic measures of fertility (e.g., completed family sizes, total fertility rates)

DAG

Due to constraints, the DAG assumes that number of live births is a function of age, and that the values of subsistence/market integration do not change substantially with age (confirmed below in Figure S2). This is unlikely to be problematic as models adjusting for age close the path between age and subsistence. Age is a cause of mortality, which is a source of selection (S) in our study because we do not have complete fertility records for deceased individuals. The DAG also makes note of unobserved confounding (U) between number of live births and mortality, an issue given the potential risk of selection bias (e.g., if having more children reduces your chance of surviving to age 60, then women sampled at older ages are an increasingly narrow subpopulation). Birth year is an assumed cause of many other variables, namely mortality, number of live births and subsistence/market integration. Because we have sampled women of the same age (when their cumulative fertility is calculated) from different birth years, age and birth year are not perfectly correlated as they would be if we only had current age and number of live births. By including birth year in the modelling, we are able to capture broad cohort effects and secular trend which cause fertility to vary temporally.

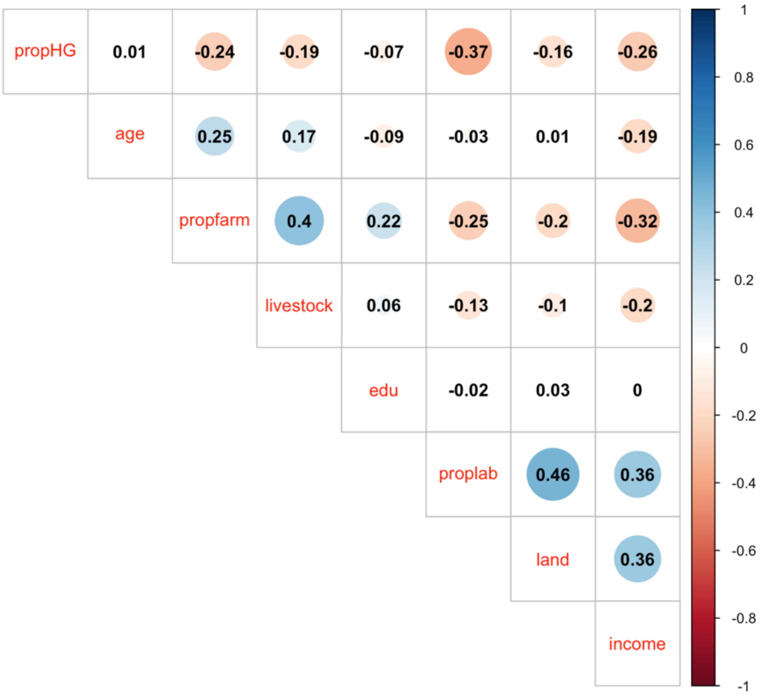

Figure S2 Correlation plot demonstrating the weak correlations between age and our measures of subsistence and market integration.

Our models, based on these causal assumptions are exploring the overarching relationship between fertility and subsistence and as a result do not include other, proximate, variables which exist on the causal pathway. Two such examples include infant mortality and contraception, both factors through which subsistence and market integration may impact fertility. Our research question asks whether subsistence activities are associated with fertility; the mechanisms through which subsistence is connected with fertility are unimportant in this model, only whether subsistence and fertility are – or are not – connected. As a result, it is not appropriate to include variables on the causal pathway in the models.

Extended results

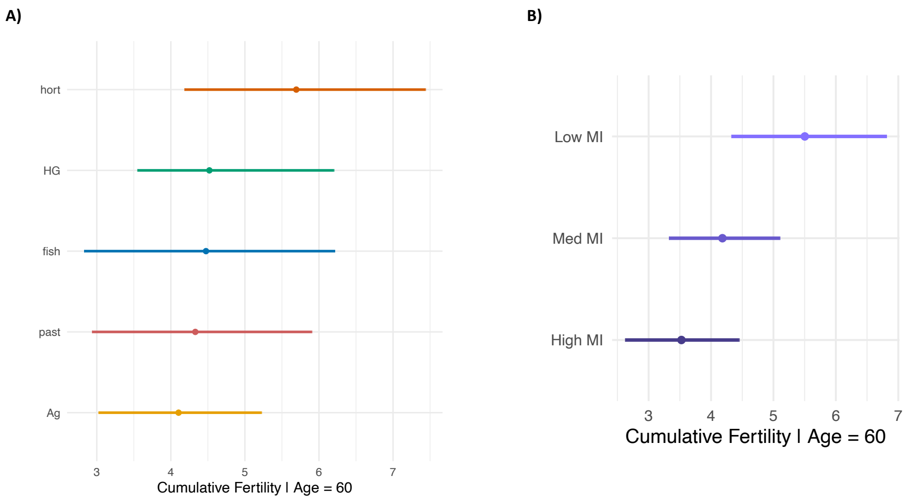

Figure S3 Group level comparisons for model predicted cumulative fertility at age 60 by **A)** subsistence type (colour coded) and **B)** ethnographer reported degree of market integration.

Table S1 Summary statistics for the entire sample (*n* = 10,025) across the independent and dependent variables separated by ethnographer reported subsistence type. Sample sizes vary by measure, documented in the table.

	**AGRICULTURALISTS (N=5298)**	**FISHERS (N=161)**	**HUNTER-GATHERERS (N=875)**	**HORTICULTURALISTS (N=2041)**	**WAGE LABOURERS (N=97)**	**PASTORALISTS (N=1553)**
**Age**						
**n**	5298	161	875	2041	97	1553
**Mean (SD)**	42.56 (16.62)	35.14 (16.80)	38.35 (18.22)	35.44 (14.96)	37.23 (16.17)	37.55 (17.16)
**Median (Min, Max)**	40.00 (14.00, 94.00)	32.00 (14.00, 88.00)	36.00 (14.00, 95.00)	32.00 (14.00, 90.00)	36.00 (16.00, 80.00)	34.00 (14.00, 96.00)
**Live births**						
**n**	5253	161	875	2038	97	1553
**Mean (SD)**	2.91 (2.39)	3.27 (2.73)	2.30 (2.59)	4.17 (3.08)	2.00 (2.17)	2.95 (2.53)
**Median (Min, Max)**	2.00 (0.00, 17.00)	3.00 (0.00, 12.00)	1.00 (0.00, 13.00)	4.00 (0.00, 15.00)	2.00 (0.00, 14.00)	3.00 (0.00, 13.00)
**Age at first birth**						
**n**	4219	136	537	1827	60	1203
**Mean (SD)**	22.31 (4.14)	17.69 (4.18)	22.93 (5.88)	19.34 (4.12)	20.73 (3.93)	20.61 (3.84)
**Median (Min, Max)**	22.00 (11.00, 46.00)	17.00 (11.00, 48.00)	22.00 (11.00, 44.03)	19.00 (10.00, 50.00)	20.00 (15.00, 36.00)	20.00 (11.00, 41.00)
**Age at last birth**						
**n**	2058	40	162	563	25	439
**Mean (SD)**	32.33 (6.32)	34.65 (8.78)	34.76 (7.91)	33.69 (8.86)	28.60 (7.01)	33.36 (7.51)
**Median (Min, Max)**	32.00 (16.00, 60.00)	34.50 (17.00, 52.00)	35.00 (12.00, 52.00)	34.33 (14.33, 55.00)	27.00 (17.00, 46.00)	33.00 (15.00, 52.00)
**% Wage labour**						
**n**	317	160	208	416	94	0
**Mean (SD)**	0.26 (0.31)	0.50 (0.45)	0.14 (0.25)	0.18 (0.32)	0.58 (0.48)	NA
**Median (Min, Max)**	0.15 (0.00, 1.00)	0.50 (0.00, 1.00)	0.00 (0.00, 1.00)	0.00 (0.00, 1.00)	1.00 (0.00, 1.00)	NA

**% Agriculture**						
**n**	317	160	208	416	94	0
**Mean (SD)**	0.13 (0.30)	0.00 (0.00)	0.00 (0.00)	0.00 (0.00)	0.00 (0.00)	NA
**Median (Min, Max)**	0.00 (0.00, 1.00)	0.00 (0.00, 0.00)	0.00 (0.00, 0.00)	0.00 (0.00, 0.00)	0.00 (0.00, 0.00)	NA
**% Horticulture**						
**n**	317	160	208	416	94	0
**Mean (SD)**	0.28 (0.28)	0.04 (0.14)	0.16 (0.25)	0.41 (0.39)	0.03 (0.16)	NA
**Median (Min, Max)**	0.27 (0.00, 1.00)	0.00 (0.00, 1.00)	0.00 (0.00, 1.00)	0.37 (0.00, 1.00)	0.00 (0.00, 1.00)	NA
**% Fishing**						
**n**	317	160	208	416	94	0
**Mean (SD)**	0.00 (0.00)	0.33 (0.43)	0.20 (0.32)	0.12 (0.23)	0.00 (0.00)	NA
**Median (Min, Max)**	0.00 (0.00, 0.00)	0.00 (0.00, 1.00)	0.00 (0.00, 1.00)	0.00 (0.00, 1.00)	0.00 (0.00, 0.00)	NA
**% Pastoralism**						
**n**	317	160	208	416	94	0
**Mean (SD)**	0.18 (0.21)	0.00 (0.00)	0.00 (0.00)	0.00 (0.00)	0.00 (0.00)	NA
**Median (Min, Max)**	0.11 (0.00, 1.00)	0.00 (0.00, 0.00)	0.00 (0.00, 0.00)	0.00 (0.00, 0.00)	0.00 (0.00, 0.00)	NA
**% Hunting-gathering**						
**n**	317	160	208	416	94	0
**Mean (SD)**	0.06 (0.10)	0.00 (0.00)	0.51 (0.40)	0.08 (0.20)	0.00 (0.00)	NA
**Median (Min, Max)**	0.00 (0.00, 0.75)	0.00 (0.00, 0.00)	0.48 (0.00, 1.00)	0.00 (0.00, 1.00)	0.00 (0.00, 0.00)	NA
**Income**						
**n**	3057	160	870	402	95	1278
**Mean (SD)**	62320.42 (110756.65)	72603.73 (107744.32)	3126.90 (19499.14)	405.30 (931.34)	939.44 (761.77)	0.00 (0.00)
**Median (Min, Max)**	32000.00 (0.00, 3000000.00)	13200.00 (0.00, 520000.00)	0.00 (0.00, 384000.00)	110.00 (0.00, 6975.00)	780.00 (0.00, 3509.00)	0.00 (0.00, 0.00)

**Market Intergration**						
**high**	3747 (70.7%)	161 (100.0%)	0 (0.0%)	119 (5.8%)	97 (100.0%)	0 (0.0%)
**low**	750 (14.2%)	0 (0.0%)	282 (32.2%)	1731 (84.8%)	0 (0.0%)	1518 (97.7%)
**medium**	801 (15.1%)	0 (0.0%)	593 (67.8%)	191 (9.4%)	0 (0.0%)	35 (2.3%)
**Land owned (arces)**						
**n**	4467	85	870	1391	0	1261
**Mean (SD)**	7.13 (11.34)	652.94 (1159.58)	0.44 (1.77)	5.52 (3.55)	NA	1981.50 (1587.40)
**Median (Min, Max)**	3.71 (0.00, 280.00)	0.00 (0.00, 5500.00)	0.00 (0.00, 17.30)	4.94 (0.00, 37.06)	NA	2038.58 (0.00, 7907.20)
**Livestock owned**						
**n**	4949	157	875	1991	97	983
**Mean (SD)**	11.07 (22.63)	0.77 (2.18)	0.00 (0.00)	0.93 (8.05)	0.40 (1.30)	88.11 (126.61)
**Median (Min, Max)**	1.00 (0.00, 553.00)	0.00 (0.00, 13.00)	0.00 (0.00, 0.00)	0.00 (0.00, 300.00)	0.00 (0.00, 8.00)	60.00 (0.00, 1145.00)
**% Market diet**						
**n**	3915	75	142	307	97	0
**Mean (SD)**	0.65 (0.32)	0.79 (0.25)	0.15 (0.25)	0.59 (0.37)	0.94 (0.18)	NA
**Median (Min, Max)**	0.75 (0.00, 1.00)	1.00 (0.50, 1.00)	0.03 (0.00, 1.00)	0.50 (0.00, 1.00)	1.00 (0.00, 1.00)	NA
**% Foraged diet**						
**n**	3915	75	142	307	97	0
**Mean (SD)**	0.00 (0.00)	0.00 (0.00)	0.33 (0.33)	0.04 (0.07)	0.00 (0.00)	NA
**Median (Min, Max)**	0.00 (0.00, 0.00)	0.00 (0.00, 0.00)	0.21 (0.00, 1.00)	0.00 (0.00, 0.50)	0.00 (0.00, 0.00)	NA
**% Farmed diet**						
**n**	3915	75	142	307	97	0
**Mean (SD)**	0.35 (0.32)	0.21 (0.25)	0.07 (0.18)	0.37 (0.35)	0.06 (0.18)	NA
**Median (Min, Max)**	0.25 (0.00, 1.00)	0.00 (0.00, 0.50)	0.00 (0.00, 0.71)	0.50 (0.00, 1.00)	0.00 (0.00, 1.00)	NA

**% Fished diet**						
**n**	3915	75	142	307	97	0
**Mean (SD)**	0.00 (0.00)	0.00 (0.00)	0.04 (0.11)	0.00 (0.00)	0.00 (0.00)	NA
**Median (Min, Max)**	0.00 (0.00, 0.00)	0.00 (0.00, 0.00)	0.00 (0.00, 0.50)	0.00 (0.00, 0.00)	0.00 (0.00, 0.00)	NA
**Urban residence**						
**rural**	3019 (57.2%)	97 (60.2%)	599 (85.8%)	1528 (74.9%)	97 (100.0%)	1530 (98.5%)
**transition**	498 (9.4%)	56 (34.8%)	96 (13.8%)	79 (3.9%)	0 (0.0%)	23 (1.5%)
**urban**	1757 (33.3%)	8 (5.0%)	3 (0.4%)	434 (21.3%)	0 (0.0%)	0 (0.0%)
**Education**						
**n**	5277	148	712	2004	86	1317
**Mean (SD)**	3.99 (3.64)	3.37 (4.63)	5.76 (4.67)	2.78 (3.34)	8.92 (4.39)	1.85 (1.77)
**Median (Min, Max)**	4.00 (0.00, 22.00)	0.00 (0.00, 17.00)	8.00 (0.00, 12.00)	2.00 (0.00, 21.00)	11.00 (0.00, 22.00)	1.00 (0.00, 12.00)

N.B. Market integration (high, medium and low) was defined by the ethnographers. Income was z-scored and log transformed in the analysis.

Table S2 Model results for each population's posterior median cumulative fertility at age 60 denoted by subsistence type and market integration.

**Posterior median**	**Lower 90% HPDI**	**Upper 90% HPDI**	**Population**	**Subsistence**	**MI**
7.36	6.13	8.77	Agta	HG	Low
8.6	7.34	10.03	Kipsigis	Ag	Low
3.63	3.2	4.14	BaYaka	HG	Low
8.83	7.84	9.77	Tsimane	hort	Low
2.52	2.18	2.9	Mestizo Altiplano	labour	High
3.83	3.32	4.4	Afrocolombian Coastal	fish	High
5.52	3.99	7.3	Embera Coastal	hort	Med
3.53	2.8	4.33	Mestizo Highland	Ag	High
3.98	3.49	4.4	Afrocolmbian Lowland	hort	High
5.39	3.76	6.97	Emebra Lowland	hort	Med
7.29	5.93	9.09	Nicaragua	hort	Low
2.29	2.14	2.47	Dolgan/Niaa	HG	Med
1.71	1.62	1.79	Matrilineal Mosuo	Ag	Med
1.73	1.62	1.87	Patrilineal Mosuo	Ag	Low
6.61	5.96	7.33	Maya	hort	Low
3.56	3.43	3.69	Bangladesh	Ag	High
4.48	3.79	5.23	Shodagor	fish	High
8.56	7.13	10.1	Pemba	hort	Low
5.71	4.84	6.53	Mopan	Ag	Low
2.61	2.22	3.1	Choyeros	past	Med
5.91	4.89	7.22	Makushi	hort	Med
6.61	5.52	8.05	Pumé	HG	Low
3.29	3.13	3.44	Maqu	past	Low
5.14	4.81	5.4	Chewa	hort	Low
6.59	5.99	7.3	Himba	past	Low
6.93	6.33	7.49	Khasi	Ag	High
3.08	2.99	3.18	Poland	Ag	High

Table S3: Model results for group-level subsistence type predicting group-level cumulative fertility at age 60

Posterior median	Lower 90% HPDI	Upper 90% HPDI	Age	Subsistence type
4.6	3.17	6.05	60	HG
4.09	2.99	5.16	60	Ag
5.61	4.1	7.28	60	hort
4.48	2.86	6.2	60	fish
4.33	2.87	5.8	60	past

Table S4: Model results for group-level market integration predicting group-level cumulative fertility at age 60

Posterior median	Lower 90% HPDI	Upper 90% HPDI	MI level	PP
5.5	4.32	6.81	Low MI	NA
4.18	3.33	5.1	Med MI	NA
3.53	2.62	4.45	High MI	NA
-1.96	-3.62	-0.34	diff_high_low	0.972

Please note all of the following model results are colour coded in line with the thresholds given in text to support our interpretation of the results. We interpret associations between predictions and outcomes as strong (green) when 90% of posterior distributions do not include 0 (e.g., PP equal to, or higher than 0.9), moderate (yellow) when 80% of posterior distributions do not include 0 and weak (blue) when less than 80% do not include 0.

Table S5 Model results for relationship between proportion of activities devoted to foraging (compared to other economic tasks) and cumulative fertility at age 60

Posterior median	Lower 90% HPDI	Upper 90% HPDI	Population	Subsist	MI	PP
-0.43	-0.88	-0.02	Agta	HG	Low MI	0.958
-0.04	-0.33	0.47	Kipsigis	Ag	Low MI	0.574
-0.31	-0.66	0.04	BaYaka	HG	Low MI	0.935
-0.4	-0.8	0.03	Tsimane	hort	Low MI	0.939
-0.03	-0.13	0.07	Nicaragua	hort	Low MI	0.774
-0.05	-0.19	0.09	Maya	hort	Low MI	0.818
-0.06	-0.22	0.06	Mopan	Ag	Low MI	0.889
-0.19	-0.57	0.24	Pumé	HG	Low MI	0.784
-0.35	-0.87	0.13	Average	Average		0.89

Table S6 Model results for relationship between proportion of activities devoted to farming (compared to other economic tasks) and cumulative fertility at age 60

**Posterior median**	**Lower 90% HPDI**	**Upper 90% HPDI**	**Population**	**Subsist**	**MI**	**PP**
0.24	0.01	0.46	Agta	HG	Low MI	0.973
0.29	-0.02	0.59	Kipsigis	Ag	Low MI	0.944
0.18	0.01	0.37	BaYaka	HG	Low MI	0.963
0.54	0.1	0.95	Tsimane	hort	Low MI	0.984
0.08	-0.01	0.16	Mestizo Altiplano	labour	High MI	0.937
0.17	-0.01	0.36	Afrocolombian Coastal	fish	High MI	0.94
0.24	-0.1	0.58	Embera Coastal	hort	Medium MI	0.895
0.14	-0.04	0.32	Mestizo Highland	Ag	High MI	0.895
0.15	0.02	0.31	Afrocolmbian Lowland	hort	High MI	0.97
0.49	-0.06	1.16	Emebra Lowland	hort	Medium MI	0.925
0.27	-0.12	0.61	Nicaragua	hort	Low MI	0.893
0.35	0.01	0.71	Maya	hort	Low MI	0.966
0.25	-0.03	0.54	Pumé	HG	Low MI	0.933
0.33	0.05	0.6	Average	Average		0.981

Table S7 Model results for relationship between proportion of diet from farmed sources (compared to foraged, fished or market sources) and cumulative fertility at age 60

**Posterior median**	**Lower 90% HPDI**	**Upper 90% HPDI**	**Population**	**Subsist**	**MI**	**PP**
0.15	-0.02	0.40	Agta	HG	Low MI	0.956
0.07	-0.05	0.21	Mestizo Altiplano	labour	High MI	0.847
0.10	-0.17	0.34	Afrocolombian Coastal	fish	High MI	0.729
0.26	-0.12	0.70	Embera Coastal	hort	Medium MI	0.905
0.23	0.02	0.53	Mestizo Highland	Ag	High MI	0.981
0.01	-0.22	0.24	Afrocolmbian Lowland	hort	High MI	0.524
0.25	-0.14	0.75	Emebra Lowland	hort	Medium MI	0.881
0.03	-0.03	0.08	Matrilineal Mosuo	Ag	Medium MI	0.791
0.00	-0.06	0.06	Patrilineal Mosuo	Ag	Low MI	0.509
0.14	0.05	0.23	Bangladesh	Ag	High MI	0.993
0.71	0.31	1.11	Makushi	hort	Medium MI	1
0.17	0.11	0.24	Poland	Ag	High MI	1
0.21	0.02	0.42	Average	Average		0.984

Table S8 Model results for relationship between log count of heads of livestock and cumulative fertility at age 60

Posterior median	Lower 90% HPDI	Upper 90% HPDI	Population	Subsist	MI	PP
0.54	0.15	1.01	Kipsigis	Ag	Low MI	0.991
0.04	-0.13	0.18	Mestizo Altiplano	labour	High MI	0.645
0.16	-0.08	0.37	Afrocolombian Coastal	fish	High MI	0.879
0.26	-0.05	0.6	Embera Coastal	hort	Medium MI	0.923
0.15	-0.06	0.38	Mestizo Highland	Ag	High MI	0.881
0.14	-0.1	0.37	Afrocolmbian Lowland	hort	High MI	0.846
0.13	-0.2	0.44	Emebra Lowland	hort	Medium MI	0.758
0.18	-0.22	0.58	Nicaragua	hort	Low MI	0.773
0.02	-0.03	0.08	Matrilineal Mosuo	Ag	Medium MI	0.781
0.03	-0.03	0.09	Patrilineal Mosuo	Ag	Low MI	0.779
0.32	-0.05	0.65	Maya	hort	Low MI	0.937
0.24	0.15	0.33	Bangladesh	Ag	High MI	1
0.09	-0.08	0.24	Choyeros	past	Medium MI	0.838
0.42	0.15	0.72	Makushi	hort	Medium MI	0.998
0.19	0.1	0.29	Maqu	past	Low MI	0.999
0.15	-0.13	0.42	Himba	past	Low MI	0.813
0.57	0.38	0.77	Khasi	Ag	High MI	1
0.24	0.18	0.3	Poland	Ag	High MI	1
0.18	0.07	0.29	Average	Average		0.997

Table S9 Model results for relationship between acres of land owned by the household and cumulative fertility at age 60

Posterior median	Lower 90% HPDI	Upper 90% HPDI	Population	Subsist	MI	PP
0.03	-0.42	0.49	Agta	HG	Low MI	0.551
0.41	-0.07	0.89	Kipsigis	Ag	Low MI	0.929
0.02	-0.04	0.08	Matrilineal Mosuo	Ag	Medium MI	0.738
0.08	0.01	0.16	Patrilineal Mosuo	Ag	Low MI	0.973
0.34	-0.04	0.75	Maya	hort	Low MI	0.929
0.03	-0.1	0.16	Bangladesh	Ag	High MI	0.632
0.16	-0.12	0.43	Shodagor	fish	High MI	0.84
0.27	-0.08	0.66	Mopan	Ag	Low MI	0.898
0.45	0.06	0.86	Makushi	hort	Medium MI	0.979
0.15	0.03	0.25	Maqu	past	Low MI	0.988
0.24	0.11	0.37	Chewa	hort	Low MI	0.999
0.88	0.61	1.19	Khasi	Ag	High MI	1
0.3	0.23	0.37	Poland	Ag	High MI	1
0.22	0.08	0.37	Average	Average		0.995

Table S10 Model results for relationship between proportion of activities devoted to wage labour (compared to other economic tasks) and cumulative fertility at age 60

**Posterior median**	**Lower 90% HPDI**	**Upper 90% HPDI**	**Population**	**Subsist**	**MI**	**PP**
-0.2	-0.55	0.24	Agta	HG	Low MI	0.782
-0.25	-0.53	0.03	Kipsigis	Ag	Low MI	0.937
-0.08	-0.21	0.09	BaYaka	HG	Low MI	0.812
-0.17	-0.44	0.18	Tsimane	hort	Low MI	0.788
-0.28	-0.5	-0.09	Mestizo Altiplano	labour	High MI	0.992
-0.39	-0.7	-0.1	Afrocolombian Coastal	fish	High MI	0.982
-0.21	-0.69	0.41	Embera Coastal	hort	Medium MI	0.736
-0.32	-0.63	-0.03	Mestizo Highland	Ag	High MI	0.974
-0.53	-0.88	-0.22	Afrocolmbian Lowland	hort	High MI	0.999
-0.47	-1.02	0.03	Emebra Lowland	hort	Medium MI	0.945
-0.11	-0.33	0.17	Nicaragua	hort	Low MI	0.764
-0.32	-0.68	0.06	Maya	hort	Low MI	0.931
-0.31	-0.57	-0.03	Shodagor	fish	High MI	0.961
-0.26	-0.62	0.2	Mopan	Ag	Low MI	0.827
-0.01	-0.01	0	Pumé	HG	Low MI	0.865
-0.32	-0.56	-0.07	Average	Average		0.976

Table S11 Model results for relationship between proportion of diet from market sources (compared to foraged, fished or farmed sources) and cumulative fertility at age 60

**Posterior median**	**Lower 90% HPDI**	**Upper 90% HPDI**	**Population**	**Subsist**	**MI**	**PP**
-0.03	-0.12	0.06	Agta	HG	Low MI	0.750
-0.07	-0.17	0.02	Mestizo Altiplano	labour	High MI	0.907
-0.1	-0.29	0.11	Afrocolombian Coastal	fish	High MI	0.784
-0.15	-0.42	0.12	Embera Coastal	hort	Medium MI	0.847
-0.14	-0.31	-0.01	Mestizo Highland	Ag	High MI	0.968
-0.04	-0.22	0.17	Afrocolmbian Lowland	hort	High MI	0.627
-0.16	-0.43	0.15	Emebra Lowland	hort	Medium MI	0.836
-0.05	-0.22	0.16	Dolgan/Niaa	HG	Medium MI	0.672
-0.04	-0.10	0.01	Matrilineal Mosuo	Ag	Medium MI	0.917
-0.02	-0.07	0.03	Patrilineal Mosuo	Ag	Low MI	0.754
-0.13	-0.22	-0.05	Bangladesh	Ag	High MI	0.993
-0.52	-0.81	-0.23	Makushi	hort	Medium MI	1
-0.16	-0.22	-0.10	Poland	Ag	High MI	1
-0.16	-0.28	-0.03	Average	Average		0.968

Table S12 Model results for relationship between reporting a salaried occupation (compared to subsistence-based occupations) and cumulative fertility at age 60

**Posterior median**	**Lower 90% HPDI**	**Upper 90% HPDI**	**Population**	**Subsist**	**MI**	**PP**
0.01	-0.3	0.38	Dolgan/Niaa	HG	Medium MI	0.524
-0.03	-0.14	0.09	Matrilineal Mosuo	Ag	Medium MI	0.674
-0.11	-0.25	0.02	Patrilineal Mosuo	Ag	Low MI	0.908
-0.35	-1.4	0.96	Maya	hort	Low MI	0.699
-0.26	-0.45	-0.05	Bangladesh	Ag	High MI	0.986
-0.34	-0.85	0.22	Shodagor	fish	High MI	0.857
-0.48	-1.63	0.71	Pemba	hort	Low MI	0.752
-0.23	-0.91	0.5	Mopan	Ag	Low MI	0.708
0.01	-0.42	0.58	Choyeros	past	Medium MI	0.514
-1.09	-1.88	-0.4	Makushi	hort	Medium MI	0.997
-0.47	-0.87	-0.04	Chewa	hort	Low MI	0.959
-1.07	-1.56	-0.59	Khasi	Ag	High MI	1
-0.53	-0.67	-0.4	Poland	Ag	High MI	1
-0.29	-0.55	0.01	Average	Average		0.953

Table S13 Model results for relationship between urban versus rural residence and cumulative fertility at age 60

Posterior median	Lower 90% HPDI	Upper 90% HPDI	Population	Subsist	MI	PP
-0.22	-0.91	0.33	Agta	HG	Low MI	0.804
-0.12	-0.7	0.52	Tsimane	hort	Low MI	0.693
-0.57	-1.38	-0.09	Afrocolombian Coastal	fish	High MI	0.989
-0.2	-0.72	0.1	Mestizo Highland	Ag	High MI	0.916
-0.23	-0.66	0.09	Dolgan/Niaa	HG	Medium MI	0.911
-0.06	-0.18	0.06	Matrilineal Mosuo	Ag	Medium MI	0.801
-0.05	-0.16	0.04	Patrilineal Mosuo	Ag	Low MI	0.87
-0.21	-0.7	0.12	Shodagor	fish	High MI	0.884
-0.09	-0.35	0.12	Choyeros	past	Medium MI	0.838
-0.49	-1.13	0.21	Makushi	hort	Medium MI	0.898
-0.3	-0.65	0.09	Chewa	hort	Low MI	0.881
-1.02	-1.64	-0.43	Khasi	Ag	High MI	1
-0.3	-0.49	-0.13	Poland	Ag	High MI	0.997
-0.34	-0.66	-0.06	Average	Average		0.979

Table S14 Model results for relationship between years spent in education and cumulative fertility at age 60

Posterior median	Lower 90% HPDI	Upper 90% HPDI	Population	Subsist	MI	PP
-0.09	-0.73	0.18	Kipsigis	Ag	Low MI	0.885
-0.05	-0.3	0.04	BaYaka	HG	Low MI	0.908
-0.03	-0.36	0.16	Tsimane	hort	Low MI	0.673
-0.17	-0.44	0	Mestizo Altiplano	labour	High MI	0.996
-0.74	-1.5	-0.04	Afrocolombian Coastal	fish	High MI	0.997
-0.3	-1.3	0.3	Embera Coastal	hort	Medium MI	0.863
-0.5	-1.11	-0.04	Mestizo Highland	Ag	High MI	0.998
-0.69	-1.32	-0.12	Afrocolmbian Lowland	hort	High MI	1
-0.07	-0.5	0.1	Emebra Lowland	hort	Medium MI	0.887
-0.63	-2.45	0.47	Nicaragua	hort	Low MI	0.893
-0.19	-0.57	0.06	Dolgan/Niaa	HG	Medium MI	0.853
-0.06	-0.2	0.04	Matrilineal Mosuo	Ag	Medium MI	0.891
-0.12	-0.35	0.06	Patrilineal Mosuo	Ag	Low MI	0.908
-0.03	-0.3	0.2	Maya	hort	Low MI	0.674
-0.42	-0.68	-0.17	Bangladesh	Ag	High MI	1
-0.02	-0.27	0.05	Shodagor	fish	High MI	0.914
-0.64	-2.36	0.13	Pemba	hort	Low MI	0.96
-0.24	-0.87	0.15	Mopan	Ag	Low MI	0.879
-0.18	-0.59	0.05	Choyeros	past	Medium MI	0.93
-0.33	-0.91	0.01	Makushi	hort	Medium MI	0.982
-0.02	-0.16	0.07	Maqu	past	Low MI	0.765
-0.06	-0.27	0.07	Chewa	hort	Low MI	0.83
-0.02	-0.16	0.13	Khasi	Ag	High MI	0.688
-0.82	-1.16	-0.46	Poland	Ag	High MI	1
-0.47	-0.83	-0.15	Average	Average		0.999

Table S15 Model results for relationship between annual income and cumulative fertility at age 60

Posterior median	Lower 90% HPDI	Upper 90% HPDI	Population	Subsist	MI	PP
0.09	-0.38	0.56	Agta	HG	Low MI	0.626
0.06	-0.11	0.24	Mestizo Altiplano	labour	High MI	0.706
-0.03	-0.32	0.24	Afrocolombian Coastal	fish	High MI	0.577
0.14	-0.39	0.67	Embera Coastal	hort	Medium MI	0.691
0.42	-0.06	0.97	Mestizo Highland	Ag	High MI	0.952
-0.28	-0.53	-0.06	Afrocolmbian Lowland	hort	High MI	0.979
0.12	-0.46	0.82	Emebra Lowland	hort	Medium MI	0.64
0.15	0.08	0.24	Dolgan/Niaa	HG	Medium MI	0.999
0	-0.06	0.06	Matrilineal Mosuo	Ag	Medium MI	0.514
0	-0.07	0.08	Patrilineal Mosuo	Ag	Low MI	0.537
-0.14	-0.53	0.28	Maya	hort	Low MI	0.708
-0.14	-0.24	-0.05	Bangladesh	Ag	High MI	0.994
0.13	-0.15	0.44	Shodagor	fish	High MI	0.782
-0.53	-0.91	-0.14	Makushi	hort	Medium MI	0.989
-0.04	-0.27	0.18	Khasi	Ag	High MI	0.629
0.01	-0.13	0.16	Average	Average		0.554

Sensitivity Analysis

Table S16 Model results for relationship between heads of livestock and cumulative fertility at age 60, controlling for household wealth.

Posterior median	Lower 90% HPDI	Upper 90% HPDI	Population	Subsist	MI	PP
0.04	-0.13	0.19	Mestizo Altiplano	labour	High MI	0.643
0.17	-0.08	0.4	Afrocolombian Coastal	fish	High MI	0.876
0.26	-0.1	0.6	Embera Coastal	hort	Medium MI	0.906
0.15	-0.1	0.37	Mestizo Highland	Ag	High MI	0.87
0.15	-0.09	0.39	Afrocolmbian Lowland	hort	High MI	0.855
0.11	-0.23	0.43	Emebra Lowland	hort	Medium MI	0.717
0.19	-0.28	0.62	Nicaragua	hort	Low MI	0.755
0.02	-0.03	0.07	Matrilineal Mosuo	Ag	Medium MI	0.765
0.02	-0.04	0.09	Patrilineal Mosuo	Ag	Low MI	0.73
0.33	-0.03	0.69	Maya	hort	Low MI	0.932
0.23	0.14	0.33	Bangladesh	Ag	High MI	1
0.09	-0.09	0.24	Choyeros	past	Medium MI	0.81
0.42	0.14	0.73	Makushi	hort	Medium MI	0.995
0.2	-0.12	0.49	Himba	past	Low MI	0.862
0.63	0.42	0.83	Khasi	Ag	High MI	1
0.23	0.17	0.29	Poland	Ag	High MI	1
0.17	0.05	0.28	Average	Average		0.989

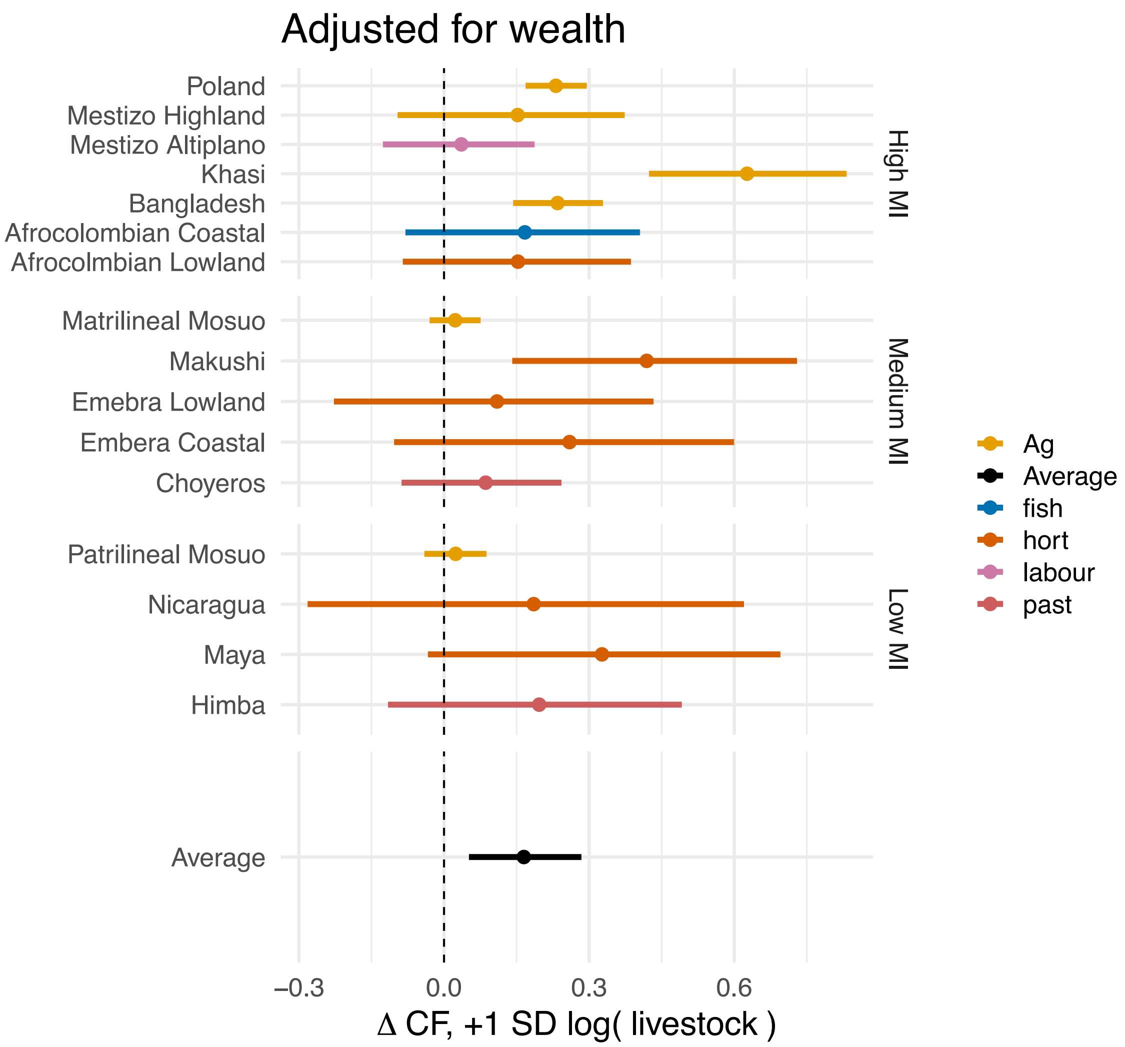

Figure S4: Plot of the relationship between livestock and fertility, controlling for wealth

Table S17 Model results for relationship between land and cumulative fertility at age 60, controlling for household wealth.

**Posterior median**	**Lower 90% HPDI**	**Upper 90% HPDI**	**Population**	**Subsist**	**MI**	**PP**
0.12	-0.37	0.61	Agta	HG	Low MI	0.663
0.03	-0.04	0.09	Matrilineal Mosuo	Ag	Medium MI	0.77
0.09	0.01	0.16	Patrilineal Mosuo	Ag	Low MI	0.972
0.34	-0.08	0.76	Maya	hort	Low MI	0.914
0.05	-0.1	0.2	Bangladesh	Ag	High MI	0.712
0.16	-0.15	0.47	Shodagor	fish	High MI	0.814
0.31	-0.11	0.72	Mopan	Ag	Low MI	0.895
0.48	0.05	0.92	Makushi	hort	Medium MI	0.974
0.14	0.02	0.25	Maqu	past	Low MI	0.984
0.92	0.63	1.21	Khasi	Ag	High MI	1
0.3	0.24	0.38	Poland	Ag	High MI	1
0.22	0.05	0.38	Average	Average		0.99

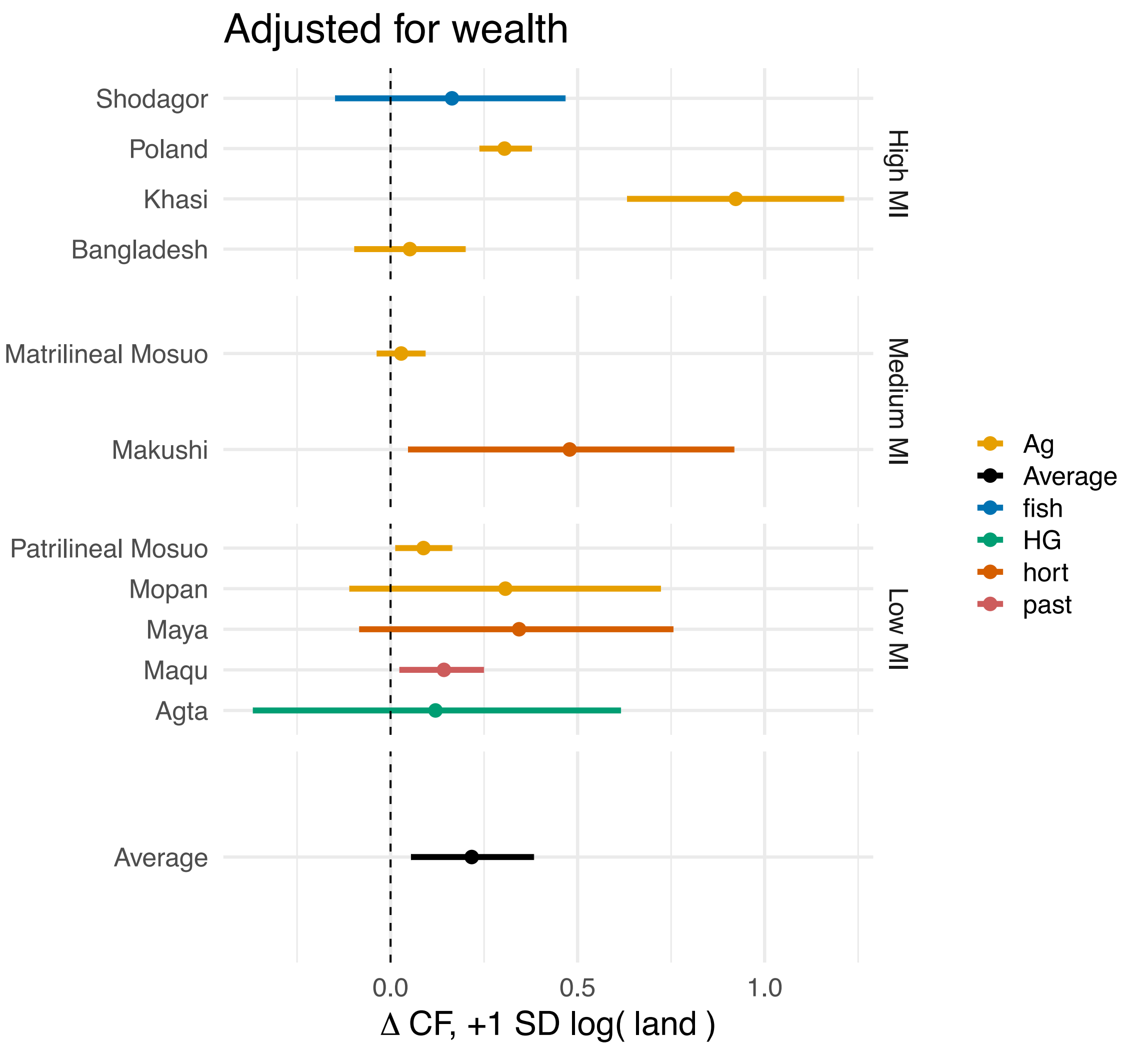

Figure S5 Plot of the relationship between land owned and fertility, controlling for wealth

Table S18 Model results for relationship between biomedical contraceptive usage (binary, yes or no at least once in lifetime) at age 60, controlling for household wealth.

**Posterior median**	**Lower 90% HPDI**	**Upper 90% HPDI**	**Population**	**Subsist**	**MI**	**PP**
0.22	-0.18	0.67	Mestizo Altiplano	labour	High MI	0.818
0.31	-0.28	0.96	Afrocolombian Coastal	fish	High MI	0.812
0.26	-0.81	1.46	Embera Coastal	hort	Medium MI	0.665
-0.25	-1.22	0.53	Mestizo Highland	Ag	High MI	0.715
0.52	-0.08	1.17	Afrocolmbian Lowland	hort	High MI	0.924
0.34	-0.8	1.67	Emebra Lowland	hort	Medium MI	0.715
0.58	0.16	1	Bangladesh	Ag	High MI	0.988
0.54	-0.17	1.22	Shodagor	fish	High MI	0.912
-0.46	-1.24	0.28	Khasi	Ag	High MI	0.847
-0.1	-0.28	0.1	Poland	Ag	High MI	0.808
0.21	-0.24	0.7	Average	Average		0.798

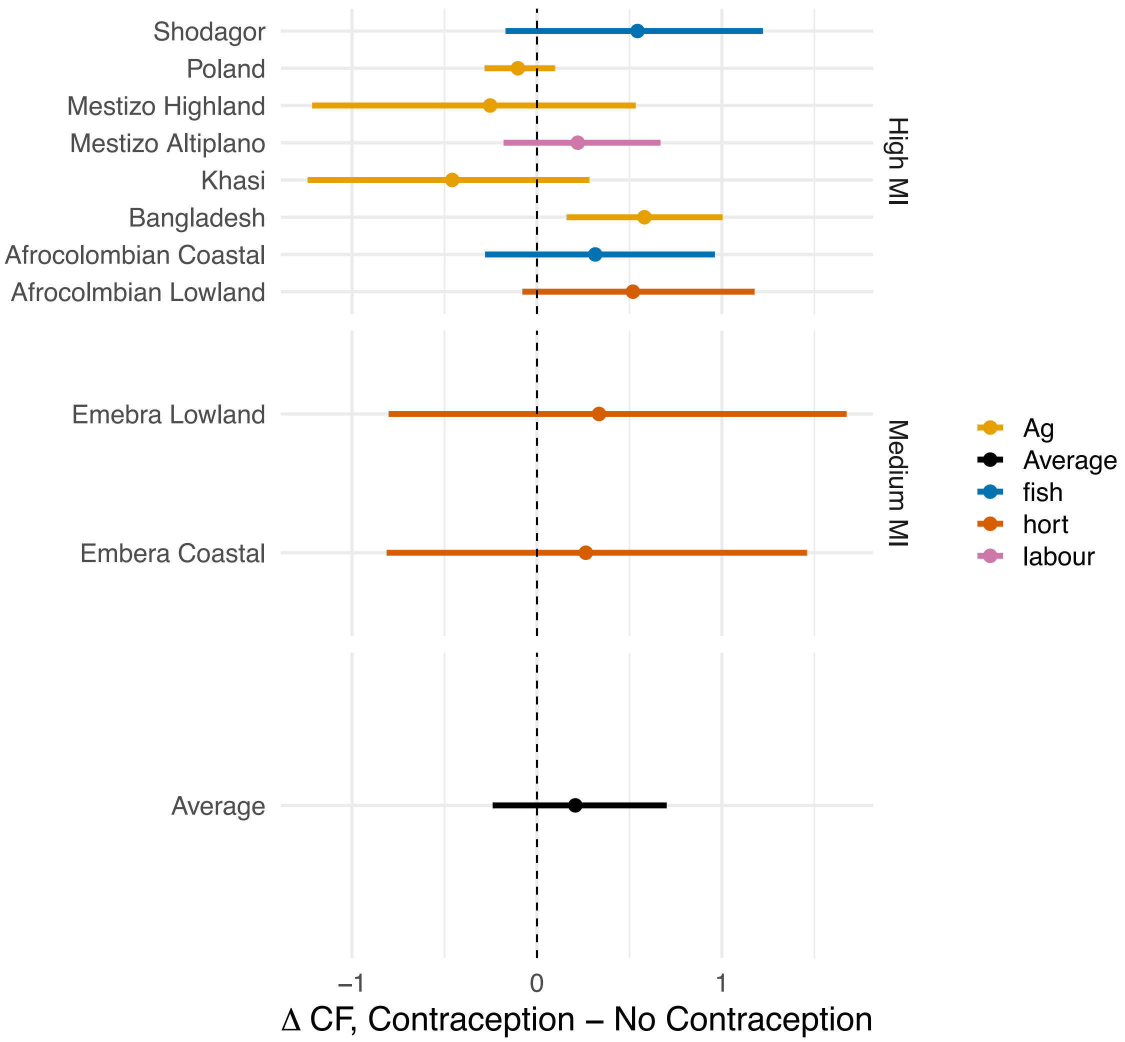

Figure S6 Plot of the relationship between contraception and fertility. The strength of evidence for this result is weak, and the point estimates go both directions. Nonetheless, in line with previous work in subsistence-level, transitional societies, we see that on average, contraception is positively associated with fertility. This is particularly the case in less market integrated groups. As documented elsewhere, this is the result of women using biomedical contraceptive to cease reproduction when they had already had a number of children.

Model checks

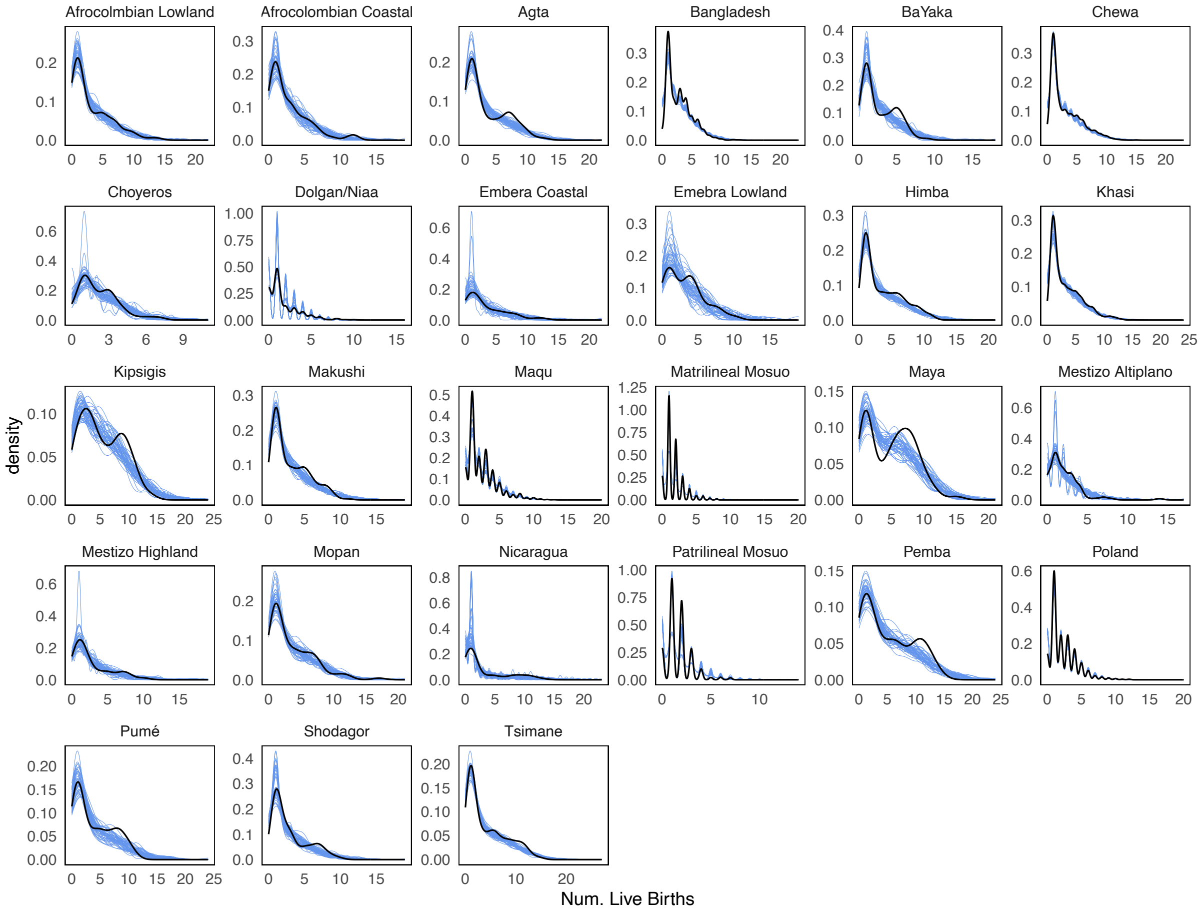

Figure S7: Posterior predictive checks for the 27 populations included in the model predicting fertility cross-culturally (with no subsistence or market-integration predictors included). Highlights that a few (e.g. the Dolgan) populations demonstrate under-dispersion (the observed variation in fertility is lower than predicted by the model). However, there is a good fit of the model in general across populations.

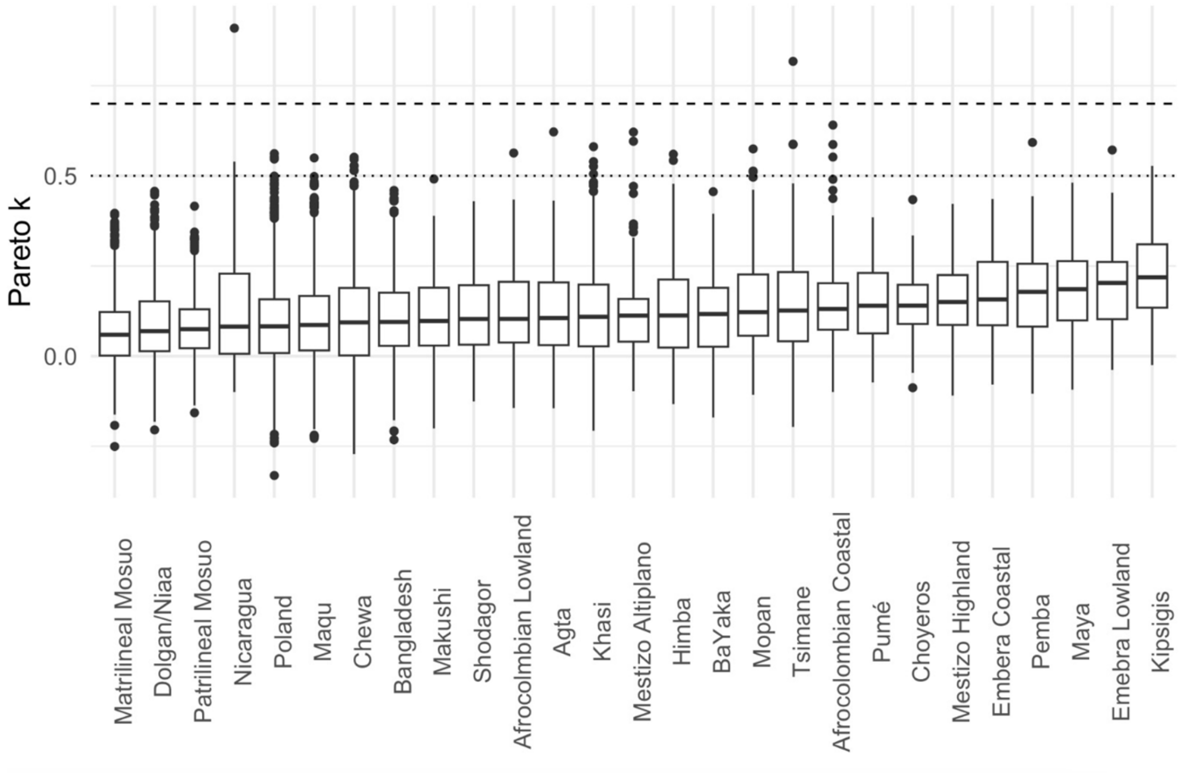

Figure S8: Distribution of Pareto K values by population on the posterior. K > 0.7 is considered highly influential, values 0.5 < K < 0.7 is considered moderately influential. This highlights that no one population is driving the overall relationship between fertility and subsistence, as no population has a cluster of hugely influential observations.

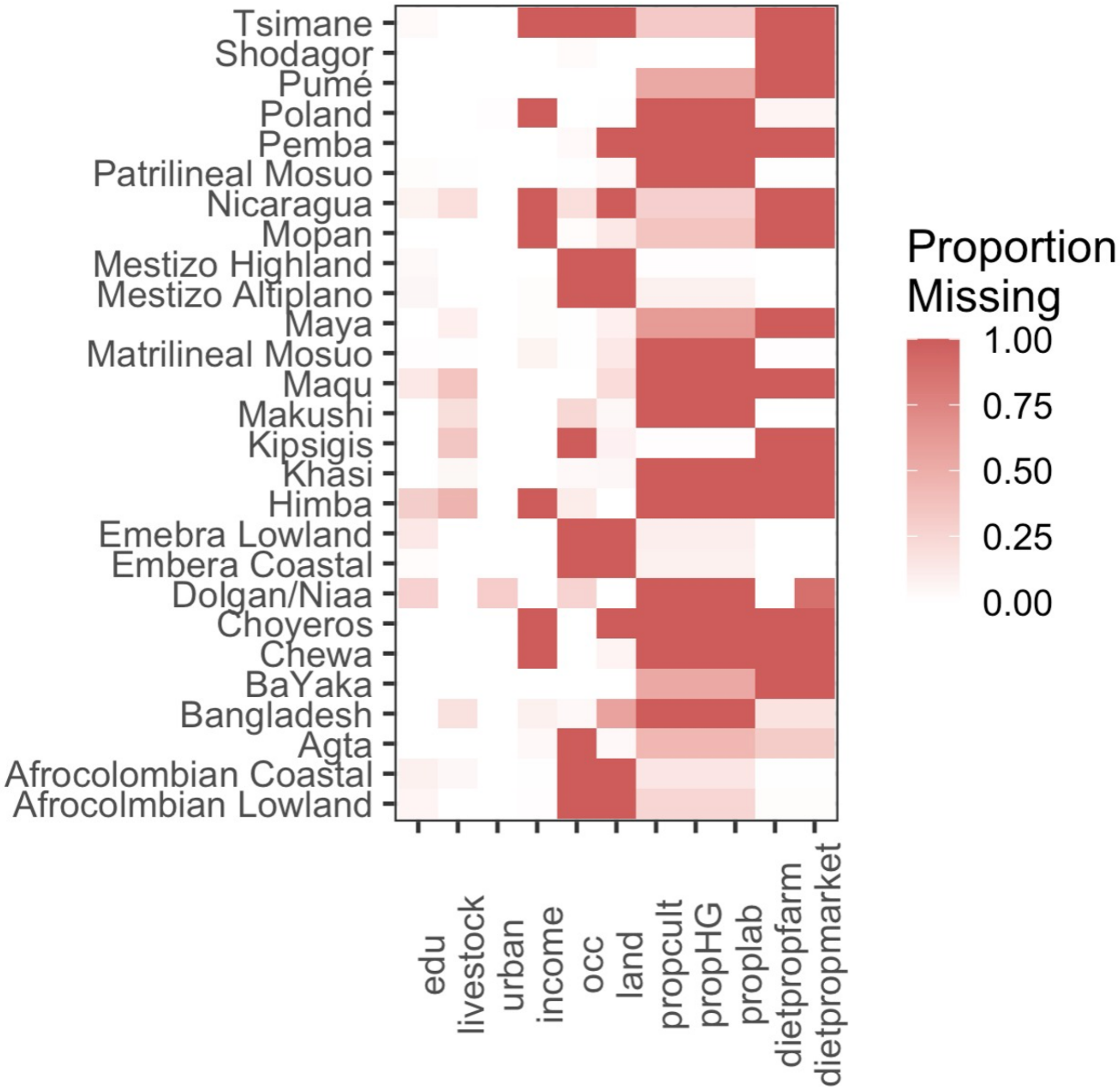

Figure S9: clustering of missingness for all 27 populations across 11 predictors. When populations have fully missing data this represents both when the data was never collected, or the measure is not applicable to the population (e.g., occupation in the Agta).

SI References

1. T. Minter, “The Agta of the Northern Sierra Madre: Livelihood strategies and resilience among Philippine hunter-gatherers,” Leiden University. (2010).

2. M. Dyble, J. Thorley, A. E. Page, D. Smith, A. B. Migliano, Engagement in agricultural work is associated with reduced leisure time among Agta hunter-gatherers. *Nat. Hum. Behav.* (2019) https:/doi.org/10.1038/s41562-019-0614-6.

3. A. E. Page, *et al.*, Reproductive trade-offs in extant hunter-gatherers suggest adaptive mechanism for the Neolithic expansion. *Proc. Natl. Acad. Sci.* **113**, 4694-4699. (2016).

4. A. E. Page, T. Minter, S. Viguier, A. B. Migliano, Hunter-gatherer health and development policy: How the promotion of sedentism worsens the Agta’s health outcomes. *Soc. Sci. Med.* **197**, 39–48 (2018).

5. M. Dyble, *et al.*, Sex equality can explain the unique social structure of hunter-gatherer bands. *Science* **348**, 796–798 (2015).

6. B. M. Griffin, The Cultural Identity of Foragers and the Agta of Palanan, Isabela the Philippines. *Anthropos* **91**, 111–123 (2012).

7. J. T. Peterson, *The Ecology of Social Boundaries: Agta Foragers of the Philippines* (University of Illinois Press, 1978).

8. M. Dyble, *et al.*, Networks of Food Sharing Reveal the Functional Significance of Multilevel Sociality in Two Hunter-Gatherer Groups. *Curr. Biol.* **26**, 2017–2021 (2016).

9. A. E. Page, *et al.*, Testing adaptive hypotheses of alloparenting in Agta foragers. *Nat. Hum. Behav.* **3**, 1154–1163 (2019).

10. A. B. Migliano, *et al.*, Characterization of hunter-gatherer networks and implications for cumulative culture. *Nat. Hum. Behav.* **1**, 1–6 (2017).

11. A. E. Page, *et al.*, Children are important too: juvenile playgroups and maternal childcare in a foraging population, the Agta. *Philos Trans R Soc Lond B Biol Sci* **376**, 20200026 (2021).

12. Y. Diekmann, *et al.*, Accurate age estimation in small-scale societies. *Proc. Natl. Acad. Sci.*, 1619583114 (2017).

13. T. Minter, “The Agta of the Northern Sierra Madre,” Lieden University. (2008).

14. J. Lewis, Forest hunter-gatherers and their world: a study of the Mbendjele Yaka pygmies of Congo-Brazzaville and their secular and religious activities and representations. 1–311 (2002).

15. B. S. Hewlett, *Intimate fathers: The nature and context of Aka Pygmy paternal infant care* (University of Michigan Press, 1993).

16. J. Lewis, Ekila: Blookd, bodies, and egalitarian societies. *J. R. Anthropol. Inst.* **14**, 297–315 (2008).

17. K. Kitanishi, Food Sharing among the Aka Hunter-Gatherers in Northeastern Congo. *Afr. Study Monogr.* **25**, 3–32 (1998).

18. B. S. Hewlett, H. N. Fouts, A. H. Boyette, B. L. Hewlett, Social learning among Congo Basin hunter-gatherers. *Philos. Trans. R. Soc. Lond. B. Biol. Sci.* **366**, 1168–1178 (2011).

19. B. S. Hewlett, J. Hudson, A. H. Boyette, H. N. Fouts, "Intimate living: sharing space among Aka and other hunter-gatherers" in *Towards a Broader View of Hunter-Gatherer Sharing*, N. Lavi, D. E. Friesem, Eds. (McDonald Institute for Archaeological Research, 2019), pp. 39–52.

20. K. Kitanishi, Seasonal changes in the subsistence activities and food intake of the Aka hunter-gatherers in northeastern Congo. *Afr. Study Monogr.* **16**, 73–118 (1995).

21. S. Bahuchet, H. Guillaume, Aka-farmer relations in the northwest Congo Basin. *Polit. Hist. Band Soc.*, 189–211 (1982).

22. R. R. Grinker, *Houses in the Rainforest* (University of California Press, 1994).

23. K. Takeuchi, "Interethnic relationships between pygmies and farmers" in *Hunter-Gatherers of the Congo Basin: Cultures, Histories and Biology of African Pygmies*, B. S. Hewlett, Ed. (Transaction Publishers, 2014), pp. 299–320.

24. D. Bombjaková, S. Lew-Levy, R. Duda, G. Loubelo, J. Lewis, BaYaka education: From the forest to the ORA (Observer, Réflechir, Agir) classroom. *Hunt. Gatherer Res.*

25. A. H. Boyette, S. Lew-Levy, H. Jang, V. Kandza, Social ties in the Congo Basin: insights into tropical forest adaptation from BaYaka and their neighbours. *Philos. Trans. R. Soc. B Biol. Sci.* **377**, 20200490 (2022).

26. K. A. Klieman, *The Pygmies were our compass: Bantu and Batwa in the History of West Central Africa, Early Times to C. 1900 C.E.* (Greenwood, 2003).

27. M. Riddell, Assessing the impacts of conservation and commercial forestry on livelihoods in Northern Republic of Congo. *Conserv. Soc.* **11**, 199–217 (2013).

28. V. H. Kandza, H. Jang, K. Ntamboudila, S. Lew-Levy, A. H. Boyette, Inter-group cooperation in shotgun hunting among BaYaka foragers and Yambe farmers from the Republic of the Congo. *Hum. Nat.*

29. J. P. Ziker, *Peoples of the tundra : northern Siberians in the post-communist transition* (Waveland Press, Inc., 2002).

30. J. P. Ziker, Raw and Cooked in Arctic Siberia: Diet and Consumption Strategies in Socio-ecological Perspective. *Nutr. Anthropol.* **25**, 20–33 (2002).

31. J. P. Ziker, “Subsistence and Sharing in Northern Siberia: Experimental Economics with the Dolgan and the Nganasan” in *Experimenting with Social Norms: Fairness and Punishment in Cross Cultural Perspective*, J. Ensminger, J. Henrich, Eds. (Russell Sage Foundation Publications, 2014), pp. 337–356.

32. J. P. Ziker, “Subsistence and residence in the Putoran uplands and Taimyr lowlands in 1926-27” in *The 1926/27 Soviet Polar Census Expeditions*, (Berghahn Books, 2011), pp. 244–267.

33. J. P. Ziker, “Microdemographics and indigenous identity in the central Taimyr lowlands” in *Indigenous Peoples and Demography: The Complex Relation between Identity and Statistics*, P. Axelsson, P. Sköld, Eds. (Berghahn Books, 2011), pp. 219–237.

34. J. P. Ziker, Assigned territories, family/clan/communal holdings, and common-pool resources in the Taimyr Autonomous Region, Northern Russia. *Hum. Ecol.* **31**, 331–368 (2003).

35. J. P. Ziker, J. Rasmussen, D. A. Nolin, Indigenous Siberians solve collective action problems through sharing and traditional knowledge. *Sustain. Sci.* **11**, 45–55 (2016).

36. J. Ziker, M. Schnegg, Food sharing at meals: Kinship, reciprocity, and clustering in the Taimyr Autonomous Okrug, Northern Russia. *Hum. Nat.* **16**, 178–210 (2005).

37. J. P. Ziker, K. S. Fulk, Paying It Forward or Giving Back? Women’s Sharing Networks in Siberia. *Cross-Cult. Res.* **53**, 272–290 (2019).

38. D. A. Nolin, J. P. Ziker, Reproductive Responses to Economic Uncertainty: Fertility Decline in Post-Soviet Ust’-Avam, Siberia. *Hum. Nat.* **27**, 351–371 (2016).

39. J. Henrich, *et al.*, Costly punishment across human societies. *Science* **312**, 1767–1770 (2006).

40. J. Henrich, *et al.*, Markets, religion, community size, and the evolution of fairness and punishment. *Science* **327**, 1480–1484 (2010).

41. J. S. Malan, *Peoples of Namibia* (1995).

42. S. P. Prall, B. A. Scelza, The dietary impacts of drought in a traditional pastoralist economy. *Am. J. Hum. Biol.* (2022).

43. M. Bollig, “Inheritance and Maintenance among the Himba of the Kunene Region” in *Perspectives on Namibian Inheritance Practices*, (2005), p. 45.

44. B. A. Scelza, Female mobility and postmarital kin access in a patrilocal society. *Hum. Nat.* **22**, 377–393 (2011).

45. B. A. Scelza, *et al.*, High rate of extrapair paternity in a human population demonstrates diversity in human reproductive strategies. *Sci. Adv.* **6**, 1–7 (2020).

46. M. Borgerhoff Mulder, T. M. Caro, The use of quantitative observational techniques in anthropology. *Curr. Anthropol.* **26**, 323–335 (1985).

47. M. Borgerhoff Mulder, "Time allocation among the Kipsigis of Kenya." in *Cross Cultural Studies in Time Allocation*, (Human Relations Area Files, Inc., 1997).

48. M. Borgerhoff Mulder, On cultural and reproductive success: Kipsigis evidence. American Anthropologist. *Am. Anthr.* **89**, 617–634 (1987).

49. M. Borgerhoff Mulder, Resources and reproductive success in women, with an example from the Kipsigis. *J. Zool.* **213**, 489–505 (1987).

50. R. Schacht, "Cassava and the Makushi: a shared history of resiliency and transformation" in *Food and Identity in the Caribbean*, (Berg Publishers, 2013), pp. 15–29.

51. I. Myers, The Makushi of the Guiana-Brazilian frontier in 1944: a study of culture contact. *Anthrpologica* **80**, 3–99 (1993).

52. J. A. Forte, *About Guyanese Amerindians* (University of Guyana Press, 1996).

53. R. Schacht, M. Borgerhoff Mulder, Sex ratio effects on reproductive strategies in humans. *R. Soc. Open Sci.* **2**, 140402 (2015).

54. J. Gafar, The performance of the labour market in Guyana. *Can. J. Dev. Stud.* **25**, 663–685 (2004).

55. K. L. Kramer, Children's help and the pace of reproduction: Cooperative breeding in humans. *Evol. Anthropol.* **14**, 224–237 (2005).

56. K. L. Kramer, J. V Hackman, R. Schacht, H. E. Davis, Effects of family planning on fertility behaviour across the demographic transition. *Sci. Rep.* **11**, 1–12 (2021).

57. D. J. Holman, K. A. O'Connor, "Bangladeshis" in *Encyclopedia of Medical Anthropology*, C. Ember, M. Ember, Eds. (Kluwer Academic/Plenum Publishers, 2004), pp. 579–590.

58. ICDDRB, "Health and Demographic Surveillance System—Matlab. 2005 Socioeconomic census" (2007).

59. N. Kabeer, Ideas, economics and the 'sociology of supply': Explanations for fertility decline in Bangladesh. *J. Dev. Stud.* **1**, 29–70 (2001).

60. J. J. Nowak, *Bangladesh: reflections on the water* (Indiana University Press, 1993).

61. I. M. Fund, "World economic outlook database" (2012).

62. A. Razzaque, P. K. Streatfield, A. Evans, Family size and children's education in Matlab, Bangladesh. *J. Biosoc. Sci.* **39**, 245–256 (2007).

63. K. M. A. Aziz, *Kinship in Bangladesh* (nternational Centre for Diarrhoeal Disease Research, Bangladesh, 1979).

64. ICDDRB, "Health and Demographic Surveillance System—Matlab" (2012).

65. M. K. Shenk, K. Starkweather, H. C. Kress, N. Alam, Does absence matter? : a comparison of three types of father absence in rural bangladesh. *Hum. Nat.* **24**, 76–110 (2013).

66. B. Esteve-Volart, "Dowry in rural Bangladesh: participation as insurance against divorce" (2004).

67. B. K. Caldwell, Factors affecting female age at marriage in South Asia. *Asian Popul. Stud.* **1**, 283–301 (2005).

68. M. K. Shenk, *et al.*, Social support , nutrition and health among women in rural Bangladesh : complex tradeoffs in allocare , kin proximity and support network size. *Philos. Trans. R. Soc. B Biol. Sci.* **376** (2021).

69. G. Perry, Alloparental care and assistance in a normatively patrilocal society. *Curr. Anthropol.* **58**, 114–123 (2017).

70. ICDDRB, "Health and Demographic Surveillance System - Matlab, v. 54" (2021).

71. M. A. Koenig, Trends in family size preferences and contraceptive use in Matlab, Bangladesh. *Stud. Fam. Plann.* **18**, 117–127 (1987).

72. M. A. Koenig, Contraceptive use in Matlab, Bangladesh in 1990: levels, trends, and explanations. *Stud. Fam. Plann.* **23**, 352–64 (1992).

73. S. Joshi, "Female House-heaedship in Rural Banladesh: Incidence, Determinants, and Impact on Children's Schooling. Economic Growth Center" (2004).

74. N. Alam, Teenage motherhood and infant mortality in Bangladesh: maternal age-dependent effect of parity one. *J. Biosoc. Sci.* **32**, 229–36 (2000).

75. J. K. Knight, *et al.*, Quantifying patterns of alcohol consumption and its effects on health and wellbeing among BaYaka hunter-gatherers: A mixed-methods cross-sectional study. *PLoS ONE* **16**, e0258384 (2021).

76. N. Chaudhary, *et al.*, Polygyny without wealth: popularity in gift games predicts polygyny in BaYaka Pygmies. *R. Soc. Open Sci.* **2**, 150054 (2015).

77. N. Chaudhary, *et al.*, Competition for Cooperation: variability, benefits and heritability of relational wealth in hunter-gatherers. *Sci. Rep.* **6**, 29120 (2016).

78. G. D. Salali, *et al.*, Global WEIRDing: Transitions in wild plant knowledge and treatment preferences in Congo hunter-gatherers. *Evol. Hum. Sci.* **2** (2020).

79. G. D. Salali, A. B. Migliano, Future Discounting in Congo Basin Hunter-Gatherers Declines with Socio-Economic Transitions. *PLoS ONE* **10**, 1–10 (2015).

80. L. Pacheco-Cobos, B. P. Winterhalder, Ethnographic Observations on the Role of Domestic Dogs in the Lowland Tropics of Belize with Emphasis on Crop Protection and Subsistence Hunting. *Hum. Ecol.* **49**, 779–794 (2021).

81. C. Atwater, C. J. Cortez, L. Pacheco-Cobos, E. Chevez, B. P. Winterhalder, "Household Demographic Census for Santa Cruz, Toledo, Belize (DATASET). University of California Davis / National Science Foundation, Human Systems Dynamics, Collaborative Research. Development and Resilience of Complex Socioeconomic Systems: A Theoretical" (2012).

82. C. Atwater, *et al.*, "Time Allocation for Santa Cruz, Toledo, Belize (DATASET). University of California Davis / National Science Foundation, Human Systems Dynamics, Collaborative Research. Development and Resilience of Complex Socioeconomic Systems: A Theoretical Model and Ca" (2012).

83. E. R. Walsh, The Na. *Encycl. Sex Gend. Men Women Worlds Cult.* (2004).

84. C.-K. Shih, *Quest for Harmony: The Moso Traditions of Sexual Union & Family Life* (Stanford University Press, 2010).

85. C. Mathieu, *A History and Anthropological Study of the Ancient Kingdoms of the Sino-Tibetan Borderland - Naxi and Mosuo* (The Edwin Mellen Press, 2003).

86. S. M. Mattison, *et al.*, Using evolutionary theory to hypothesize a transition from patriliny to matriliny and back again among the ethnic Mosuo of Southwest China. *Matrix J. Matricul. Stud.* **2**, 90–117 (2021).

87. C. Shih, "The Yongning Moso: sexual union, household organization, gender and ethnicity in a matrilineal duolocal society in Southwest China," Stanford University, Palo Alto, CA. (1993).

88. S. M. Mattison, *et al.*, Gender differences in social networks based on prevailing kinship norms in the Mosuo of China. *Soc. Sci.* **10**, 253 (2021).

89. S. M. Mattison, B. Beheim, B. Chak, P. Buston, Offspring sex preferences among patrilineal and matrilineal Mosuo in Southwest China revealed by differences in parity progression. *R. Soc. Open Sci.* **3**, 160526 (2016).

90. E. R. Walsh, From Nü Guo to Nü'er Guo: Negotiating desire in the land of the Mosuo. *Mod. China* **31**, 448–486 (2005).

91. S. M. Mattison, Economic impacts of tourism and erosion of the visiting system among the Mosuo of Lugu Lake. *Asia Pac. J. Anthropol.* **11**, 159–176 (2010).

92. S. M. Mattison, *et al.*, Context Specificity of "Market Integration" among the Matrilineal Mosuo of Southwest China. *Curr. Anthropol.* **63**, 118–124 (2022).

93. J. Koster, M. N. Grote, B. Winterhalder, Effects on Household Labor of Temporary Out-migration by Male Household Heads in Nicaragua and Peru: An Analysis of Spot-check Time Allocation Data Using Mixed-effects Models. *Hum. Ecol.* **41**, 221–237 (2013).

94. J. Koster, *et al.*, Kinship ties across the lifespan in human communities. *Philos. Trans. R. Soc. B Biol. Sci.* **374**, 20180069 (2019).

95. J. Winking, J. Koster, The Fitness Effects of Men's Family Investments: A Test of Three Pathways in a Single Population. *Hum. Nat.* **26**, 292–312 (2015).

96. J. Winking, J. Koster, Timing, Initiators, and Causes of Divorce in a Mayangna/Miskito Community in Nicaragua. *Soc. Sci.* **10**, 212 (2021).

97. C. M. Beall, P. W. Lesile, Collecting women's reproductive histories. *Am. J. Hum. Biol.* **26**, 577–589 (2014).

98. K. Kramer, R. Greaves, Changing Patterns of Infant Mortality and Maternal Fertility among Pumé Foragers and Horticulturalists. *Am. Anthropol.* **109**, 713–726 (2007).

99. K. L. Kramer, R. D. Greaves, "Diversity or Replace. What happens to wild foods when cultigens are introduced into hunter-gatherer diets" in *Why Forage? Hunters and Gatherers Living in the 21st Century*, B. F. Codding, K. L. Kramer, Eds. (School of Advanced Research, Santa Fe, 2016), pp. 15–42.

100. S. J. Macfarlan, *et al.*, NDVI predicts birth seasonality in historical Baja California Sur, Mexico: Adaptive responses to arid ecosystems and the North American Monsoon. *Biodemography Soc. Biol.* **66**, 145–155 (2021).

101. S. J. Macfarlan, *et al.*, Marriage dynamics in old Lower California: Ecological constraints and reproductive value in an arid peninsular frontier. *Biodemography Soc. Biol.* **65**, 156–171 (2019).

102. S. J. Macfarlan, *et al.*, The role of dispersal and school attendance on reproductive dynamics in small, dispersed populations: Choyeros of Baja California Sur, Mexico. *PLoS One* **15**, e0239523 (2020).

103. S. J. Macfarlan, R. Schacht, C. A. Davis, Decision-making under climate shocks and economic insecurity: Ranching in rural Baja California Sur, Mexico. *Evol. Hum. Behav.*

104. J. Lerback, B. Bowen, S. J. Macfarlan, E. Schniter, J. J. Garcia, Development of a graphical resilience framework to understand a coupled human-natural system in a remote arid highland of Baja California Sur. *Sustain. Sci.* (2022) https:/doi.org/https://doi.org/10.1007/s11625-022-01101-6.

105. L. McAllister, M. Gurven, H. Kaplan, J. Stieglitz, Why do women have more children than they want? Understanding differences in women's ideal and actual family size in a natural fertility population. *Am. J. Hum. Biol.* **24**, 786–799 (2012).

106. M. D. Gurven, Infant and fetal mortality among a high fertility and mortality population in the Bolivian Amazon. *Soc. Sci. Med.* **75**, 2493–2502 (2012).

107. M. A. Martin, G. Garcia, H. S. Kaplan, M. D. Gurven, Conflict or congruence? Maternal and infant-centric factors associated with shorter exclusive breastfeeding durations among the Tsimane. *Soc. Sci. Med.* **170**, 9–16 (2016).

108. C. von Rueden, M. Gurven, H. Kaplan, The multiple dimensions of male social status in an Amazonian society. *Evol. Hum. Behav.* **29**, 402–415 (2008).

109. R. a. Godoy, *et al.*, Do markets worsen economic inequalities? Kuznets in the Bush. *Hum. Ecol.* **32**, 339–364 (2004).

110. N. Howell, *Demography of the Dobe !Kung* (Aldine, 1979).

111. K. Hill, A. M. Hurtado, *Aché Life History: The Ecology and Demography of a Foraging People* (Aldine de Gruyter, 1996) https:/doi.org/10.1016/S1090-5138(97)00035-4.

112. N. G. Blurton Jones, K. Hawkes, J. F. O'Connell, Antiquity of postreproductive life: Are there modern impacts on hunter-gatherer postreproductive life spans? *Am. J. Hum. Biol.* **14**, 184–205 (2002).

113. M. D. Gurven, *et al.*, The Tsimane Health and Life History Project: Integrating anthropology and biomedicine. *Evol. Anthropol.* **26**, 54–73 (2017).

114. L. von Bertalanffy, Untersuchungen über die Gesetzlichkeit des Wachstums. *Wilhelm RouxArchiv Für Entwicklungsmechanik Org.* **131**, 613–652 (1934).

115. J. Koster, *et al.*, The life history of human foraging: Cross-cultural and individual variation. *Sci. Adv.* **6**, 1–8 (2020).

116. S. Lew-Levy, *et al.*, The life history of learning subsistence skills among Hadza and BaYaka foragers from Tanzania and the Republic of Congo. *Hum. Nat.* **32**, 16–47 (2021).

117. I. Pretelli, E. J. Ringen, S. Lew-Levy, Foraging complexity and the evolution of childhood. *EcoEvoRxiv Prepr.* (2022).

118. R Core Team, 2021 R: a language and environment for statistical coding. *Vienna Austria R Found. Stat. Comput.*

119. S. D. Team, RStan (2022).
